# EAGS: efficient and adaptive Gaussian smoothing applied to high-resolved spatial transcriptomics

**DOI:** 10.1093/gigascience/giad097

**Published:** 2024-02-20

**Authors:** Tongxuan Lv, Ying Zhang, Mei Li, Qiang Kang, Shuangsang Fang, Yong Zhang, Susanne Brix, Xun Xu

**Affiliations:** BGI Research, Shenzhen 518083, China; College of Life Sciences, University of Chinese Academy of Sciences, Beijing 100049, China; BGI Research, Shenzhen 518083, China; BGI Research, Shenzhen 518083, China; Department of Biotechnology and Biomedicine, Technical University of Denmark, 2800 Kgs. Lyngby, Denmark; BGI Research, Shenzhen 518083, China; BGI Research, Shenzhen 518083, China; BGI Research, Beijing 102601, China; BGI Research, Shenzhen 518083, China; BGI Research, Beijing 102601, China; BGI Research, Shenzhen 518083, China; College of Life Sciences, University of Chinese Academy of Sciences, Beijing 100049, China

**Keywords:** spatial transcriptomics, imputation, gaussian smoothing, adaptive weight

## Abstract

**Background:**

The emergence of high-resolved spatial transcriptomics (ST) has facilitated the research of novel methods to investigate biological development, organism growth, and other complex biological processes. However, high-resolved and whole transcriptomics ST datasets require customized imputation methods to improve the signal-to-noise ratio and the data quality.

**Findings:**

We propose an efficient and adaptive Gaussian smoothing (EAGS) imputation method for high-resolved ST. The adaptive 2-factor smoothing of EAGS creates patterns based on the spatial and expression information of the cells, creates adaptive weights for the smoothing of cells in the same pattern, and then utilizes the weights to restore the gene expression profiles. We assessed the performance and efficiency of EAGS using simulated and high-resolved ST datasets of mouse brain and olfactory bulb.

**Conclusions:**

Compared with other competitive methods, EAGS shows higher clustering accuracy, better biological interpretations, and significantly reduced computational consumption.

## Introduction

Recent advances in barcode-based spatial transcriptomics (ST) technology include 10X Visium [1], Slide-Seq [2, 3], and high-definition spatial transcriptomics [4]. These advances made it feasible to provide expression profile information of entire genes, which is extremely important for comprehending biological functions and interaction networks [5, 6]. High-resolved ST is an essential technical support for analyzing complex biological problems, as the function of complex biological tissues is closely related to the location of the transcriptional expression events within the tissue. However, cell localization and identification are limited by technical factors, such as the chip capture area, the sequencing depth, and the resolution. Spatially enhanced resolution transcriptome sequencing (Stereo-seq) [7] is a new ST technology based on DNA nanoballs. Stereo-seq provides the highest resolution (500 nm) among all currently available ST technologies. Such breakthrough in resolution allows researchers to perform genome-wide analyses of gene expression at the capture site (spot) with a single-cell or even subcellular resolution. Wang et al. [8] applied Stereo-seq to the 3-dimensional reconstruction of the ST of *Drosophila* embryos and larvae, providing a spatial- and temporal-resolved transcriptomic map of the whole organism across the developmental stages for *Drosophila* research. Liu et al. [9] reconstructed the developmental trajectory of zebrafish embryos during their development by analyzing Stereo-seq and single-cell RNA sequencing (scRNA-seq) datasets from different time points.

Barcode-based high-resolved ST technology captures fewer genes at a single sequencing site (spot) than low-resolution ST technologies, such as 10X Visium [1], leading to high sparsity of the complete gene expression profile. In certain cell cycle phases, some cells do not express a set of genes whose expression thus appears to be null. In addition, amplification bias, cell cycle, library creation, and poor RNA capture rates cause some genes to be expressed but not captured by DNA nanoballs; such genes are called “dropout” [10]. Such biases adversely affect downstream analyses, such as clustering, cellular interaction analyses, and pseudo-temporal reconstructions [11, 12], when the raw data are directly processed.

Various imputation methods have been proposed to solve the “dropout” in gene expression for scRNA-seq datasets [13]. These imputation methods can be broadly classified into 3 categories according to their principles. The first category smooths or diffuses the levels of gene expression in cells with comparable expression patterns to correct (typically) all values (zero and nonzero). MAGIC imputes the missing data on scRNA-seq datasets based on the Markov chains of adjacent domains and recovers gene expression of the characterized cells by data diffusion [14]; DrImpute finds similar cells by consensus clustering and pools their gene expression values to estimate the loss [15]. The second category models the gene expression profile with an existing probabilistic statistical model to simulate the distribution of genes. SAVER assumes that each gene in each cell follows a Poisson–Gamma distribution (a negative binomial distribution) and estimates prior parameters to recover the expression of the missing genes using Poisson LASSO regression methods [16]. Scimpute constructs a mixed Gamma–Normal distribution based on the gene expression profile and uses a nonnegative least squares regression model, sc-transform (R package), to perform the imputation [17]. The third category uses deep learning methods to capture the potential spatial representation of cells and reconstruct the expression matrix. DCA is an auto-encoder that predicts the parameters of the selected distribution to generate estimates [18]. These methods offer practical recommendations for single-cell imputation; however, these methods do not account for spatial information in ST datasets, and the methods based on specialized statistical models cannot be applied to the high sparsity of high-resolved ST datasets.

In recent years, ST-based imputation methods have been presented. Sprod first projects gene expression onto a potential space, connects nearest neighbor cells to construct patterns, and then learns the denoising matrix using a shared minimization of the graph's Laplacian smoothing term and reconstruction errors [19]. For ST data without pathology images, Sprod provides cluster-based pseudo-images, but it does not accurately reflect the actual cell clustering situation. STAGATE introduces a graph attention auto-encoder to construct a spatial neighbor network based on sequencing spots, and then, it introduces a distribution of the spatial neighbor network in the middle layer of the self-encoder to learn the correlation of neighboring sequencing spots and subsequently obtains the recovered gene expression profile by decoder [20]. However, the labels processed based on a specific clustering method are not completely consistent with the reality of the biological organization. It has been noted that the self-attention layer of the network does not consider the interaction between spot pairs and the information about the graphical structure of the spots [21].

To address these problems, we propose an efficient and adaptive Gaussian smoothing (EAGS) method, which is applied to high-resolved ST data. EAGS is derived from the fact that the spatial location of cells in biological tissues has a close relationship with their microenvironment, and the gene expression levels of cells within the same microenvironment are similar [17, 22]. EAGS constructs different patterns based on cell expression profiles and cell location information to generate a similarity matrix. The similarity matrix then assesses cellular similarity within expression profiles to recover true biosignatures. By refining the information from proximal cells using adaptive smoothing weights and generating new gene expression profiles, the “dropout” is reduced. The resulting dataset provides RNA abundances more accurately than the original gene expression profile and preserves more of the true biological signal. EAGS enables the usage of high-sparsity ST datasets since it is independent of prior statistical models of the expression preconditioning the gene expression profiles. More crucially, EAGS could be used for large-scale ST datasets without requiring a lot of operating memory since it does not call for the computation of parameters for a predefined model, skipping most of the iterative process. We applied EAGS to the simulated and high-resolved ST datasets of mouse brain and olfactory bulb and compared it with widely used imputation methods to evaluate its efficacy in terms of fewer “zeros” in the gene expression profiles, improved cell annotation, and spatial organization replication.

## Methods

### The workflow of EAGS

In EAGS (RRID: SCR_024399), the original expression matrix with the single-cell resolution was first used to generate patterns based on expression and spatial information. Then, the tight relationship between cells was established using 2 distinct patterns. Finally, the smoothing weights calculated from the patterns were used to define the level of smoothing for each cell and applied to recalculate the gene expression.

### Datasets

There were 2 methods to generate gene expression profiles from Stereo-seq *in situ* captured data. One was to acquire the spatial location information of various cells by conducting cell identification and segmentation on the optical stained image and then match the cell in the image to the sequencing spots with spatial coordinates [7, 23]. The other one was to take consecutive *X* × *X* bins as units (considered cells), where each bin (bin*X*) contains the total gene expression of *X* × *X* spots [7]. We used the mouse brain [24] and olfactory bulb datasets at single-cell resolution [23], which were generated by the first method and included 61,857 and 33,272 cells, respectively. The *in situ* hybridization (ISH) images of the signature genes from the mouse brain were obtained to help compare the impacts of smoothing [25, 26]. We also used another mouse olfactory bulb dataset generated by the second method, which contained 812 units of Bin140 [27].

The above 2 categories of the gene expression profile with spatial information were preprocessed with the Scanpy toolbox (V1.9.1; RRID:SCR_018139) to remove low-quality signals that might be blended into the gene expression data [28, 29]. For the first category, first, we filtered genes based on expression in at least 10 cells: those genes were kept. Next, cell outliers were filtered using gene expression: cells expressing at least 300 molecular identifier (MID) counts were kept. The 2% highest MID counts in all cells were subtracted from the overall number of MIDs across all cells in the gene expression profile. Finally, the coordinates of the spatial position information of the cells and the log-transformed and normalized gene expression profiles were employed as input to EAGS. For the second category, we filtered genes based on expression in at least 10 cells: those genes were kept. Next, cell outliers were filtered using gene expression: cells expressing at least 300 MID counts were kept.

### Pattern construction

Since “similar cells” in organisms with comparable molecular microenvironments express their genes similarly, the regions with identical expression patterns may originate from the same cell type or from the same biological tissue location [17, 22]. Using “similar cells” to supplement the information of a particular spot is feasible. Based on spatial location data and gene expression profiles, we constructed 2 patterns to divide the cells on an ST slice's gene expression profile into several clusters. A comprehensive description of these 2 pattern styles is given as follows:

Definition 1 **(Gene Expression Pattern)**: If ${{\boldsymbol{P}}}_e( i )$ is the gene expression domain of ${\boldsymbol{Cel}}{{\boldsymbol{l}}}_i$ for ST data, then

(1) \begin{eqnarray*}
\forall {\boldsymbol{Cel}}{{\boldsymbol{l}}}_j \in {{\boldsymbol{P}}}_e\left( i \right),\;\;\forall {\boldsymbol{Cel}}{{\boldsymbol{l}}}_k \in {{\boldsymbol{P}}}_g - \left( {{{\boldsymbol{P}}}_e\left( i \right) \cup \{ {\boldsymbol{Cel}}{{\boldsymbol{l}}}_i\} } \right),\;\;s.t.{d}_{ij}^e < {d}_{ik}^e
\end{eqnarray*}

where ${\boldsymbol{Cel}}{{\boldsymbol{l}}}_i$, ${\boldsymbol{Cel}}{{\boldsymbol{l}}}_j$, and ${\boldsymbol{Cel}}{{\boldsymbol{l}}}_k$ are different cells; ${{\boldsymbol{P}}}_g$ is the global pattern of gene expression; and ${d}_{ij}^e$ and ${d}_{ik}^e$ are the distance between ${\boldsymbol{Cel}}{{\boldsymbol{l}}}_i$ and ${\boldsymbol{Cel}}{{\boldsymbol{l}}}_j$, as well as ${\boldsymbol{Cel}}{{\boldsymbol{l}}}_i$ and ${\boldsymbol{Cel}}{{\boldsymbol{l}}}_k$, respectively.

Balltree is a binary tree data structure that performs well on high-dimensional datasets, especially for fast nearest-neighbor search on high-dimensional datasets [30, 31]. The complete gene expression profile is separated into many different subspaces by Balltree. Then, the Euclidean distances between cells are calculated separately. Assuming the prenormalized gene expression profile still contains *m* cells, the unsupervised nearest-neighbor network toolkit (scikit-learn) is used to extract the *n*-dimensional principal component data and creates the low-dimensional information matrix (${\boldsymbol{LDI}}{{\boldsymbol{M}}}_{( {m,n} )}$) for the gene expression profile, as shown in Algorithm 1 [32]. Then, the neighboring cell matrix is constructed based on the k-nearest-neighbors network (kNN) as in Algorithm 2, forming the expression neighbor matrix (${\boldsymbol{EN}}{{\boldsymbol{M}}}_{(m,m)}$). Different definitions are given depending on whether ${\boldsymbol{Cel}}{{\boldsymbol{l}}}_j$ can be attributed to the gene expression pattern of ${\boldsymbol{Cel}}{{\boldsymbol{l}}}_i$:


(2) \begin{eqnarray*}
EN{M}_{\left( {i,j} \right)}{\mathrm{ = }}\left\{ \begin{array}{@{}l@{}} 1,\;j = i\\[5pt]1,{\boldsymbol{Cel}}{{\boldsymbol{l}}}_j \in {{\boldsymbol{P}}}_e\left( i \right)\\[5pt]0,{\boldsymbol{Cel}}{{\boldsymbol{l}}}_j \notin {{\boldsymbol{P}}}_e\left( i \right) \end{array} \right. \end{eqnarray*}

where $EN{M}_{( {i,j} )}$ defines whether ${\boldsymbol{Cel}}{{\boldsymbol{l}}}_j$ is within the gene expression pattern ${{\boldsymbol{P}}}_e( i )$ of ${\boldsymbol{Cel}}{{\boldsymbol{l}}}_i$; if $EN{M}_{( {i,j} )}$ = 1, ${\boldsymbol{Cel}}{{\boldsymbol{l}}}_j$ belongs to the expression pattern of ${\boldsymbol{Cel}}{{\boldsymbol{l}}}_i$, and if $EN{M}_{( {i,j} )}$ = 0, it does not.

Algorithm 1. Builds the tree structure of Balltree

**Table:** 

Balltree is built using a divide-and-conquer method. Initially, Balltree has only 1 (root) node and all data points are assigned to it. At each step, the partition corresponding to each node is split into 2 subpartitions. For a partition ${p}_i$, the splitting procedure is as follows:
Step 1: Find the centroid of the node points in ${\boldsymbol{LDI}}{{\boldsymbol{M}}}_{( {m,n} )}$. Reducing an *n*-dimensional matrix to a 2-dimensional plane, the centroid of the node is centroid 1.
Step 2: Select the farthest point from centroid 1 in ${p}_i$ as the first (left) child pivot ${p}_i^L$.
Step 3: Select the farthest point from ${p}_i^L$ as the second (right) child pivot ${p}_i^R$.
Step 4: Assign each data point ${p}_i$ to the partition whose pivot is closer.
Step 5: Assign the new subpartitions as children of ${v}_i$ in Balltree, i.e., ${v}_i^R$ and ${v}_i^L$.

Algorithm 2. Using Balltree to find the nearest neighbor of each cell

**Table:** 

Input: Balltree structure $nbrs$, nearest neighbor num $k$, test point $t$, Current node $n$
Output: Expression neighbor matrix (${\boldsymbol{ENM}}$)
Algorithm :$ball - tree - research(nbrs,k,t,n)$
if $distance( {t,node.pivot} )$—$node.radius$ ≥$max( q )$:
return;
if node in leaf-node set:
Add $node.pivot$ to $Q$ refresh $q$
If $length( Q )$ > $k$:
Remove the point furthest from the test point
Refresh $q$
else:
$ball - tree - research(nbrs,k,t,node.son1)$
$ball - tree - research(nbrs,k,t,node.son2)$
end if
return ${\boldsymbol{ENM}}$

The difference between ST and scRNA-seq datasets is that the ST dataset provides the spatial coordinate position of each sequencing site (spot). After StereoCell processing, ST data are spots with a single-cell resolution where every spot corresponds to a single physical cell with spatial coordinates [23]. Cells in adjacent regions of histological sections are more likely to come from the identical microenvironment and belong to similar or identical cell types than cells from other areas. Therefore, we offer the spatial neighborhood pattern as a reference and classify the cluster of cells that are physically adjacent to a specific cell as its “spatial neighborhoods”:

Definition 2 **(Spatial Neighbor Pattern):** If ${{\boldsymbol{P}}}_s( i )$ is the spatial neighbor pattern of ${\boldsymbol{Cel}}{{\boldsymbol{l}}}_i$ for ST data, then

(3) \begin{eqnarray*}
\forall {\boldsymbol{Cel}}{{\boldsymbol{l}}}_j \in {{\boldsymbol{P}}}_s\left( i \right),\;s.t.{d}_{ij}^s \le {\tau }_s
\end{eqnarray*}

where ${d}_{ij}^S$ is the spatial distance between ${\boldsymbol{Cel}}{{\boldsymbol{l}}}_i$ and ${\boldsymbol{Cel}}{{\boldsymbol{l}}}_j$, and ${\tau }_s$ represents the maximum spatial distance of ${{\boldsymbol{P}}}_s( i )$ of ${\boldsymbol{Cel}}{{\boldsymbol{l}}}_i$.

Since the spatial distribution of the ST dataset is a 2-dimensional plane space, the Euclidean distance can serve as a useful measure of spatial location between cells in a low-dimensional environment. Therefore, the spatial distance matrix (${\boldsymbol{SD}}{{\boldsymbol{M}}}_{( {m,m} )}$) is constructed by computing the Euclidean distance. Furthermore, since ST chips of the Stereo-seq platform vary in size, EAGS fine-tunes the weight value for different chip sizes while calculating Euclidean distances.

### Adaptive weight calculation

Cells can be used as smoothing factors for ${\boldsymbol{Cel}}{{\boldsymbol{l}}}_i$ and must satisfy both the gene expression pattern and the spatial neighbor pattern belonging to ${\boldsymbol{Cel}}{{\boldsymbol{l}}}_i$. A cell acting as the smoothing factor is more similar in gene expression to the smoothed cell than to other cells in the overall expression profile. EAGS defines the nearest-neighbor contribution matrix (${\boldsymbol{NC}}{{\boldsymbol{M}}}_{( {m,m} )}$) for an ST dataset containing $m$ cells as follows:


(4) \begin{eqnarray*}
{\boldsymbol{NC}}{{\boldsymbol{M}}}_{\left( {m,m} \right)} = {\boldsymbol{SD}}{{\boldsymbol{M}}}_{\left( {m,m} \right)} \cdot {\boldsymbol{EN}}{{\boldsymbol{M}}}_{\left( {m,m} \right)}
\end{eqnarray*}

where ${\boldsymbol{NC}}{{\boldsymbol{M}}}_{( {m,m} )}$ is the dot product obtained by multiplying the corresponding elements of the ${\boldsymbol{SD}}{{\boldsymbol{M}}}_{( {m,m} )}$ and ${\boldsymbol{EN}}{{\boldsymbol{M}}}_{( {m,m} )}$ matrices. The nonzero value ${\boldsymbol{NC}}{{\boldsymbol{M}}}_{nonzero}$ part of the ${\boldsymbol{NC}}{{\boldsymbol{M}}}_{( {m,m} )}$ is selected as the parameter for smoothing weights, and ${\boldsymbol{NC}}{{\boldsymbol{M}}}_{nonzero}$ is a *G*-dimensional row vector, where the condition $G \le M \times M$ is satisfied. The ${p}^{th}$ percentile of the ${\boldsymbol{NC}}{{\boldsymbol{M}}}_{nonzero}$ along the specified axis is calculated by the following method:


(5) \begin{eqnarray*}
\left( {G - 1} \right) \times {p}^{th} = c + t
\end{eqnarray*}

where $G$ represents the number of vectors of ${\boldsymbol{NC}}{{\boldsymbol{M}}}_{nonzero}$. and $c$ and $t$ represent the integer and fractional parts of the calculation result, respectively. The distance distribution threshold ($DDT$) is defined as follows:


(6) \begin{eqnarray*}
DDT = \left( {1 - t} \right) \times {\boldsymbol{NC}}{{\boldsymbol{M}}}_{nonzero}\left[ c \right] + t \times {\boldsymbol{NC}}{{\boldsymbol{M}}}_{nonzero}\left[ {c + 1} \right]
\end{eqnarray*}

where the calculated $c$ and $t$ obtain the ${p}^{th}$ percentile $DDT$ along the specified axis of the ${\boldsymbol{NC}}{{\boldsymbol{M}}}_{nonzero}$. The calculation of the adaptive weights is based on the ${\boldsymbol{NC}}{{\boldsymbol{M}}}_{nonzero}$:


(7) \begin{eqnarray*}
{\boldsymbol{G}}{{\boldsymbol{S}}}_{new} = GS\left( {{\boldsymbol{NCM}}} \right) = a \times {e}^{ - \frac{{{{\left( {{\boldsymbol{NCM}} - b} \right)}}^2}}{{2 \times {\mu }^2}}}
\end{eqnarray*}

where ${\boldsymbol{G}}{{\boldsymbol{S}}}_{new}$ is the degree of smoothing information and is an adaptive weight determined by the degree of similarity between the cells in the pattern's framework, and $GS()$ is used to calculate the adaptive weights. The precise smoothing weight contribution between cells is calculated as follows:


(8) \begin{eqnarray*}
\mu = \sqrt { - \frac{{{{\left( {DDT - b} \right)}}^2}}{{2 \times \ln \left( {\frac{{gs}}{a}} \right)}}}
\end{eqnarray*}

where $gs$ is a hyperparameter that characterizes the overall smoothness of the reference gene expression profile, which represents the overall smoothness of the entire chip. For a $1 \times 1$ cm ST chip of the Stereo-seq platform, $gs$ is set to 0.95. $\mu $ is the smooth weight that varies around the $gs$ and characterizes the overall contribution level of cells in both the ${{\boldsymbol{P}}}_e( i )$ and ${{\boldsymbol{P}}}_s( i )$ to ${\boldsymbol{Cel}}{{\boldsymbol{l}}}_i$.

$DDT$ in Equation (6) refers to the similarity distance between cells generated based on the gene expression pattern and the spatial neighbor pattern in the entire gene expression distribution matrix, which is a global benchmark reference for information distribution and can be characterized as the distribution of the gene expression matrix from the overall level. $\mu $, calculated in Equation (8), refers to the standardized parameters of the Gaussian model. The value of $\mu $ calculated by $DDT$ can make the smoothed gene expression matrix more consistent with the preset distribution; such ${\boldsymbol{G}}{{\boldsymbol{S}}}_{new}$ can be measured with the help of some existing expression quantities, and genes are complemented without changing the overall expression profile.

### Smooth

The raw gene expression profile can be processed after ${\boldsymbol{G}}{{\boldsymbol{S}}}_{new}$ and raw expression ${{\boldsymbol{E}}}_{origin}$ have been obtained:


(9) \begin{eqnarray*}
{{\boldsymbol{E}}}_{GS}\left( x \right) = \frac{{\sum\nolimits_{i \in {P}_A\left( i \right)} {{\boldsymbol{G}}{{\boldsymbol{S}}}_{new}\left( {R\left( {i,x} \right)} \right) \times {{\boldsymbol{E}}}_{origin}\left( i \right) + {{\boldsymbol{E}}}_x} }}{{\sum\nolimits_{i \in {P}_A\left( i \right)} {{\boldsymbol{G}}{{\boldsymbol{S}}}_{new}\left( {R\left( {i,x} \right)} \right) + 1} }}
\end{eqnarray*}

where ${{\boldsymbol{E}}}_{GS}$ represents the level of gene expression after adaptive weight smoothing, ${P}_A(i)$ represents all cells in the region where cell *x* is smoothed, and ${{\boldsymbol{E}}}_x$ represents the original gene expression of the smoothed cell. The whole process can be represented by Algorithm 3.

Algorithm 3. Calculate weights and perform smoothing

**Table:** 

Input: Expression neighbor matrix ${\boldsymbol{ENM}}$, spatial distance matrix ${\boldsymbol{SD}}{{\boldsymbol{M}}}_{( {m,m} )}$, origin expression matrix ${{\boldsymbol{E}}}_{origin}$, hyperparameter $gs$
Output: Smooth expression matrix ${{\boldsymbol{E}}}_{( {GS} )}$
Step 1: Calculating the k-nearest-neighbor cell Euclidean distance distribution.
Step 2: Smooth threshold takes the percentile value *x* of the distance distribution and requires a value from 0.2 to 1.
Step 3: Using Equation (6) to back-calculate the magnitude of $\mu $ at this time; preset $gs$ = 0.95.
Step 4: The Gaussian weights at other distances are calculated by substituting $\mu $ values into Equation (7).
Step 5: Reweighted summation based on the newly calculated Gaussian weights and the original expressions.

If relying entirely on the cells in the ${P}_A(i)$ as smoothing factors without using the origin gene expression of the smoothed ${\boldsymbol{Cel}}{{\boldsymbol{l}}}_i$, Equation (10) can be further streamlined as


(10) \begin{eqnarray*}
{{\boldsymbol{E}}}_{GS}\left( x \right) = \frac{{\sum\nolimits_{i \in {P}_A\left( i \right)} {{\boldsymbol{G}}{{\boldsymbol{S}}}_{new}\left( {R\left( {i,x} \right)} \right) \times {{\boldsymbol{E}}}_{origin}\left( i \right)} }}{{\sum\nolimits_{i \in {P}_A\left( i \right)} {{\boldsymbol{G}}{{\boldsymbol{S}}}_{new}\left( {R\left( {i,x} \right)} \right)} }}
\end{eqnarray*}

where ${{\boldsymbol{E}}}_{GS}$ is completely calculated from the expression level of cells in ${P}_A(i)$, regardless of the gene expression of ${\boldsymbol{Cel}}{{\boldsymbol{l}}}_i$.

### Evaluation method

We measured the imputation error by calculating the L2 norm of the difference between the smoothed matrix and ground truth (L2-error) [33]. We used the Calinski–Harabasz Index (CHI) and the Davies–Bouldin Index (DBI) to evaluate the significance of the differences in intraclass and extra-class similarity of the clustering results. We used Moran's *I* and Geary's *C* to calculate the correlation of cellular marker genes in the gene expression space of the data before and after smoothing [34].

### Imputation error by calculating the L2 norm

L2-error is used to compute the difference between 2 matrix vectors by calculating the Euclidean distance between each corresponding element of the 2 matrices separately. A lower L2-error represents a higher degree of similarity between the 2 matrices, indicating that the method performs better. It is defined as follows:


(11) \begin{eqnarray*}
{\mathrm{L2 - error}} = \sqrt {\sum\nolimits_{i = 1}^N {\sum\nolimits_{j = 1}^N {{{\left( {{Y}_{i,j}} \right)}}^2} } } - \sqrt {\sum\nolimits_{i = 1}^N {\sum\nolimits_{j = 1}^N {{{\left( {{X}_{i,j}} \right)}}^2} } }
\end{eqnarray*}

where ${Y}_{i,j}$ represents the reference gene expression matrix, and ${X}_{i,j}$ represents the smoothed gene expression matrix. L2-error is mainly used to compare the difference between the reference expression matrix with “ground-truth counts” and the smoothed expression matrix.

### Calinski–Harabasz Index

The CHI computes the sum of squares of the distances between points in the class and the class center to determine how closely a class is related [35]. The higher the CHI, the higher the similarity between cells of the same type in the cell population, indicating that this method performs better. It is defined as


(12) \begin{eqnarray*}
{\mathrm{CHI}}(k) = \frac{{tr\left( {{{\boldsymbol{B}}}_q} \right)}}{{tr\left( {{{\boldsymbol{W}}}_q} \right)}} \times \left( {\frac{{h - q}}{{q - 1}}} \right) \end{eqnarray*}

where $h$ is the number of training samples, $q$ is the number of categories, ${{\boldsymbol{B}}}_q$ is the between-category covariance matrix, ${{\boldsymbol{W}}}_q$ is the within-category data covariance matrix, and ${\mathrm{tr()}}$ is the trace calculation function.

### Davies–Bouldin Index

The DBI finds the maximum by calculating the quotient of the sum of the average intraclass distances of any 2 classes within the sample set and the distance between the centers of the 2 clusters [36]. The lower the DBI, the higher the similarity between cells of the same type in the cell population, indicating that this method performs better. It is defined as


(13) \begin{eqnarray*}
{\mathrm{DBI}} = \frac{1}{n}\sum\limits_{i = 1}^n {\mathop {\max }\limits_{i \ne j} } \left( {\frac{{{\sigma }_i + {\sigma }_j}}{{d\left( {{{\boldsymbol{c}}}_i,{{\boldsymbol{c}}}_j} \right)}}} \right) \end{eqnarray*}

where $n$ is the number of categories, ${{\boldsymbol{c}}}_i$ is the center of the $ith$ category, ${\sigma }_i$ is the average distance from all points of the $ith$ category to the center, $d( {{{\boldsymbol{c}}}_i,{{\boldsymbol{c}}}_j} )$ is the distance between the center points ${{\boldsymbol{c}}}_i$ and ${{\boldsymbol{c}}}_j$, and $max()$ is the maximum function.

### Moran's *I*

Moran's *I* is a global autocorrelation statistic for certain metrics on a graph. It is commonly used in spatial data analysis to evaluate autocorrelation on 2-dimensional grids [37]. The higher the Moran's *I*, the stronger the spatial autocorrelation of the cell population, indicating that the method performs better. It is defined as


(14) \begin{eqnarray*}
{\mathrm{Moran's\,\, I}} = \left( {\frac{N}{W}} \right) \times \frac{{\sum\nolimits_{i = 1}^N {\sum\nolimits_{j = 1}^N {\left( {{w}_{ij} \times \left( {{x}_i - \bar{x}} \right) \times \left( {{x}_j - \bar{x}} \right)} \right)} } }}{{\sum\nolimits_{i = 1}^N {{{\left( {{x}_i - \bar{x}} \right)}}^2} }}
\end{eqnarray*}

where $N$ is the number of spatial units indexed by $i$ and $j$, $x$ is the variable of interest, $\bar{x}$ is the mean of $x$, ${w}_{ij}$ are the elements of a matrix of spatial weights with zeros on the diagonal, and $W$ is the sum of all ${w}_{ij}$.

### Geary's *C*

Geary's *C* is a measure of spatial autocorrelation that attempts to determine if observations of the same variable are spatially autocorrelated globally (rather than at the neighborhood level) [38]. The lower the Geary's *C*, the stronger the spatial autocorrelation of the cell population, indicating that the method performs better. It is defined as


(15) \begin{eqnarray*}
{\mathrm{Geary^{\prime} s\,\, C}} = \frac{{\left( {N - 1} \right) \times \sum\nolimits_i {\sum\nolimits_j {\left( {{{\boldsymbol{w}}}_{ij} \times \left( {{x}_i - {x}_j} \right)} \right)} } }}{{2 \times {S}_0 \times \sum\nolimits_i {{{\left( {{x}_i - \bar{x}} \right)}}^2} }}
\end{eqnarray*}

where ${{\boldsymbol{w}}}_{ij}$ is the $i{\rm}th$ row of the spatial weight matrix with zeros on the diagonal, and ${S}_0$ is the sum of all the weights.

## Results

### Overview of EAGS

**Figure 1:**
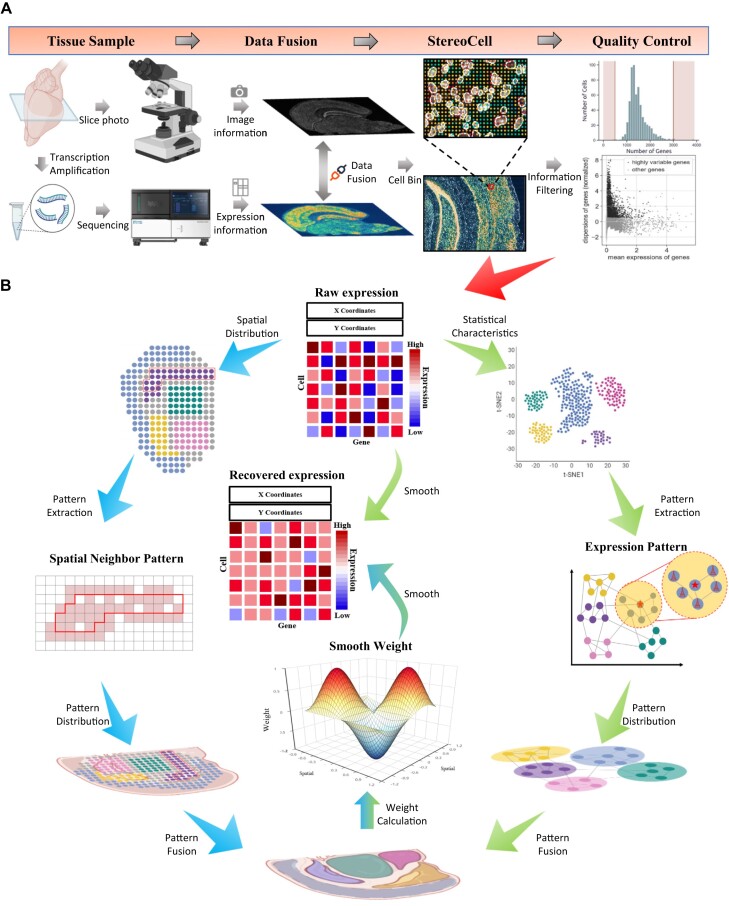
Workflow of EAGS. (A) Data generation process for the input of EAGS. (B) The EAGS method calculates the nearest-neighbor information based on the gene expression pattern and spatial information. Then, EAGS adaptively generates smoothing weights and outputs the smoothed results.

We collect the datasets of mouse brain and olfactory bulb as inputs to EAGS [23, 24]. The acquisition process of these data is as follows: stereo-seq [7] is used to capture the ST data of the mouse brain and mouse olfactory bulb *in situ* and record the position information of the sequencing spot, just like the data generation process in the “Datasets” subsection, and then StereoCell [23] is used to generate ST data at single-cell resolution with spatial information. After obtaining the ST dataset at single-cell resolution, the entire gene expression profile is normalized and smoothed [39], as shown in Fig. 1A.

EAGS constructs 2 styles of patterns based on the input gene expression information and spatial information, respectively. These 2 patterns are used to identify similar cells within the pattern, as shown in Fig. 1B. Next, EAGS adaptively generates smoothing weights based on the difference between similar cells and their genes’ expression, then utilizes these weights as a reference to complement the expression of similar cells.

### EAGS performs better smoothing by adaptive weighting

**Figure 2:**
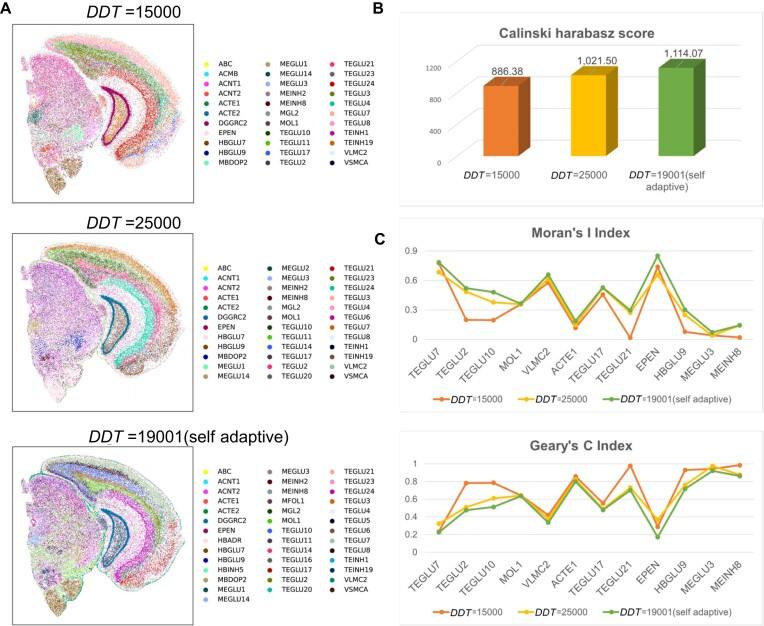
Results of EAGS with adaptive and fixed weights. (A) Spatial cell-type map for cell annotation with Spatial-ID using different weights for smoothing results. (B) The smoothing results with different weights are annotated with Spatial-ID cells. The Calinski–Harabasz Index is calculated using cell labels. (C) After the cell annotation using Spatial-ID with different weights, Geary's *C* and Moran's *I* are calculated from annotation results.

We use the mouse brain dataset to evaluate EAGS with adaptive weight. The results are compared to the outputs of EAGS with fixed weights. As the mouse brain dataset's adaptive weight value is 19,001, the fixed value weights are set to 25,000 and 15,000. We use Spatial-ID to annotate cell types in order to assess the potential of EAGS to improve the cell annotation power and restore the true levels of gene expression [24]. Fig. 2 shows all the results of the subsequent analysis with the adaptive and the fixed weights. The cell annotation results of EAGS using an adaptive weight compare to a fixed weight generated by a cell-type spatial map with clearer tissue outlines and more annotated cell-type subtypes (Fig. 2A).

Based on our cell annotation results, the CHI of the EAGS smoothing results with adaptive and fixed weights is calculated. Next, Geary's *C* and Moran's *I* of the common cell types in the annotation results are calculated (Fig. 2B, C). The results based on the adaptive weight cell annotation show a significant improvement in spatial autocorrelation compared to the others. Also, within the same type of cell annotation, the level of intraclass autocorrelation is higher.

### EAGS smooths gene expression with better performance on the simulated ST dataset

We collected a Bin140 specification mouse olfactory bulb ST dataset [27], with the top 2,000 highly variable genes selected as the reference input of ScDesign3 to construct a simulation space group with “ground-truth counts” [40]. To simulate the “dropout” phenomenon during the sequencing process, we randomly drop the simulated ST dataset expression to varying degrees and add different proportions of noise. EAGS, MAGIC [14], kNN-smoothing [41], SPCS [22], and STAGATE [20] are used to impute the processed ST dataset, and then L2-error with the “ground-truth counts” matrix and DBI are calculated, respectively. The results are shown in Table 1.

**Table 1:** Results on the simulated ST dataset with different proportions of dropout and noise

Dataset	Method	10% noise		20% noise		30% noise	
		L2-error	DBI	L2-error	DBI	L2-error	DBI
30% dropout	MAGIC	473.0679	4.7818	524.2593	4.4600	562.8816	4.2533
	kNN-smoothing	381.4241	4.4481	448.7622	4.2175	504.8468	3.9928
	SPCS	322.4659	4.6961	390.2348	4.3379	460.2699	4.2569
	STAGATE	757.3749	11.3146	790.5977	10.4536	791.5948	4.7064
	EAGS	**313.4211**	**4.1926**	**379.4374**	**3.9912**	**449.6919**	**3.9589**
50% dropout	MAGIC	496.2557	4.9018	557.0938	4.4717	590.9306	4.2881
	kNN-smoothing	389.2189	5.2899	458.8456	4.4637	517.1581	4.7092
	SPCS	319.4318	4.8357	398.8399	4.3223	475.0918	4.2884
	STAGATE	705.9759	8.7298	747.5357	76.9083	872.2147	9.3826
	EAGS	**310.2873**	**4.6808**	**386.7170**	**4.3201**	**459.1241**	**4.2065**
70% dropout	MAGIC	506.0679	6.3969	571.8875	5.9789	600.8655	5.7213
	kNN-smoothing	390.3494	7.7208	477.8630	6.6270	532.8365	6.1928
	SPCS	321.1818	**5.9028**	413.9192	5.7685	488.5445	5.6784
	STAGATE	850.6077	13.1108	814.2477	15.8843	693.4574	7.2281
	EAGS	**312.1247**	6.0768	**395.1362**	**5.6497**	**467.0692**	**5.6410**

The best values are set bold.

From Table 1, in the simulated datasets with 30% and 50% dropout, L2-error and DBI values obtained by EAGS are always the lowest, regardless of the proportion of noise. When the proportion of dropout is 70%, DBI obtained by EAGS is suboptimal with 10% noise (only higher than that of SPCS), and the results obtained by EAGS are the best on the other cases. In general, EAGS performs better on different simulated ST datasets and shows obvious advantages in improving intracell similarity and consistency with the “ground-truth counts” compared with other methods.

### EAGS smooths gene expressions for better characterizing the spatial expression patterns of mouse brain

**Figure 3:**
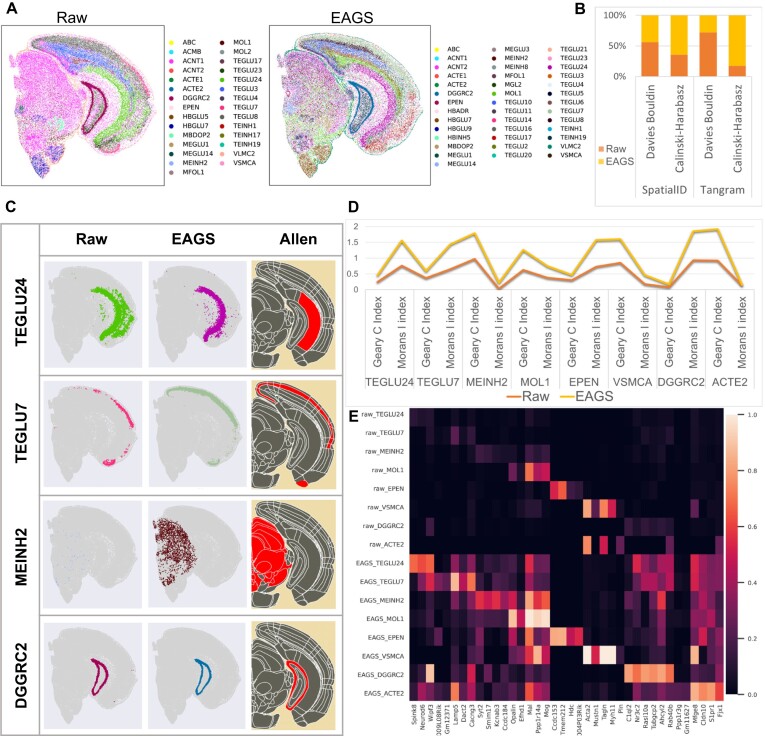
Comparisons between the analysis results obtained from data before and after EAGS smoothing. (A) Spatial cell-type maps of the mouse brain using Spatial-ID cell annotation of raw and EAGS smoothed data. (B) Davies–Bouldin and Calinski–Harabasz Indexes calculated using Spatial-ID and Tangram annotation results obtained from raw and EAGS smoothed data. (C) Comparison of the spatial map and Allen Mouse Brain Atlas obtained from the raw and EAGS smoothed dataset. (D) Comparison of Moran's *I* and Geary's *C* cell annotation types obtained from the raw and EAGS smoothed dataset. (E) Heatmap of nonzero ratio between the number of cell types and their marker genes obtained from the raw and EAGS smoothed dataset.

We perform cell annotation on mouse brain data before and after EAGS smoothing using Spatial-ID [24]. The annotation results are shown in Fig. 3A. The mouse brain cell annotation based on data smoothed by EAGS returns a clearer tissue structure, and more cell types can be annotated. To further assess the improvement provided by EAGS in cell annotation, we also perform cell annotation with Tangram [42], a technique for merging spatial data types with single-cell/single-nucleus RNA sequencing data and for cell-type annotation. As shown in Fig. 3B, the CHI and DBI are calculated for the spatial autocorrelation of cell types with the gene expression profiles after Tangram and Spatial-ID cell annotation. These results show that EAGS smoothing provides significantly better results in cell-type annotations.

Fig. 3C shows the results of cell annotation using Spatial-ID and the spatial map of the Allen Mouse Brain Atlas of corresponding cell types [25, 26]. TEGLU24, TEGLU7, and MEINH2 are important cell types in the hippocampus, cortex, and dorsal midbrain, respectively, and DGGRC2 is the important cell type in the ventral midbrain and dentate gyrus. These cell types are more consistent with Allen's spatial expression map of cell types after EAGS smoothing. To verify the smoothing effect, Moran's *I* and Geary's *C* are calculated for cells with different cell number ratios using the raw or the EAGS smoothed dataset (Fig. 3D). To determine whether the correlation between the above cell types and their marker genes improved after smoothing, the ratios of the number of annotated cell types to their corresponding nonzero marker gene expressions are computed. The ability of EAGS to restore true biological signals is shown in Fig. 3E. Our results show that EAGS contributes to enhancing the cellular features of the mouse brain as well as the spatial autocorrelation and intraclass similarity of the gene expression patterns.

### EAGS improves spatial patterns and downstream analyses of gene expression data

**Figure 4:**
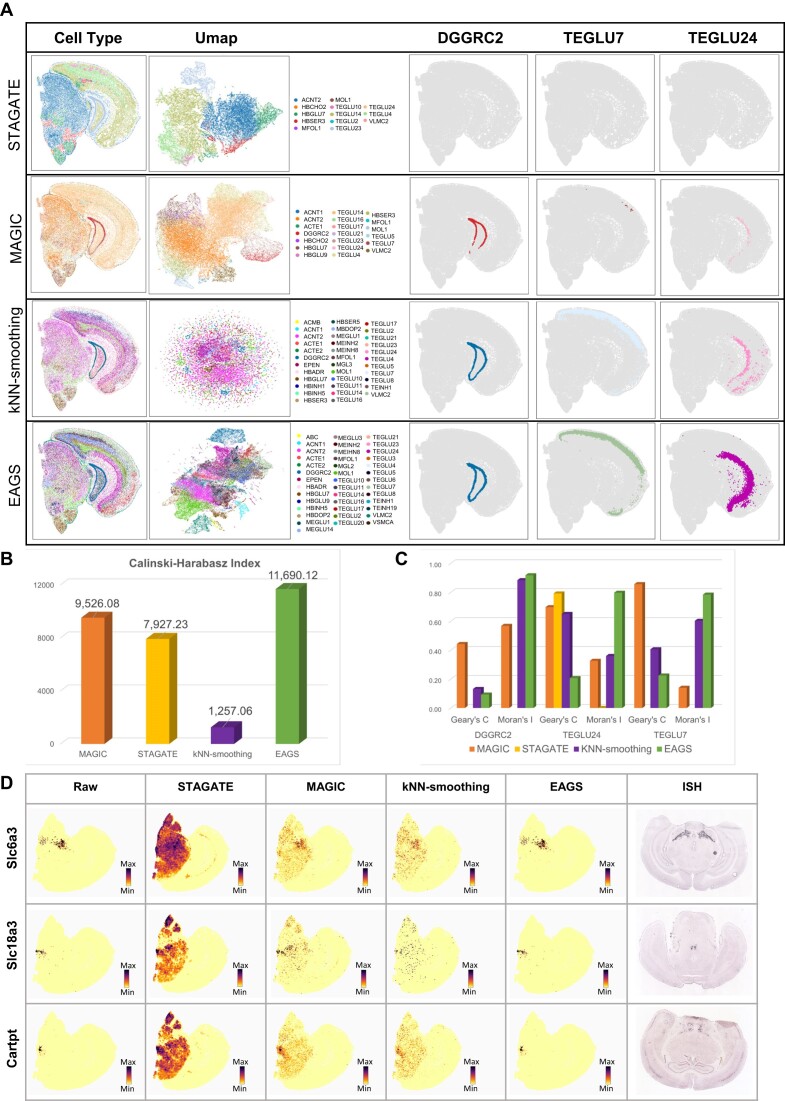
Comparison of different imputation methods. (A) Left: Spatial maps of cell types using Spatial-ID cell annotations and 4 different imputation methods. Middle: UMAP dimensionality reduction using Spatial-ID cell annotation and different imputation methods. Right: Individual cell-type spatial maps after cell annotation and different imputation methods. (B) Calinski–Harabasz Index calculated using cell labels after Spatial-ID cell annotations and different imputation methods. (C) Moran's *I* and Geary's *C* for the DGGRC2, TEGLU7, and TEGLU24 cell types. (D) Marker gene heatmaps and Mouse Brain Atlas obtained using different imputation methods.

EAGS is compared with the imputation methods, MAGIC [14], STAGATE [20], and kNN-smoothing [41], on the ST mouse brain dataset (SPCS cannot be executed successfully because the sparsity of this dataset is high, causing it to be out of memory, and thus the results SPCS are not obtained). The cell-type space map of different imputation methods using Spatial-ID as reference is shown in Fig. 4A (left). EAGS returns more cell types and more prominent outlines than other methods. The results of MAGIC are very unbalanced in terms of the number of cell types, with a large number of cell annotations that did not match the true values [25, 26]. The annotations of the dorsal midbrain, the ventral midbrain, and the dentate gyrus are mixed using MAGIC. The results of STAGATE show fewer cell types. Also, STAGATE does not result in well-organized cell-type distributions in the hippocampus and cortex. The cell-type boundaries of cell annotation after kNN-smoothing processing are blurred, and different types of cells are mixed. In order to avoid the impact of data sparsity on the interpretability of the results, the input data of the cell annotation are the 50th-dimensional principal component of different imputation results; the Uniform Manifold Approximation and Projection (UMAP) of the annotated results is shown in Fig. 4A (middle). The cell-type space maps, consisting of cell types that are highly represented and annotated by the 3 methods, are shown in Fig. 4A (right). Fig. 4B shows the CHI derived from data processed using 1 of the 4 methods. After cell annotation, CHI [35] calculated by the cell annotation label using EAGS shows higher spatial autocorrelation than the other 3 methods. EAGS obtains a higher Moran's *I* and Geary's *C* than the other methods (Fig. 4C). Additionally, the spatial maps of a few marker genes based on their expression are generated (Fig. 4D). The gene expression profiles smoothed by EAGS agree with Allen's ISH image better than the other methods.

To evaluate the efficiency of high-resolved ST data, we run EAGS, MAGIC, STAGATE, kNN-smoothing, Scimpute, and Drimpute 3 times on the ST mouse brain dataset and monitor the average runtime. For the sake of fairness, in this running time comparison, all methods use the CPU uniformly. EAGS requires the shortest run time, taking 3,484 seconds, while MAGIC takes 4,109 seconds, kNN-smoothing costs 4,739 seconds, and the other methods need a large memory consumption and cannot reach their final output in an acceptable time. The ST mouse brain dataset for STAGATE imputation is generated utilizing a GPU platform.

### EAGS application to a high-resolved ST dataset of other biological tissues

**Figure 5:**
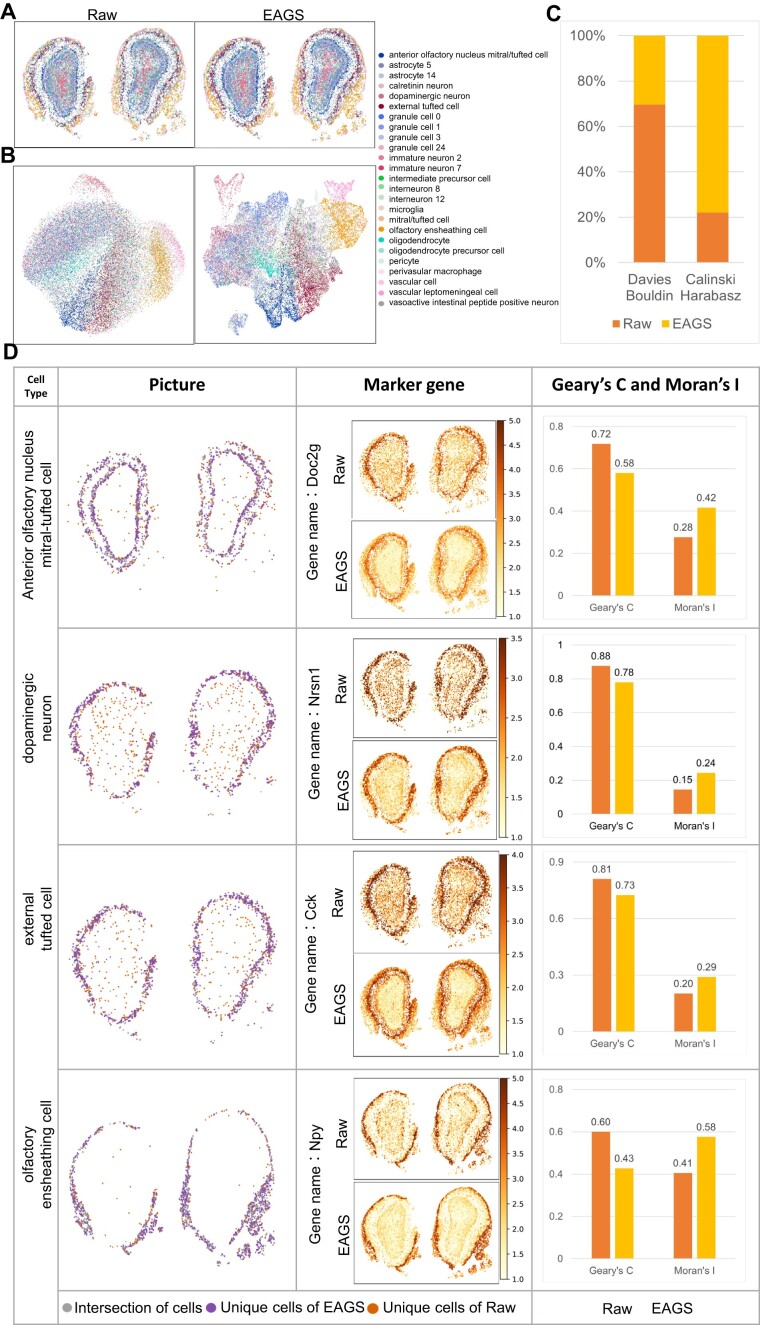
EAGS application to mouse olfactory bulb data. (A) Cell-annotated spatial map of data before and after EAGS smoothing. (B) Cell-annotated UMAP of the dataset before and after EAGS smoothing. (C) Davies–Bouldin and Calinski–Harabasz Indexes of mouse olfactory bulb data. (D) How the annotation results of the main cell types of the mouse olfactory bulb differ between data without and with EAGS smoothing. We also show the heatmap of the marker genes of different cell types and Moran's *I* and Geary's *C* indexes of the corresponding types. Cells annotated before and after smoothing (gray), cells annotated by EAGS alone (purple), and cells annotated by pretreatment data alone (orange) are displayed on the left side; the expression heatmap of marker genes corresponding to different cell types is shown in the middle; the Moran's *I* and Geary's *C* indices are shown on the right side.

To verify EAGS's adaptability to high-resolved ST data, we next apply EAGS to the mouse olfactory bulb dataset. We generate the mouse olfactory bulb spatial cell map with cell-type annotations (Fig. 5A) and the UMAP with cell annotation labels (Fig. 5B). The cell-annotated spatial map of the EAGS results shows a clearer outline of the cells in the mouse olfactory bulb (Fig. 5A). The results of EAGS in UMAP form easily distinguishable clusters in the transcriptome space, and the clusters of different cell types have a low degree of overlap (Fig. 5B). We then calculate the CHI and DBI of the results generated without and with EAGS. EAGS can generate the results with higher intraclass similarity. Also, cells belonging to the same annotation type are closer to each other when the data have been smoothed by EAGS (Fig. 5C). Next, we count the cell types with a high proportion of Tangram cell labels to generate a spatial cell map and make a heatmap of the expression of the corresponding marker genes for different types of cells (Fig. 5D). Then we classify the sources of different cell labeling results and calculate Geary's *C* and Moran's *I*. The cell-type annotation profile generated through the dataset smoothed by EAGS is clearer. Also, the corresponding marker gene expression is more concentrated, and the cell types have higher Geary's *C* and Moran's *I* if the data have been processed using EAGS. These results indicate a stronger spatial autocorrelation in the transcriptome space.

## Discussion

EAGS defines patterns based on expression and spatial information. Specifically, it selects similar elements from the intersection between cells of 2 patterns, ensuring a reliable source of information is borrowed between smoothed cells and similar cells. The main source of smoothing information for EAGS is the smoothing weights adaptively generated based on gene expression profiles. EAGS considers the overall expression level to generate weights, avoids the appearance of a single edge value, and effectively ensures the reliability of information borrowed between cells. This allows one to recover authentic cellular signals with improved intracellular similarity and spatial autocorrelation. For example, the expression of the Cartpt gene in Fig. 4D is scattered in the original data heatmap, with more noise appearing, and the matching degree with Allen's ISH image is low. EAGS smoothing considers the reliability of adjacent information. After EAGS smoothing, a lot of noise is eliminated, and more Cartpt genes are expressed in the correct cells, which is higher consistency with Allen's ISH image, and the aggregation of Cartpt expression is significantly improved. Furthermore, EAGS improves the quality of raw data as it recovers the original biological signals by smoothing cell expression information. The dimensional space is adjusted to ensure the hidden correlation between cells. As it does not depend on a specific statistical model, EAGS does not adjust from the low-dimensional space of the expression profile, thus ensuring the hidden correlation between cells. More important, EAGS does not require predefined expression models, numerous iterations to obtain the model parameters, or multiple training sessions on the deep learning model framework of the GPU platform. Consequently, EAGS significantly reduces computational costs and offers a significant execution advantage over other methods. Finally, because of the general applicability of smoothing, EAGS is suitable for different ST data.

It should be noted that the EAGS model is based on the premise that “neighboring” cells in the spatial microenvironment of biological tissues are more similar, which is applicable to most developmental tissue systems. However, for complex microenvironments with high biological heterogeneity (such as tumor microenvironment, etc.), this assumption will be challenged. EAGS may result in many false-positive signals. When it is necessary to perform EAGS on complex tumor microenvironment samples, when calculating the adaptive Gaussian smoothing weight, the sample may need to be partitioned according to different situations, and the Gaussian weight is calculated for different areas.

## Conclusions

We propose EAGS, a method for smoothing high-resolved ST datasets that performs 2-factor smoothing and adaptive weighting on raw gene expression profiles. EAGS significantly improves computing efficiency, reduces “dropout” in ST data, recovers the expression of true biological signals, and restores the spatial patterns of tissues. In the future, we will explore the false-positive signals produced by EAGS imputation strategies, as well as downstream analyses of datasets after imputation.

## Availability of Source Code and Requirements

Project name: EAGS: efficient and adaptive Gaussian smoothing

Project homepage: https://github.com/STOmics/EAGS

Operating system(s): Platform independent

Programming language: Python

Other requirements: Python 3.8 or higher

License: MIT License

RRID: SCR_024399

BiotoolsID: EAGS

## Data Availability

The mouse brain dataset at single-cell resolution is available in the STOmics DB of China National Gene Bank (CNGB) (accession code: “STT0000022”) [43, 44]. The mouse olfactory bulb at single-cell resolution is available in the STOMICS DataBase (accession code: “STT0000027”) [43, 44]. The mouse olfactory bulb data for the Bin140 specification are available in the China National Gene Bank (CNGB) (accession code: “CNP0001543”) [7]. The ST data at single-cell resolution with spatial information are available in Zenodo [45]. An archival copy of the code and supporting data is available via the *GigaScience* repository, GigaDB [46].

## Abbreviations

CHI: Calinski–Harabasz Index; DBI: Davies–Bouldin Index; DDT: distance distribution threshold; EAGS: efficient and adaptive Gaussian smoothing; ISH: *in situ* hybridization; MID: molecular identifier; scRNA-seq: single-cell RNA sequencing; ST: spatial transcriptomics; Stereo-seq: spatially enhanced resolution transcriptome sequencing; UMAP: Uniform Manifold Approximation and Projection.

## Competing Interests

The authors declare they have no competing interests.

## Funding

This work was supported by the National Key R&D Program of China (2022YFC3400400).

## Authors’ Contributions

X.X. and S.B.: project administration and supervision. T.L. and Y.Z.: algorithm development and implementation. T.L., M.L., and Q.K.: data collection, processing, and application. M.L., S.F., and Y.Z.: project coordination. T.L. and Q.K.: method comparisons, manuscript writing, and figure generation. T.L., M.L., Q.K., S.F., and S.B.: manuscript review.

## Supplementary Material

giad097_GIGA-D-23-00147_Original_Submission

giad097_GIGA-D-23-00147_Revision_1

giad097_GIGA-D-23-00147_Revision_2

giad097_Response_to_Reviewer_Comments_Original_Submission

giad097_Response_to_Reviewer_Comments_Revision_1

giad097_Reviewer_1_Report_Original_Submission Peijie Zhou -- 7/2/2023

giad097_Reviewer_1_Report_Revision_1 Peijie Zhou -- 9/15/2023

giad097_Reviewer_2_Report_Original_Submission Vaibhav Jain -- 8/1/2023
