# Supplementary material for: EAGS: efficient and adaptive Gaussian smoothing applied to high-resolved spatial transcriptomics
Source: Gigascience. 2024 Feb 20;13:giad097. doi: 10.1093/gigascience/giad097 (PMC10939424; doi:10.1093/gigascience/giad097)
Supplement: giad097_GIGA-D-23-00147_Original_Submission [file giad097_giga-d-23-00147_original_submission.pdf]

GigaScience

EAGS: efficient and adaptive Gaussian smoothing applied to high-resolved spatial transcriptomics

--Manuscript Draft--

Manuscript Number:	GIGA-D-23-00147
Full Title:	EAGS: efficient and adaptive Gaussian smoothing applied to high-resolved spatial transcriptomics
Article Type:	Technical Note
Funding Information:	
Abstract:	The emergence of high-resolved spatial transcriptomics (ST) has facilitated the research of novel methods to investigate biological development, organism growth, and other complex biological processes. However, high-resolution and whole transcriptomics ST datasets require customized imputation methods to improve the signal-to-noise ratio and the data quality. We propose an efficient and adaptive Gaussian smoothing (EAGS) imputation method for high-resolved ST. The adaptive two-factor smoothing of EAGS creates patterns based on the spatial and expression information of the cells, creates adaptive weights for the smoothing of cells in the same pattern, and then utilizes the weights to restore the gene expression profiles. We assessed the performance and efficiency of EAGS using high-resolved ST data of mouse brain and olfactory bulb. Compared with other competitive methods, EAGS shows higher clustering accuracy, better biological interpretations, and significantly reduced computational consumption.
Corresponding Author:	Xun Xu, Ph.D<br>BGI<br>Shenzhen, Guangdong CHINA
Corresponding Author Secondary Information:	
Corresponding Author's Institution:	BGI
Corresponding Author's Secondary Institution:	
First Author:	Xun Xu, Ph.D
First Author Secondary Information:	
Order of Authors:	Xun Xu, Ph.D
	Tongxuan Lv
	Ying Zhang, Ph.D
	Mei Li
	Qiang Kang, Ph.D
	Shuangsang Fang
	Yong Zhang
	Susanne Brix
Order of Authors Secondary Information:	
Additional Information:	
Question	Response
Are you submitting this manuscript to a special series or article collection?	No
**Experimental design and statistics**	Yes

Powered by Editorial Manager® and ProduXion Manager® from Aries Systems Corporation

Full details of the experimental design and statistical methods used should be given in the Methods section, as detailed in our Minimum Standards Reporting Checklist. Information essential to interpreting the data presented should be made available in the figure legends.<br><br>Have you included all the information requested in your manuscript?	
**Resources**<br><br>A description of all resources used, including antibodies, cell lines, animals and software tools, with enough information to allow them to be uniquely identified, should be included in the Methods section. Authors are strongly encouraged to cite Research Resource Identifiers (RRIDs) for antibodies, model organisms and tools, where possible.<br><br>Have you included the information requested as detailed in our Minimum Standards Reporting Checklist?	Yes
**Availability of data and materials**<br><br>All datasets and code on which the conclusions of the paper rely must be either included in your submission or deposited in publicly available repositories (where available and ethically appropriate), referencing such data using a unique identifier in the references and in the "Availability of Data and Materials" section of your manuscript.<br><br>Have you have met the above requirement as detailed in our Minimum Standards Reporting Checklist?	Yes

Powered by Editorial Manager® and ProduXion Manager® from Aries Systems Corporation

Manuscript
Click here to access/download;Manuscript;renamed_e0e34.docx

EAGS: efficient and adaptive Gaussian smoothing applied to high-resolved spatial transcriptomics

Tongxuan Lv[1,2,†], Ying Zhang[1,†], Mei Li[1,4,†], Qiang Kang[1,‡], Shuangsang Fang[1,3], Yong Zhang[1], Susanne Brix[4,*], Xun Xu[1,2,*]

[1] BGI-Shenzhen, Shenzhen 518103, China
[2] College of Life Sciences, University of Chinese Academy of Sciences, Beijing 100049, China
[3] BGI-Beijing, Beijing 100101, China
[4] Department of Biotechnology and Biomedicine, Technical University of Denmark, 2800 Kgs. Lyngby, Denmark
[*] Corresponding: sbrix@dtu.dk, xuxun@genomics.cn
[†] These authors contributed equally as the first authors.
[‡] Senior author.

Abstract:
The emergence of high-resolved spatial transcriptomics (ST) has facilitated the research of novel methods to investigate biological development, organism growth, and other complex biological processes. However, high-resolution and whole transcriptomics ST datasets require customized imputation methods to improve the signal-to-noise ratio and the data quality. We propose an efficient and adaptive Gaussian smoothing (EAGS) imputation method for high-resolved ST. The adaptive two-factor smoothing of EAGS creates patterns based on the spatial and expression information of the cells, creates adaptive weights for the smoothing of cells in the same pattern, and then utilizes the weights to restore the gene expression profiles. We assessed the performance and efficiency of EAGS using high-resolved ST data of mouse brain and olfactory bulb. Compared with other competitive methods, EAGS shows higher clustering accuracy, better biological interpretations, and significantly reduced computational consumption.

Keywords: spatial transcriptomics; imputation; gaussian smoothing; adaptive weight

Introduction

Recent advances in barcode-based spatial transcriptomics (ST) technology include 10X Visium [1], Slide-Seq [2,3], and high-definition spatial transcriptomics [4]. These advances made it feasible to provide expression profile information of entire genes, which is extremely important for comprehending biological functions and interaction networks [5,6]. High-resolved ST is an essential technical support for analyzing complex biological problems, as the function of complex biological tissues is closely related to the location of the transcriptional expression events within the tissue. However, cell localization and identification are limited by technical factors, such as the chip capture area, the sequencing depth, and the resolution. Spatially enhanced resolution transcriptome sequencing (Stereo-seq) [7] is a new ST technology based on DNA nanoballs. Stereo-seq provides the highest resolution (500 nm) among all currently available ST technologies. Such breakthrough in resolution allows researchers to perform genome-wide analyses of gene expression at the capture site (spot) with a single-cell or even sub-cellular resolution. Wang et al. [8] applied Stereo-seq to the 3D reconstruction of the ST of *Drosophila* embryos and larvae, providing a spatial- and temporal-resolved transcriptomic map of the whole organism across the developmental stages for *Drosophila* research. Liu et al. [9] reconstructed the developmental trajectory of zebrafish embryos during their development by analyzing Stereo-seq and scRNA-seq data from different time points.

Barcode-based high-resolution ST technology captures fewer genes at a single sequencing site (spot) than low-resolution ST technologies, such as 10X Visium [1], leading to high sparsity of the complete gene expression profile. In certain cell cycle phases, some cells do not express a set of genes whose expression thus appears to be null. In addition, amplification bias, cell cycle, library creation, and poor RNA capture rates cause some genes to be expressed but not captured by DNA nanoballs; such genes are called “dropout” [10]. Such biases adversely affect downstream analyses, such as clustering, cellular interaction analyses, and pseudo-temporal reconstructions [11–14], when the raw data is directly processed.

Various imputation methods have been proposed to solve the “dropout” in gene expression for scRNA-seq data [15]. These imputation methods can be broadly classified into 3 categories according to their principles. The first category smooths or diffuses the levels of gene expression in cells with comparable expression patterns to correct (typically) all values (zero and non-zero). For instance, MAGIC imputes the missing data in scRNA-seq datasets based on the Markov chains of adjacent domains and recovers gene expression of the characterized cells by data diffusion [16]; DrImpute finds similar cells by consensus clustering and pools their gene expression values to estimate the loss [17]. The second category models the gene expression profile with an existing probabilistic statistical model to simulate the distribution of genes. For instance, SAVER assumes that each gene in each cell follows a Poisson-Gamma distribution (a negative binomial distribution) and estimates prior parameters to recover the expression of the missing genes using Poisson LASSO regression methods [18]. Scimpute constructs a mixed Gamma-Normal distribution based on the gene expression profile and uses a non-negative least squares regression model, sc-transform (R package), to perform the imputation [19]. The third category uses deep learning principles to capture the potential spatial representation of cells and reconstruct the expression matrix. DCA is an auto-encoder that predicts the parameters of the selected distribution to generate estimates [20]. These methods offer practical recommendations for single-cell imputation; however, these methods do not account for spatial information in ST data, and the methods based on specialized statistical models cannot be applied to the high sparsity of high-resolved ST data.

In recent years, ST-based imputation methods have been presented. Sprod first projects gene expression onto a potential space, connects nearest neighbor cells to construct patterns, and then learns the denoising matrix using a shared minimization of the graph’s Laplacian smoothing term and reconstruction errors [21]. For ST data without pathology images, Sprod provides cluster-based pseudo-images, but it does not accurately reflect the actual cell clustering situation. STAGATE introduces a graph attention auto-encoder to construct a spatial neighbor network based on sequencing spots. Next, it introduces a distribution of the spatial neighbor network in the middle layer of the self-encoder to learn the correlation of neighboring sequencing spots and subsequently obtains the recovered gene expression profile by decoder [22]. However, the labels processed based on a specific clustering method are not completely consistent with the reality of the biological organization. It has been noted that the self-attention layer of the network does not consider the interaction between spot pairs and the information about the graphical structure of the spots [23].

To address these problems, we propose an efficient and adaptive Gaussian smoothing (EAGS) method, which we applied to high-resolved ST. EAGS is based on the fact that the spatial location of cells in biological tissues has a close relationship with their microenvironment, and the gene expression levels of cells within the same microenvironment are similar [19,24]. EAGS constructs different patterns based on cell expression profiles and cell location information to generate a similarity matrix. The similarity matrix then assesses cellular similarity within expression profiles to recover true biosignatures. By refining the information from proximal cells using adaptive smoothing weights and generating new gene expression profiles, the “dropout” is reduced. The resulting dataset provides RNA abundances more accurately than the original gene expression profile and preserves more of the true biological signal. EAGS enables the usage of high-sparsity ST data since it is independent of prior statistical models of the expression preconditioning the gene expression profiles. More crucially, EAGS could be used for large-scale ST data without requiring a lot of operating memory since it does not call for the computation of parameters for a pre-defined model, skipping most of the iterative process. We applied EAGS to the most updated ST dataset and compared it with widely used imputation methods to evaluate its efficacy in terms of fewer “zeros” in the gene expression profiles, improved cell annotation, and spatial organization replication.

Methods

The workflow of EAGS

In EAGS, the original expression matrix with the single-cell resolution was first used to generate patterns based on expression and spatial information. Then, the tight relationship between cells was established using two distinct patterns. Finally, the smoothing weights calculated from the patterns were used to define the level of smoothing for each cell and were then applied to recalculate the gene expression.

Datasets

The data generated by Stereo-seq consists of two parts: one is the optical image map of the tissue sections, and the other is the ST data based on the *in situ* capture. The spatial location information of various cells and their associated single-cell gene expression profiles were acquired by first conducting cell identification and segmentation on the optical image, and then comparing image after cell segmentation with the sequencing spots with spatial coordinates [7,25]. We used the published mouse brain data [26] and mouse olfactory bulb data [25]. The mouse brain data includes 61,857 cells, and the mouse olfactory bulb data includes 33,272 cells. The In Situ Hybridization (ISH) images of the signature genes from the mouse brain were obtained to help compare the impacts of smoothing [27,28].

The gene expression profile with spatial information was pre-processed with the Scanpy toolbox (V1.9.1; RRID:SCR_018139) to remove low-quality signals that might be blended into the gene expression data [29,30]. First, we filtered genes based on expression in at least 10 cells: those genes were kept. Next, cell outliers were filtered using gene expression: cells expressing at least 300 MID counts were kept. The 2% highest MID counts in all cells were subtracted from the overall number of MIDs across all cells in the gene expression profile. Finally, the coordinates of the spatial position information of the cells and the log-transformed and normalized gene expression profiles were employed as input to EAGS.

Pattern construction

Since "similar cells" in organisms with comparable molecular microenvironments express their genes similarly, the regions with identical expression patterns may originate from the same cell type or from the same biological tissue location [19,24]. Using "similar cells" to supplement the information of a particular spot is feasible. Based on spatial location data and gene expression profiles, we constructed two patterns to divide the cells on an ST slice's gene expression profile into several clusters. A comprehensive description of these two pattern styles is given below:

Definition 1 (Gene Expression Pattern): If $\boldsymbol{P}_e(i)$ is the gene expression domain of $\boldsymbol{Cell}_i$ for ST data, then:

$$\forall \boldsymbol{Cell}_j \in \boldsymbol{P}_e(i),\ \forall \boldsymbol{Cell}_k \in \boldsymbol{P}_g - \left(\boldsymbol{P}_e(i) \cup \{\boldsymbol{Cell}_i\}\right),\ s.t. d_{ij}{}^e < d_{ik}{}^e \quad (1)$$

where $\boldsymbol{Cell}_i$, $\boldsymbol{Cell}_j$ and $\boldsymbol{Cell}_k$ are different cells, $\boldsymbol{P}_g$ is the global pattern of gene expression, $d_{ij}{}^e$ and $d_{ik}{}^e$ are the distance between $\boldsymbol{Cell}_i$ and $\boldsymbol{Cell}_j$, and $\boldsymbol{Cell}_i$ and $\boldsymbol{Cell}_k$, respectively.

Balltree is a binary tree data structure that performs well on high-dimensional datasets, especially for Fast Nearest-neighbor Search on high-dimensional datasets [31,32]. The complete gene expression profile is separated into many different subspaces by Balltree. Then, the Euclidean distances between cells are calculated separately. Assuming the pre-normalized gene expression profile still contains *m* cells, the unsupervised nearest neighbor network toolkit (scikit-learn) is used to extract the *n*-dimensional principal component data and creates the low-dimensional information matrix ($\boldsymbol{LDIM}_{(m,n)}$) for the gene expression profile, as shown in *Algorithm 1* [33]. Then, the neighboring cell matrix is constructed based on the K-Nearest Neighbors network as in *Algorithm 2,* forming the Expression Neighbor Matrix ($\boldsymbol{ENM}_{(m,m)}$). Different definitions are given depending on whether $\boldsymbol{Cell}_j$ can be attributed to the gene expression pattern of $\boldsymbol{Cell}_i$:

$$ENM_{(i,j)} = \begin{cases} 1,\ j = i \\ 1, \boldsymbol{Cell}_j \in \boldsymbol{P}_e(i) \\ 0, \boldsymbol{Cell}_j \notin \boldsymbol{P}_e(i) \end{cases} \quad (2)$$

where $ENM_{(i,j)}$ defines whether $\boldsymbol{Cell}_j$ is within the gene expression pattern $\boldsymbol{P}_e(i)$ of $\boldsymbol{Cell}_i$, if $ENM_{(i,j)}$ = 1, $\boldsymbol{Cell}_j$ belongs to the expression pattern of $\boldsymbol{Cell}_i$; if $ENM_{(i,j)}$ = 0, it does not.

Algorithm 1. Builds the tree structure of Balltree

Balltree is built using a divide-and-conquer method. Initially, Balltree has only one (root) node and all data points are assigned to it. At each step, the partition corresponding to each node is split into two sub-partitions. For a partition p_i, the splitting procedure is as follows:

Step 1: Find the centroid of the node points in $\boldsymbol{LDIM}_{(m,n)}$. Reducing an n-dimensional matrix to a two-dimensional plane, the centroid of the node is centroid 1.

Step 2: Select the farthest point from centroid 1 in p_i as the first (left) child pivot p_i^L.

Step 3: Select the farthest point from p_i^L as the second (right) child pivot p_i^R.

Step 4: Assign each data point p_i to the partition whose pivot is closer.

Step 5: Assign the new sub-partitions as children of v_i in Balltree, i.e., v_i^R and v_i^L.

Algorithm 2. Using Balltree to find the nearest Neighbor of each cell

Input: Balltree structure $nbrs$, nearest neighbor num k, test point t, Current node n

Output: Expression Neighbor Matrix ($\boldsymbol{ENM}$)

Algorithm: $ball-tree-research(nbrs,k,t,n)$

if $distance(t, node.pivot)$ - $node.radius$ $\geq max(q)$:

    return;

if node in leaf-node set:

    Add $node.pivot$ to Q refresh q

    If $length(Q)$ > k :

        Remove the point furthest from the test point

        Refresh q

else :

    $ball-tree-research(nbrs,k,t,node.son1)$

    $ball-tree-research(nbrs,k,t,node.son2)$

end if

return $\boldsymbol{ENM}$

The difference between ST and scRNA-seq data is that ST provides the spatial coordinate position of each sequencing site (spot). After StereoCell processing, ST data are spots with a single-cell resolution where every spot corresponds to a single physical cell with spatial coordinates [25]. Cells in adjacent regions of histological sections are more likely to come from the identical microenvironment and belong to similar or identical cell types than cells from other areas. Therefore, we offer the spatial neighborhood pattern as a reference and classify the cluster of cells that are physically adjacent to a specific cell as its "spatial neighborhoods":

Definition 2 (Spatial Neighbor Pattern): If $\boldsymbol{P}_s(i)$ is the spatial neighbor pattern of $\boldsymbol{Cell}_i$ for ST data, then:

$$\forall \boldsymbol{Cell}_j \in \boldsymbol{P}_s(i),\ s.t. d_{ij}^{\,s} \leq \tau_s \quad (3)$$

where $d_{ij}^{\,S}$ is the spatial distance between $\boldsymbol{Cell}_i$ and $\boldsymbol{Cell}_j$, and τ_s represents the maximum spatial distance of $\boldsymbol{P}_s(i)$ of $\boldsymbol{Cell}_i$.

Since the spatial distribution of ST data is a two-dimensional plane space, the Euclidean distance can serve as a useful measure of spatial location between cells in a low-dimensional environment. Therefore, the spatial distance Matrix ($\boldsymbol{SDM}_{(m,m)}$) is constructed by computing the Euclidean distance. Furthermore, since ST chips of the Stereo-seq platform vary in size, EAGS fine-

tunes the weight value for different chip sizes while calculating Euclidean distances.

Adaptive weight calculation

Cells can be used as smoothing factors for $\boldsymbol{Cell}_i$, and must satisfy both the gene expression pattern and the spatial neighbor pattern belonging to $\boldsymbol{Cell}_i$. A cell acting as the smoothing factor is more similar in gene expression to the smoothed cell than to other cells in the overall expression profile. EAGS defines the nearest neighbor contribution matrix ($\boldsymbol{NCM}_{(m,m)}$) for an ST dataset containing m cells as follows:

$$\boldsymbol{NCM}_{(m,m)} = \boldsymbol{SDM}_{(m,m)} \times \boldsymbol{ENM}_{(m,m)} \tag{4}$$

where the non-zero value $\boldsymbol{NCM}_{nonzero}$ part of the $\boldsymbol{NCM}_{(m,m)}$ is selected as the parameter for smoothing weights, and $\boldsymbol{NCM}_{nonzero}$ is a G-dimensional row vector, where the condition $G \le M \times M$ is satisfied. The pth percentile of the $\boldsymbol{NCM}_{nonzero}$ along the specified axis is calculated by the following method:

$$(G-1) \times p^{th} = c + t \tag{5}$$

where G represents the number of vectors of $\boldsymbol{NCM}_{nonzero}$; c and t represent the integer and fractional parts of the calculation result, respectively. The Distance Distribution Threshold (DDT) is defined as follows:

$$DDT = (1-t) \times \boldsymbol{NCM}_{nonzero}[c] + t \times \boldsymbol{NCM}_{nonzero}[c+1] \tag{6}$$

where the calculated c and t obtain the pth percentile DDT along the specified axis of the $\boldsymbol{NCM}_{nonzero}$. The calculation of the adaptive weights is based on the $\boldsymbol{NCM}_{nonzero}$:

$$\boldsymbol{GS}_{new} = \mathrm{GS}(\boldsymbol{NCM}) = a \times e^{-\frac{(\boldsymbol{NCM}-b)^2}{2\times\mu^2}} \tag{7}$$

where $\boldsymbol{GS}_{new}$ is the degree of smoothing information and is an adaptive weight determined by the degree of similarity between the cells in the pattern's framework, and $\mathrm{GS}()$ is used to calculate the adaptive weights. The precise smoothing weight contribution between cells is calculated as follows:

$$\mu = \sqrt{-\frac{(DDT-b)^2}{2 \times \ln\left(\frac{gs}{a}\right)}} \tag{8}$$

where gs is a hyperparameter that characterizes the overall smoothness of the reference gene expression profile, which represents the overall smoothness of the entire chip. For a 1×1 cm ST chip of the Stereo-seq platform, gs is set to 0.95. μ is the smooth weight that varies around the gs, and characterizes the overall contribution level of cells in both the $\boldsymbol{P}_e(i)$ and $\boldsymbol{P}_s(i)$ to $\boldsymbol{Cell}_i$.

Smooth

The raw gene expression profile can be processed after $\boldsymbol{GS}_{new}$ and raw expression $\boldsymbol{E}_{origin}$ have been obtained:

$$\boldsymbol{E}_{GS}(x) = \frac{\sum_{i \in P_A(i)} \boldsymbol{GS}_{new}(R(i,x)) \times \boldsymbol{E}_{origin}(i) + \boldsymbol{E}_x}{\sum_{i \in P_A(i)} \boldsymbol{GS}_{new}(R(i,x)) + 1} \tag{9}$$

where E_{GS} represents the level of gene expression after adaptive weight smoothing, $P_A(i)$

represents all cells in the region where cell x is smoothed, $\boldsymbol{E}_x$ represents the original gene expression of the smoothed cell. The whole process can be represented by *Algorithm 3*.

Algorithm 3. Calculate weights and perform smoothing

Input: Expression Neighbor Matrix($\boldsymbol{ENM}$), spatial distance matrix $\boldsymbol{SDM}_{(m,m)}$, Origin expression matrix $\boldsymbol{E}_{origin}$, gs

Output: Smooth expression Matrix $\boldsymbol{E}_{(GS)}$

Step 1：Calculating the K-nearest-neighbor cell Euclidean distance distribution.

Step 2：Smooth threshold takes the percentile value x of the distance distribution and requires a value from 0.2 to 1.

Step 3：Using Eq. (6) to back-calculate the magnitude of μ at this time; preset gs = 0.95.

Step 4：The Gaussian weights at other distances are calculated by substituting μ values into Eq. (7).

Step 5：Re-weighted summation based on the newly calculated Gaussian weights and the original expressions.

If relying entirely on the cells in the $P_A(i)$ as smoothing factors without using the origin gene expression of the smoothed $\boldsymbol{Cell}_i$, Eq. (10) can be further streamlined as:

$$\boldsymbol{E}_{GS}(x) = \frac{\sum_{i \in P_A(i)} \boldsymbol{GS}_{new}\left(R(i,x)\right) \times \boldsymbol{E}_{origin}(i)}{\sum_{i \in P_A(i)} \boldsymbol{GS}_{new}\left(R(i,x)\right)} \tag{10}$$

where $\boldsymbol{E}_{GS}$ is completely calculated from the expression level of cells in $P_A(i)$, regardless of the gene expression of $\boldsymbol{Cell}_i$.

Evaluation method

We evaluated the significance of the differences in intra-class and extra-class similarity of our clustering results using the Calinski-Harabasz index (CHI) and the Davies-Bouldin index (DBI). We used Moran’s I and Geary’s C to calculate the correlation of cellular marker genes in the gene expression space of the data before and after EAGS smoothing [34].

Calinski-Harabasz Index

The Calinski-Harabasz index computes the sum of squares of the distances between points in the class and the class center to determine how closely a class is related [35]. It is defined as:

$$CHI(k) = \frac{\mathrm{tr}\left(\boldsymbol{B}_q\right)}{\mathrm{tr}\left(\boldsymbol{W}_q\right)} \times \left(\frac{h-q}{q-1}\right) \tag{11}$$

where h is the number of training samples, q is the number of categories, $\boldsymbol{B}_q$ is the between-category covariance matrix, $\boldsymbol{W}_q$ is the within-category data covariance matrix, and $\mathrm{tr}()$ is the trace calculation function.

Davies-Bouldin Index

The Davies-Bouldin Index finds the maximum by calculating the quotient of the sum of the average intra-class distances of any two classes within the sample set and the distance between the centers of the two clusters [36]. It is defined as:

$$DB = \frac{1}{n}\sum_{i=1}^{n}\max_{i \neq j}\left(\frac{\sigma_i + \sigma_j}{d\left(\boldsymbol{c}_i, \boldsymbol{c}_j\right)}\right) \quad (12)$$

where n is the number of categories, $\boldsymbol{c}_i$ is the center of the ith category, σ_i is the average distance from all points of the ith category to the center, $d\left(\boldsymbol{c}_i, \boldsymbol{c}_j\right)$ is the distance between the center points $\boldsymbol{c}_i$ and $\boldsymbol{c}_j$, and $\max()$ is the maximum function.

Moran's I

Moran's I is a global autocorrelation statistic for certain metrics on a graph. It is commonly used in spatial data analysis to evaluate autocorrelation on two-dimensional grids [37]. It is defined as:

$$Index_{Moran's} = \left(\frac{N}{W}\right) \times \frac{\sum_{i=1}^{N}\sum_{j=1}^{N}\left(w_{ij} \times \left(x_i - \bar{x}\right) \times \left(x_j - \bar{x}\right)\right)}{\sum_{i=1}^{N}\left(x_i - \bar{x}\right)^2} \quad (13)$$

where N is the number of spatial units indexed by i and j, x is the variable of interest, $\bar{x}$ is the mean of x, w_{ij} are the elements of a matrix of spatial weights with zeros on the diagonal, and W is the sum of all w_{ij}.

Geary's C

Geary's C is a measure of spatial autocorrelation that attempts to determine if observations of the same variable are spatially autocorrelated globally (rather than at the neighborhood level) [38]. It is defined as:

$$C = \frac{\left(N-1\right) \times \sum_i \sum_j \left(\boldsymbol{w}_{ij} \times \left(x_i - x_j\right)\right)}{2 \times S_0 \times \sum_i \left(x_i - \bar{x}\right)^2} \quad (14)$$

where $\boldsymbol{w}_{ij}$ is the ith row of the spatial weight matrix with zeros on the diagonal, and S_0 is the sum of all the weights.

Results

Overview of EAGS

We have downloaded the data of mouse olfactory bulb and mouse brain as inputs to EAGS [25,26]. The acquisition process of these data is: stereo-seq [7] was used to capture the ST data of the mouse brain and mouse olfactory bulb *in situ* and record the position information of the sequencing spot, just like the data generation process in "Datasets" subsection, and then StereoCell [25] was used to generate ST data at single-cell resolution with spatial information. After obtaining the ST data at single-cell resolution, the entire gene expression profile was normalized and smoothed [39], as shown in Fig. 1A.

EAGS constructs two styles of patterns based on the input gene expression information and spatial information, respectively. These two patterns are used to identify similar cells within the pattern, as shown in Fig. 1B. Next, EAGS adaptively generates smoothing weights based on the difference between similar cells and their genes' expression, then utilizes these weights as a reference to complement the expression of similar cells.

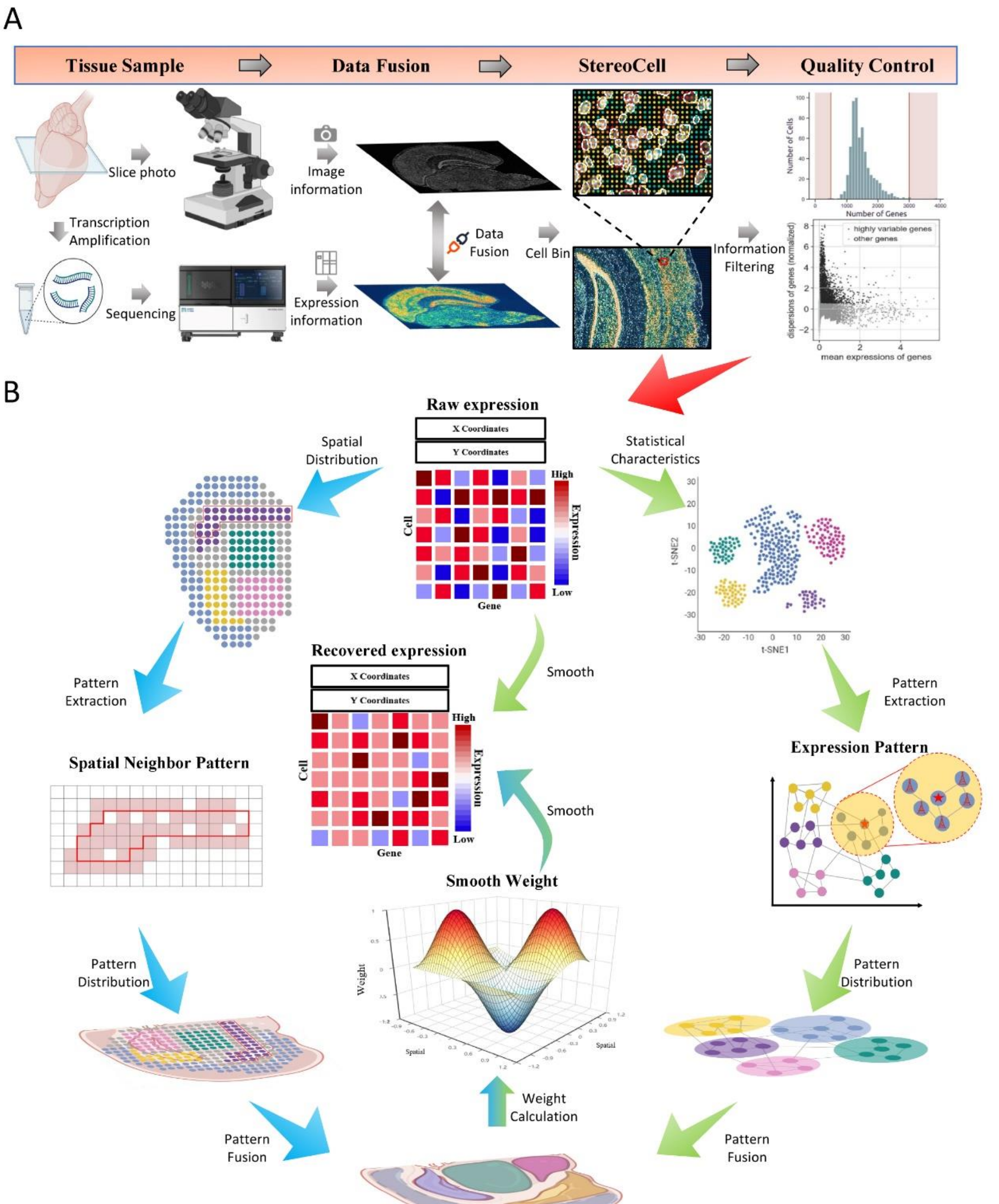

Figure 1: (A) Data generation process for the input of EAGS. (B) The EAGS method calculates the nearest neighbor information based on the gene expression pattern and spatial information. Then, EAGS adaptively generates smoothing weights and outputs the smoothed results.

EAGS performs better smoothing by adaptive weighting

We used mouse brain data to evaluate EAGS with adaptive weight. The results were compared to the output of EAGS with fixed weights. As the mouse brain data's adaptive weight value was 19,001, the fixed value weights were set to 25,000 and 15,000. We use Spatial-ID to annotate cell types in order to assess the potential of EAGS to improve the cell annotation power and restore the true levels of gene expression [26]. Fig. 2 shows all the results of the subsequent analysis with the adaptive and the fixed weights. The cell annotation results of EAGS using an adaptive weight compared to a fixed weight generated a cell-type spatial map with clearer tissue outlines and more annotated cell-type subtypes (Fig. 2A).

Based on our cell annotation results, the CHI of the EAGS smoothing results with adaptive and fixed weights were calculated. Next, Geary's C and Moran's I of the common cell types in the

annotation results were calculated (Fig. 2, B and C). The results based on the adaptive weight cell annotation showed a significant improvement in spatial autocorrelation compared to the others. Also, within the same type of cell annotation, the level of intra-class autocorrelation was higher.

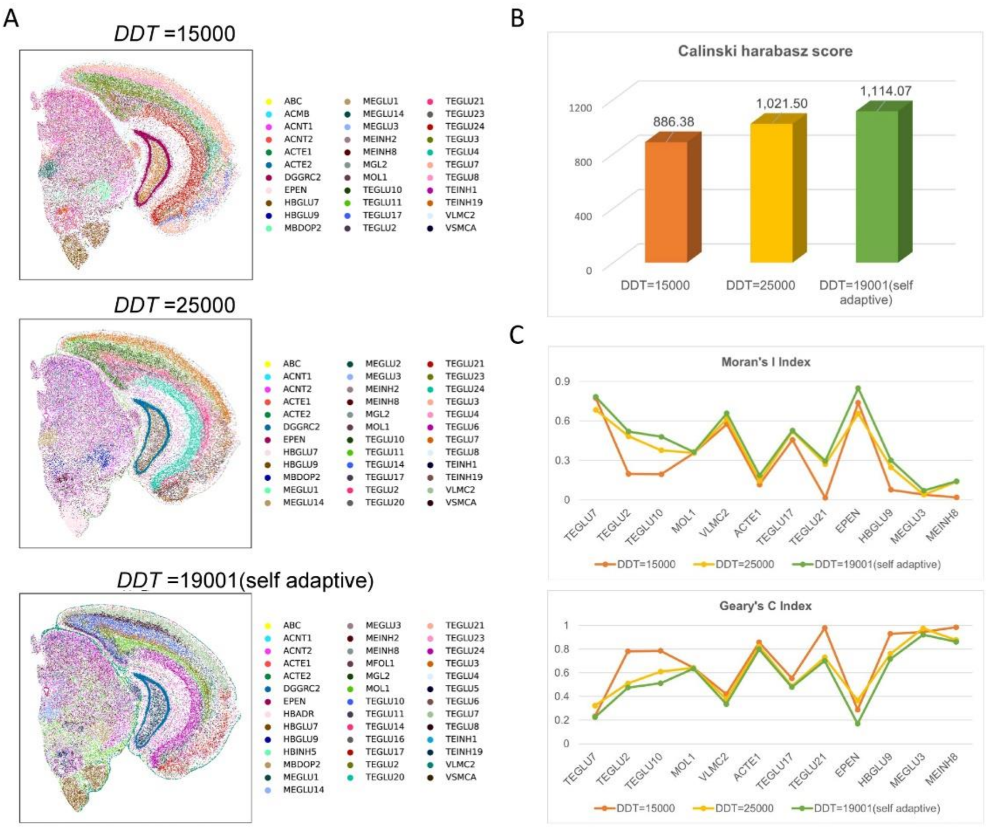

Figure 2: Results of EAGS with adaptive and fixed weights. (A) Spatial cell type map for cell annotation with Spatial-ID using different weights for smoothing results. (B) The smoothing results with different weights were annotated with Spatial-ID cells. The Calinski-Harabasz Index was calculated using cell labels. (C) After the cell annotation using Spatial-ID with different weights, Geary's C and Moran's I were calculated from the annotation results.

EAGS smooths gene expressions for better characterizing the spatial expression patterns of mouse brain

We performed cell annotation on mouse brain data before and after EAGS smoothing using Spatial-ID [26]. The annotation results are shown in Fig. 3A. The mouse brain cell annotation based on data smoothed by EAGS returned a clearer tissue structure, and more cell types could be annotated. To further assess the improvement provided by EAGS in cell annotation, we also performed cell annotation with Tangram [40], a technique for merging spatial data types with single cell/single nucleus RNA sequencing data and for cell type annotation. As shown in Fig. 3B, the CHI and DBI were calculated for the spatial autocorrelation of cell types with the gene expression profiles after Tangram and Spatial-ID cell annotation. These results show that EAGS smoothing provides significantly better results in cell-type annotations.

Fig. 3C shows the results of cell annotation using Spatial-ID and the spatial map of Allen Mouse Brain Atlas of corresponding cell types [27,28]. TEGLU24, TEGLU7 and MEINH2 are important cell types in the Hippocampus, Cortex and Midbrain dorsal respectively, and DGGRC2 is the important cell type in the Midbrain ventral and Dentate gyrus. These cell types were more consistent with the Allen's spatial expression map of cell types after EAGS smoothing. To verify the smoothing effect, Moran's I and Geary's C were calculated for cells with different cell number ratios using the raw or the EAGS smoothed data (Fig. 3D). To determine whether the correlation between the above cell types and their marker genes improved after smoothing, the ratios of the number of annotated cell types to their corresponding non-zero marker gene expressions were computed. The ability of EAGS to restore true biological signals is shown in Fig. 3E. Our results show that EAGS contributes to enhancing the cellular features of the mouse brain as well as the spatial autocorrelation and intraclass similarity of the gene expression patterns (Fig. 3).

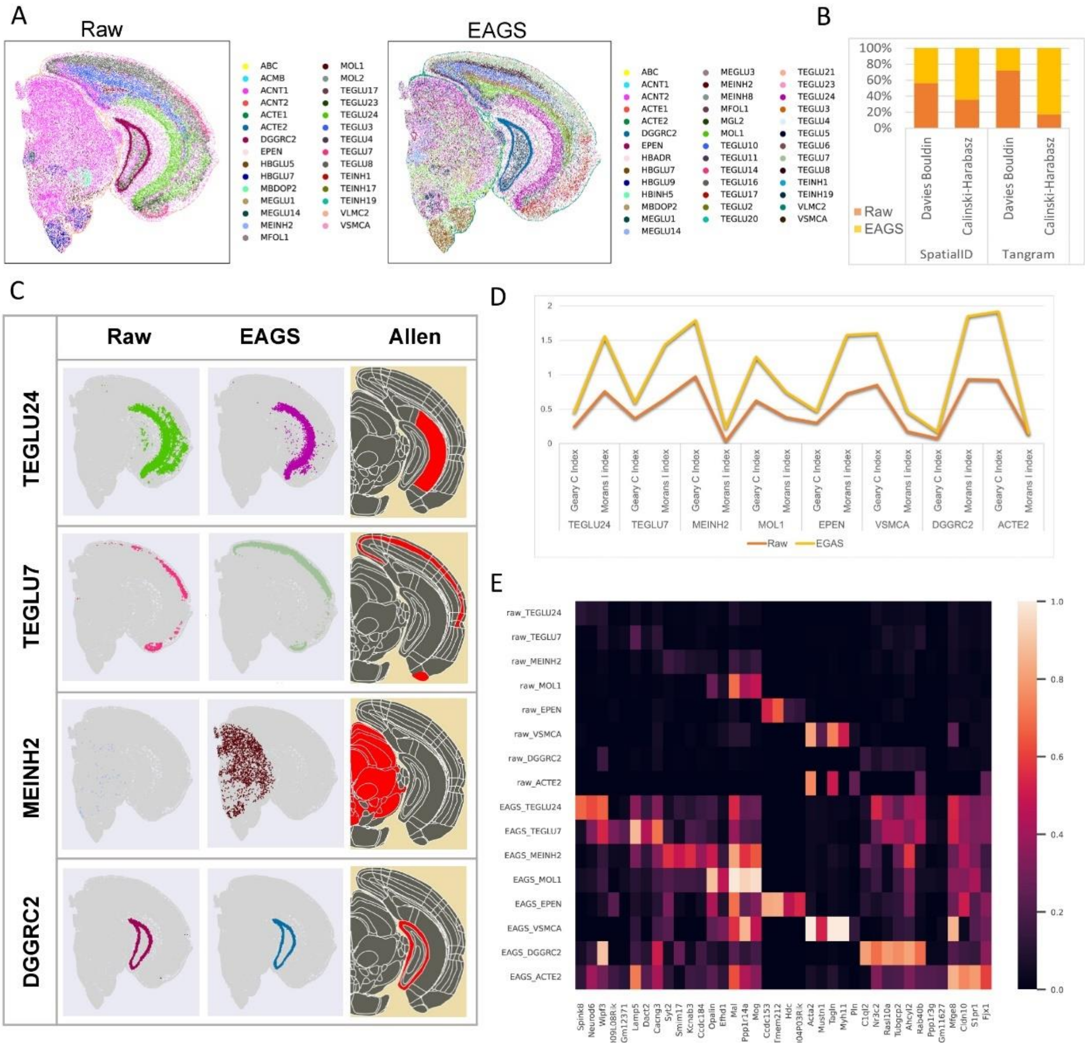

Figure 3: Comparisons between the analysis results obtained from data before and after EAGS smoothing. (A) Spatial cell type maps of the mouse brain using Spatial-ID cell annotation of raw and EAGS smoothed data. (B) Davies-Bouldin and Calinski-Harabasz Indexes calculated using Spatial-ID and Tangram annotation results obtained from raw and EAGS smoothed data. (C) Comparison of the spatial map and Allen Mouse Brain Atlas obtained from raw and EAGS smoothed data. (D) Comparison of Moran's I and Geary's C cell annotation types obtained from raw and EAGS smoothed data. (E) Heatmap of the non-zero ratio between the number of cell types and their marker genes obtained from raw and EAGS smoothed data.

EAGS improves spatial patterns and downstream analyses of gene expression data

EAGS was compared with two commonly used imputation methods: MAGIC [16] and STAGATE [22]. Fig. 4 compares the results of these methods on ST mouse brain data, and the results of different imputation methods using Spatial-ID are shown in Fig. 4A. EAGS returned more cell types and more prominent outlines than MAGIC or STAGATE. The results of MAGIC were very unbalanced in terms of the number of cell types, with a large number of cell annotations that did not match the true values [27,28]. The annotations of the Midbrain dorsal, the Midbrain ventral, and the Dentate gyrus were mixed using MAGIC. The results of STAGATE had fewer cell types. Also, STAGATE did not result in well-organized cell type distributions in the Hippocampus and Cortex. Fig. 4B shows the CHI derived from data processed using one of the three methods. After cell annotation, the CHI [35] calculated by the cell annotation label had higher spatial autocorrelation than MAGIC and STAGATE. In order to avoid the impact of data sparsity on the interpretability of the results, the input data of the cell annotation was the 50th-dimensional principal component of different imputation results; the Uniform Manifold Approximation and Projection (UMAP) of the annotated results is shown in Fig. 4C. The cell type space maps, consisting of cell types that were highly represented and annotated by the three methods, are shown in Fig. 4D. EAGS obtained higher Moran's I and Geary's C than MAGIC and STAGATE (Fig. 4E). Additionally, the spatial maps of a few marker genes based on their expression was generated (Fig. 4F). The gene

expression profiles smoothed by EAGS agreed with the Allen's ISH image better than the other methods.

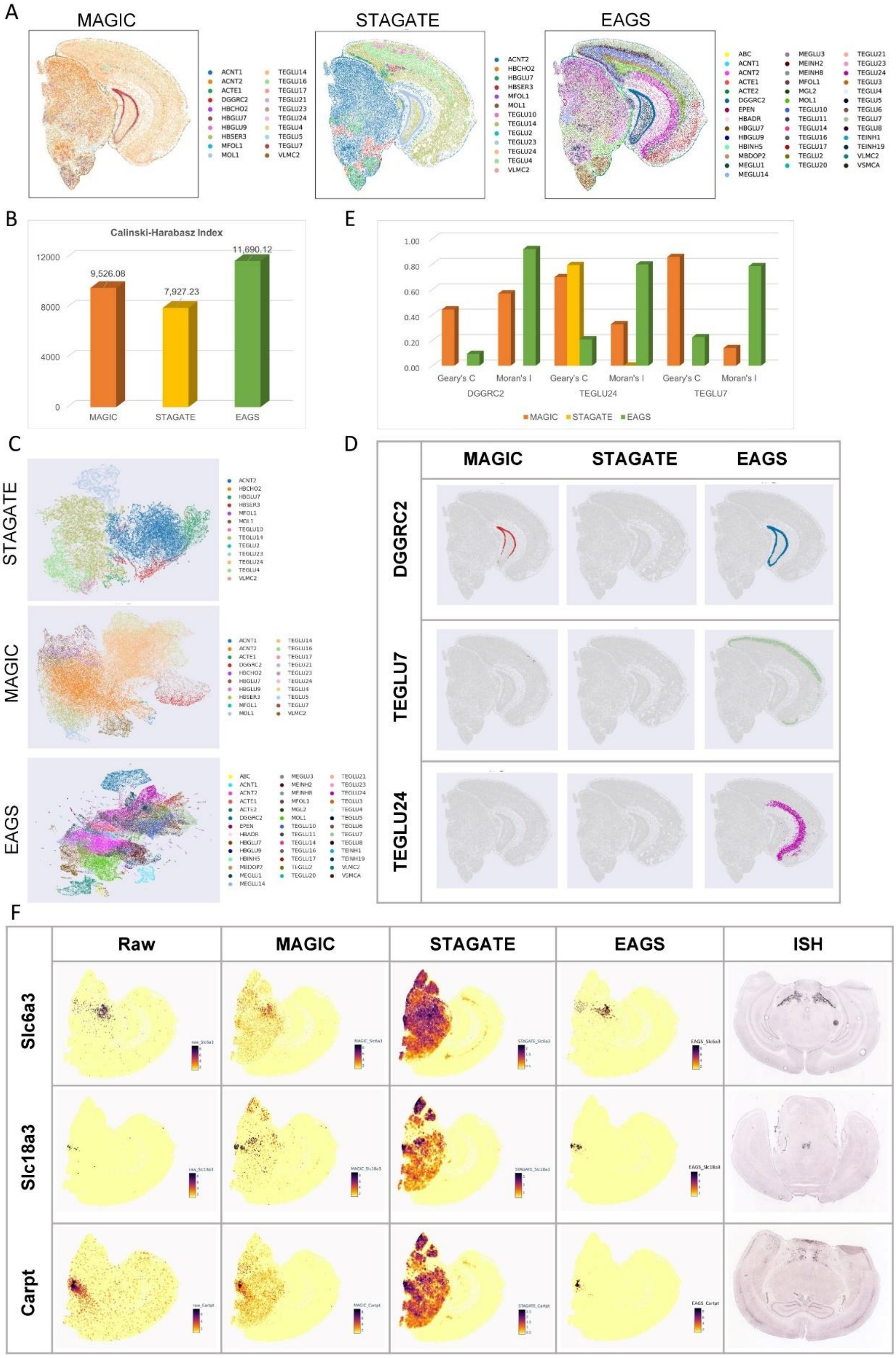

Figure 4: Comparison of different imputation methods. (A) Spatial maps of cell types using Spatial-ID cell annotations and three different imputation methods. (B) Calinski-Harabasz Index calculated using cell labels after Spatial-ID cell annotations and different imputation methods. (C) UMAP dimensionality reduction using Spatial-ID cell annotation and different imputation methods. (D) Individual cell type spatial maps after cell annotation and different imputation methods. (E) Moran's I and Geary's C for the DGGRC2, TEGLU7, TEGLU24 cell types. (F) Marker gene heatmaps and Allen graphs obtained using different imputation methods.

To evaluate the efficiency of high-resolved ST data, we ran EAGS, MAGIC, STAGATE, Scimpute, and Drimpute three times on mouse brain data and monitored the average run time. STAGATE was run using a GPU. For the sake of fairness, in this running time comparison, all methods used the CPU uniformly. EAGS required the shortest run time, taking 3,483 seconds, while MAGIC took 4,109 seconds, and the other methods needed a large memory consumption and could not reach their final output in an acceptable time. The mouse brain data for STAGATE imputation was generated utilizing a GPU platform.

EAGS application to high-resolved ST data of other biological tissues

To verify EAGS's adaptability to high-resolved ST data, we next applied it to olfactory bulb data (Fig. 5). We generated the mouse olfactory bulb spatial cell map with cell type annotations (Fig. 5A) and the UMAP with cell annotation labels (Fig. 5B). The cell-annotated spatial map of the EAGS results showed a clearer outline of the cells in the mouse olfactory bulb (Fig. 5A). The results of EAGS in UMAP formed easily distinguishable clusters in the transcriptome space, and the clusters of different cell types had a low degree of overlap (Fig. 5B). We then calculated the CHI and DBI of the results generated without and with EAGS. We found that EAGS generated results with higher intraclass similarity. Also, cells belonging to the same annotation type were closer to each other when the data had been smoothed by EAGS (Fig. 5C). Next, we counted the cell types with a high proportion of Tangram cell labels to generate a spatial cell map and make a heat map of the expression of the corresponding marker genes for different types of cells (Fig. 5D). Then we classified the sources of different cell labeling results and calculated Geary's C and Moran's I. The cell type annotation profile generated through data smoothed by EAGS was clearer. Also, the corresponding marker gene expression was more concentrated, and the cell types had higher Geary's C and Moran's I if the data had been processed using EAGS. These results indicate a stronger spatial autocorrelation in the transcriptome space.

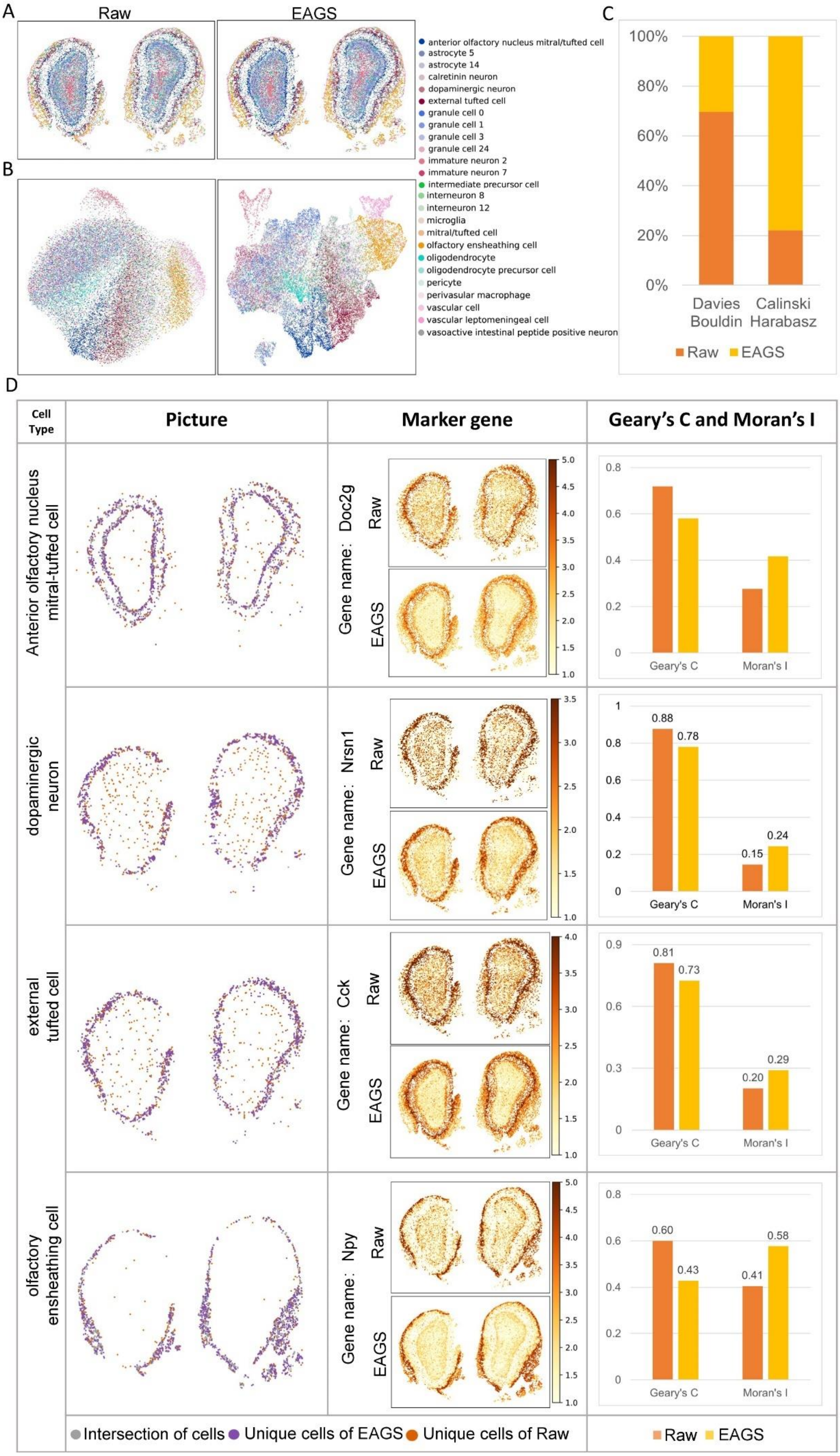
A
Raw
EAGS
anterior olfactory nucleus mitral/tufted cell
astrocyte 5
astrocyte 14
calretinin neuron
dopaminergic neuron
external tufted cell
granule cell 0
granule cell 1
granule cell 3
granule cell 24
immature neuron 2
immature neuron 7
intermediate precursor cell
interneuron 8
interneuron 12
microglia
mitral/tufted cell
olfactory ensheathing cell
oligodendrocyte
oligodendrocyte precursor cell
pericyte
perivasular macrophage
vascular cell
vascular leptomeningeal cell
vasoactive intestinal peptide positive neuron
B
C
100%
80%
60%
40%
20%
0%
Davies Bouldin
Calinski Harabasz
Raw
EAGS
D
Cell Type
Picture
Marker gene
Geary's C and Moran's I
Anterior olfactory nucleus mitral-tufted cell
Gene name: Doc2g
Raw
EAGS
dopaminergic neuron
Gene name: Nrsn1
0.88
0.78
0.15
0.24
external tufted cell
Gene name: Cck
0.81
0.73
0.20
0.29
olfactory ensheathing cell
Gene name: Npy
0.60
0.43
0.41
0.58
Geary's C
Moran's I
Intersection of cells
Unique cells of EAGS
Unique cells of Raw
Raw
EAGS

Figure 5: EAGS application to mouse olfactory bulb data. (A) Cell-annotated spatial map of data before and after EAGS smoothing. (B) Cell-annotated Umap of data before and after EAGS smoothing (C) Davies-Bouldin and Calinski-Harabasz Indexes of mouse olfactory bulb data. (D) How the annotation results of the main cell types of the mouse olfactory bulb differ between data without and with EAGS smoothing. We also show the heatmap of the marker genes of different cell types, and Moran's I and Geary's C indexes of the corresponding types. Cells annotated before and after smoothing (grey), cells annotated by EAGS alone (purple), and cells annotated by pre-treatment data alone (orange) are displayed on the left side; the expression heatmap of marker genes corresponding to different cell types are shown on the middle; the Moran's I and Geary's C indices are shown on the right side.

Discussions

EAGS defines patterns based on expression and spatial information. Specifically, it selects similar elements from the intersection between cells of two patterns, ensuring a reliable source of information is borrowed between smoothed cells and similar cells. The main source of smoothing information for EAGS is the smoothing weights adaptively generated based on gene expression profiles. EAGS considers the overall expression level to generate weights, avoids the appearance of a single edge value, and effectively ensures the reliability of information borrowed between cells. This allows to recover authentic cellular signals with improved intracellular similarity and spatial autocorrelation. Furthermore, EAGS improves the quality of raw data as it recovers the original biological signals by smoothing cell expression information. The dimensional space is adjusted to ensure the hidden correlation between cells. As it does not depend on a specific statistical model, EAGS does not adjust from the low-dimensional space of the expression profile, thus ensuring the hidden correlation between cells. More importantly, EAGS does not require pre-defined expression models, numerous iterations to obtain the model parameters, or multiple training sessions on the deep learning model framework of the GPU platform. Consequently, EAGS significantly reduces computational costs and offers a significant execution advantage over other methods. Finally, because of the general applicability of smoothing, EAGS is suitable for different ST data.

Conclusions

We propose EAGS, a method for smoothing high-resolved ST datasets that performs two-factor smoothing and adaptive weighting on raw gene expression profiles. EAGS significantly improves computing efficiency, reduces "dropout" in ST data, recovers the expression of true biological signals, and restores the spatial patterns of tissues. In the future, we will explore the false positive signals produced by EAGS imputation strategies, as well as downstream analyses of datasets after imputation.

Availability of Source Code and Requirements

Project name: EAGS: efficient and adaptive Gaussian smoothing
Project home page: https://github.com/BGIResearch/EAGS
Operating system(s): Platform independent
Programming language: Python
Other requirements: Python 3.8 or higher
License: MIT License

Data Availability

Mouse brain and mouse olfactory bulb data have been published in other studies, where the mouse brain data is available in China National Gene Bank (CNGB) (https://db.cngb.org/cnsa/, accession code: "CNP0002966"), and the mouse olfactory bulb is available in STOMICS DataBase (https://db.cngb.org/stomics/, accession code: "STT0000027"). The ST data at single-cell resolution with spatial information is available in Zenodo (https://doi.org/10.5281/zenodo.7906815).

Authors' Contributions

Project administration and supervision: Xun Xu, Susanne Brix
Algorithm development and implementation: Tongxuan Lv, Ying Zhang
Data collection, processing, and application: Tongxuan Lv, Mei Li, Qiang Kang
Project coordination: Mei Li, Shuangsang Fang, Yong Zhang
Method comparisons: Tongxuan Lv, Qiang Kang
Manuscript writing and figure generation: Tongxuan Lv, Qiang Kang
Manuscript review: Tongxuan Lv, Mei Li, Qiang Kang, Shuangsang Fang

Competing interests

The authors declare they have no competing interests.

Abbreviations

CHI: Calinski-Harabasz index; DBI: Davies-Bouldin index; DDT: Distance Distribution Threshold; EAGS: efficient and adaptive Gaussian smoothing; MIDs: molecular identifiers; ST: spatial transcriptomics; Stereo-seq: spatially enhanced resolution transcriptome sequencing. scRNA-seq: single-cell RNA seq; ISH: In Situ Hybridization; UMAP: uniform manifold approximation and projection.

Acknowledgments

We thank Guangdong Provincial Key Laboratory of Genome Read and Write (2017B030301011) for technical support for this study. We thank China National GeneBank for providing data support for this study.

Reference

1. Ji AL, Rubin AJ, Thrane K, et al. Multimodal Analysis of Composition and Spatial Architecture in Human Squamous Cell Carcinoma. Cell 2020;**182**(2):497-514. doi:10.1016/j.cell.2020.05.039.
2. Rodriques SG, Stickels RR, Goeva A, et al. Slide-seq: A scalable technology for measuring genome-wide expression at high spatial resolution. Science 2019;**363**(6434):1463-7. doi:10.1126/science.aaw1219.
3. Stickels RR, Murray E, Kumar P, et al. Highly sensitive spatial transcriptomics at near-cellular resolution with Slide-seqV2. Nat Biotechnol 2021;**39**:313-9. doi:10.1038/s41587-020-0739-1.
4. Vickovic S, Eraslan G, Salmén F, et al. High-definition spatial transcriptomics for in situ tissue profiling. Nat Methods 2019;**16**:987-90. doi:10.1038/s41592-019-0548-y.
5. Fang S, Chen B, Zhang Y, et al. Computational Approaches and Challenges in Spatial Transcriptomics. Genom Proteom Bioinf 2022. doi:10.1016/j.gpb.2022.10.001.
6. Longo SK, Guo MG, Ji AL, et al. Integrating single-cell and spatial transcriptomics to elucidate intercellular tissue dynamics. Nat Rev Genet 2021;**22**:627-44. doi:10.1038/s41576-021-00370-8.
7. Chen A, Liao S, Cheng M, et al. Spatiotemporal transcriptomic atlas of mouse organogenesis using DNA nanoball-patterned arrays. Cell 2022;**185**(10):1777-92. doi:10.1016/j.cell.2022.04.003.
8. Wang M, Hu Q, Lv T, et al. High-resolution 3D spatiotemporal transcriptomic maps of developing Drosophila embryos and larvae. Dev Cell 2022;**57**(10):1271-83.e4. doi: 10.1016/j.devcel.2022.04.006.

9. Liu C, Li R, Li Y, et al. Spatiotemporal mapping of gene expression landscapes and developmental trajectories during zebrafish embryogenesis. Dev Cell 2022;**57**(10):1284-98.e5. doi:10.1016/j.devcel.2022.04.009.
10. Kharchenko PV, Silberstein L, Scadden DT. Bayesian approach to single-cell differential expression analysis. Nat Methods 2014;**11**(7):740-2. doi:10.1038/nmeth.2967.
11. Ly LH, Vingron M. Effect of imputation on gene network reconstruction from single-cell RNA-seq data. Patterns (N Y) 2021;**3**(2):100414. doi:10.1016/j.patter.2021.100414.
12. Xu J, Cui L, Zhuang J, et al. Evaluating the performance of dropout imputation and clustering methods for single-cell RNA sequencing data. Comput Biol Med 2022;**146**:105697. doi:10.1016/j.compbiomed.2022.105697.
13. Hou W, Ji Z, Ji H, et al. A systematic evaluation of single-cell RNA-sequencing imputation methods. Genome Biol 2020;**21**:218. doi:10.1186/s13059-020-02132-x.
14. Dijk D, Sharma R, Nainys J, et al. Recovering Gene Interactions from Single-Cell Data Using Data Diffusion. Cell 2018;**174**(3):716-29.e27. doi:10.1016/j.cell.2018.05.061.
15. Gong W, Kwak IY, Pota P, et al. DrImpute: Imputing dropout events in single cell RNA sequencing data. BMC Bioinformatics 2018;**19**(1):220. doi:10.1186/s12859-018-2226-y.
16. Huang M, Wang J, Torre E, et al. SAVER: gene expression recovery for single-cell RNA sequencing. Nat Methods 2018;**15**(7):539-42. doi:10.1038/s41592-018-0033-z.
17. Li WV, Li JJ. An accurate and robust imputation method scImpute for single-cell RNA-seq data. Nat Commun 2018;**9**:997. doi:10.1038/s41467-018-03405-7.
18. Eraslan G, Simon LM, Mircea M, et al. Single-cell RNA-seq denoising using a deep count autoencoder Nat Commun. 2019;**10**:390. doi:10.1038/s41467-018-07931-2.
19. Wang Y, Song B, Wang S, et al. Sprod for de-noising spatially resolved transcriptomics data based on position and image information Nat Methods. 2022;**19**:950–8. doi:10.1038/s41592-022-01560-w.
20. Dong K, Zhang S. Deciphering spatial domains from spatially resolved transcriptomics with an adaptive graph attention auto-encoder. Nat Commun 2022;**13**:1739. doi:10.1038/s41467-022-29439-6.
21. Park W, Chang W, Lee D, et al. Graph Self-Attention for learning graph representation with Transformer. arXiv 2022;2201.12787. doi:10.48550/arXiv.2201.12787.
22. Liu Y, Wang T, Duggan B, et al. SPCS: a spatial and pattern combined smoothing method for spatial transcriptomic expression. Brief Bioinform 2022;**23**(3):bbac116. doi:10.1093/bib/bbac116.
23. Li M, Liu H, Li M, et al. StereoCell enables highly accurate single-cell segmentation for spatial transcriptomics. axXiv 2023;530414. doi:10.1101/2023.02.28.530414.
24. Shen R, Liu L, Wu Z, et al. Spatial-ID: a cell typing method for spatially resolved transcriptomics via transfer learning and spatial embedding. Nat Commun 2022;**13**:7640. doi:10.1038/s41467-022-35288-0.
25. Lein ES, Hawrylycz MJ, Ao N, et al. Genome-wide atlas of gene expression in the adult mouse brain. Nature 2007;**445**:168–76. doi:10.1038/nature05453.
26. Zeisel A, Hochgerner H, Lönnerberg P, et al. Molecular Architecture of the Mouse Nervous System. Cell. 2018;**174**(4):999-1014.e22. doi:10.1016/j.cell.2018.06.021.
27. Wolf FA, Angerer P, Theis FJ. SCANPY: Large-scale single-cell gene expression data analysis. Genome Biol 2018;**19**(1):15. doi:10.1186/s13059-017-1382-0.

28. Virshup I, Bredikhin D, Heumos L, et al. The scverse project provides a computational ecosystem for single-cell omics data analysis. Nat Biotechnol 2023;**41**:604–6. doi: 10.1038/s41587-023-01733-8.

29. Omohundro SM. Five balltree construction algorithms. International Computer Science Institute Technical Report; 1989.

30. Kumar N, Zhang L, Nayar S. What is a good nearest neighbors algorithm for finding similar patches in images? In European Conference on Computer Vision 2008;364–78. doi:10.1007/978-3-540-88688-4_27.

31. Pedregosa F, Varoquaux G, Gramfort A, et al. Scikit-learn: Machine learning in Python. J Mach Learn Res, 2011;**12**:2825-30.

32. Desgraupes B. Clustering Indices. Univ.Paris Ouest-Lab Modal'X 2013;**1**(34).

33. Caliñski T, Harabasz J. A Dendrite Method Foe Cluster Analysis. Commun Stat 1974;**3**:1,1-27. doi:10.1080/03610927408827101.

34. Hubert L, Arabic P. Comparing Partitions. J Classif 1985;**2**:193-218. doi:10.1007/BF01908075.

35. Moran PAP. Notes on Continuous Stochastic Phenomena. Biometrika 1950;**37**(1/2):17-23. doi:10.2307/2332142.

36. Geary RC. The Contiguity Ratio and Statistical Mapping. The Incorporated Statistician 1954;**5**(3):115–46. doi:10.2307/2986645.

37. Chen G, Ning B, Shi T. Single-Cell RNA-Seq Technologies and Related Computational Data Analysis. Front Genet 2019;**10**:317. doi:10.3389/fgene.2019.00317.

38. Biancalani T, Scalia G, Buffoni L, et al. Deep learning and alignment of spatially resolved single-cell transcriptomes with Tangram. Nat Methods 2021;**18**:1352–62. doi:10.1038/s41592-021-01264-7.

Figure

Click here to access/download;Figure;Figure.pdf

Fig. 1

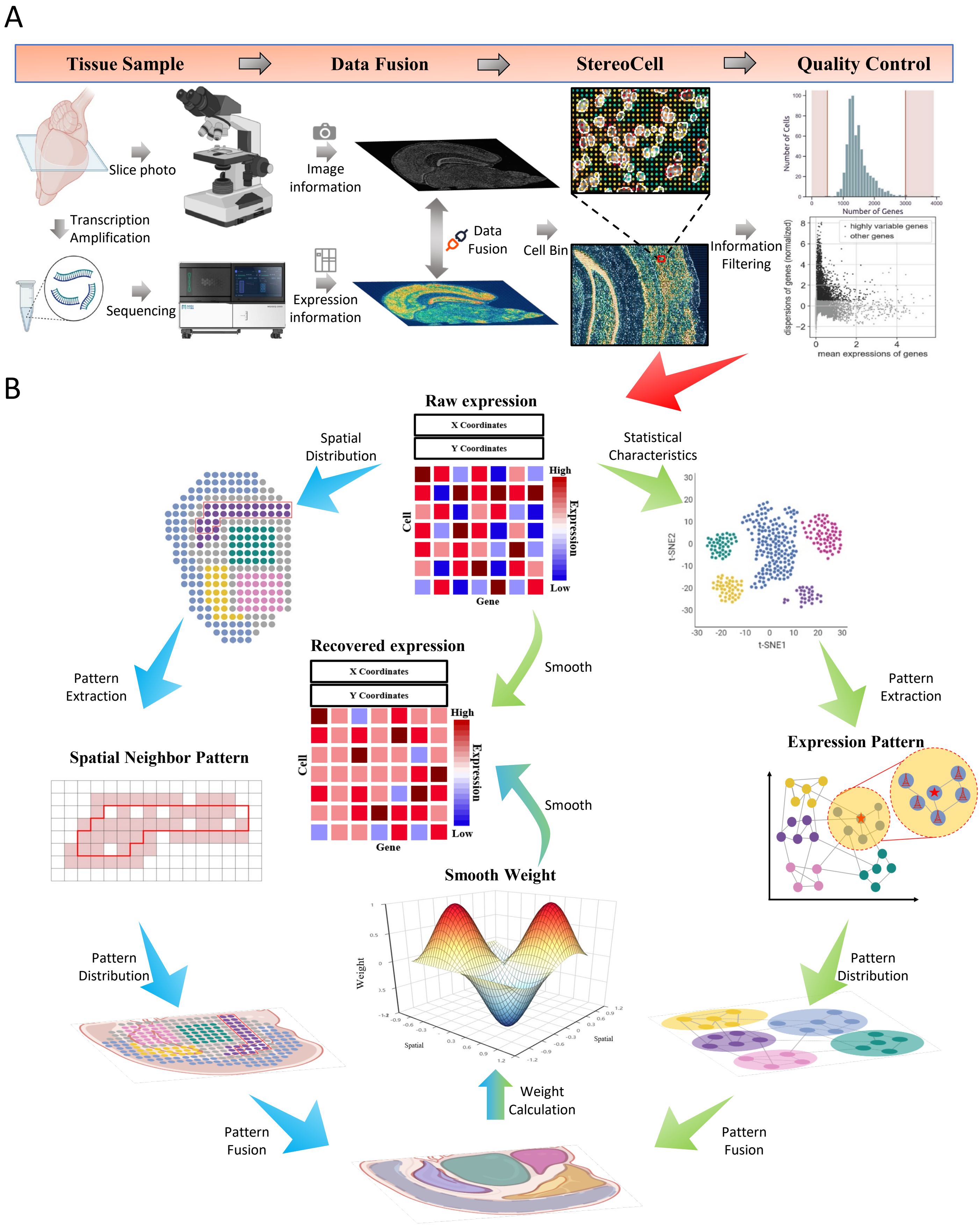

Fig. 2

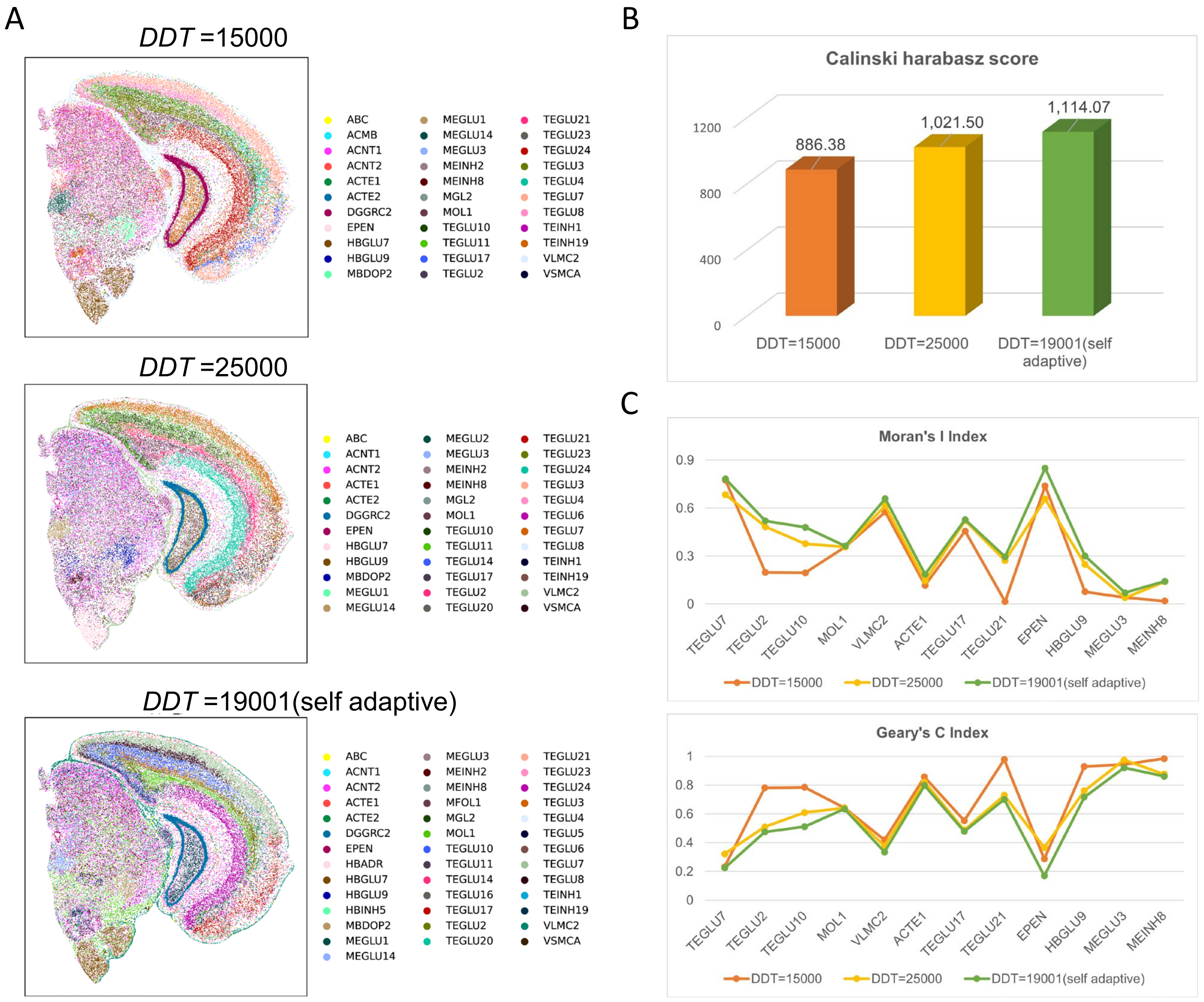
A
DDT =15000
ABC
ACMB
ACNT1
ACNT2
ACTE1
ACTE2
DGGRC2
EPEN
HBGLU7
HBGLU9
MBDOP2
MEGLU1
MEGLU14
MEGLU3
MEINH2
MEINH8
MGL2
MOL1
TEGLU10
TEGLU11
TEGLU17
TEGLU2
TEGLU21
TEGLU23
TEGLU24
TEGLU3
TEGLU4
TEGLU7
TEGLU8
TEINH1
TEINH19
VLMC2
VSMCA
DDT =25000
ABC
ACNT1
ACNT2
ACTE1
ACTE2
DGGRC2
EPEN
HBGLU7
HBGLU9
MBDOP2
MEGLU1
MEGLU14
MEGLU2
MEGLU3
MEINH2
MEINH8
MGL2
MOL1
TEGLU10
TEGLU11
TEGLU14
TEGLU17
TEGLU2
TEGLU20
TEGLU21
TEGLU23
TEGLU24
TEGLU3
TEGLU4
TEGLU6
TEGLU7
TEGLU8
TEINH1
TEINH19
VLMC2
VSMCA
DDT =19001(self adaptive)
ABC
ACNT1
ACNT2
ACTE1
ACTE2
DGGRC2
EPEN
HBADR
HBGLU7
HBGLU9
HBINH5
MBDOP2
MEGLU1
MEGLU14
MEGLU3
MEINH2
MEINH8
MFOL1
MGL2
MOL1
TEGLU10
TEGLU11
TEGLU14
TEGLU16
TEGLU17
TEGLU2
TEGLU20
TEGLU21
TEGLU23
TEGLU24
TEGLU3
TEGLU4
TEGLU5
TEGLU6
TEGLU7
TEGLU8
TEINH1
TEINH19
VLMC2
VSMCA
B
Calinski harabasz score
1200
800
400
0
886.38
1,021.50
1,114.07
DDT=15000
DDT=25000
DDT=19001(self adaptive)
C
Moran's I Index
0.9
0.6
0.3
0
TEGLU7
TEGLU2
TEGLU10
MOL1
VLMC2
ACTE1
TEGLU17
TEGLU21
EPEN
HBGLU9
MEGLU3
MEINH8
DDT=15000
DDT=25000
DDT=19001(self adaptive)
Geary's C Index
1
0.8
0.6
0.4
0.2
0
TEGLU7
TEGLU2
TEGLU10
MOL1
VLMC2
ACTE1
TEGLU17
TEGLU21
EPEN
HBGLU9
MEGLU3
MEINH8
DDT=15000
DDT=25000
DDT=19001(self adaptive)

Fig. 3

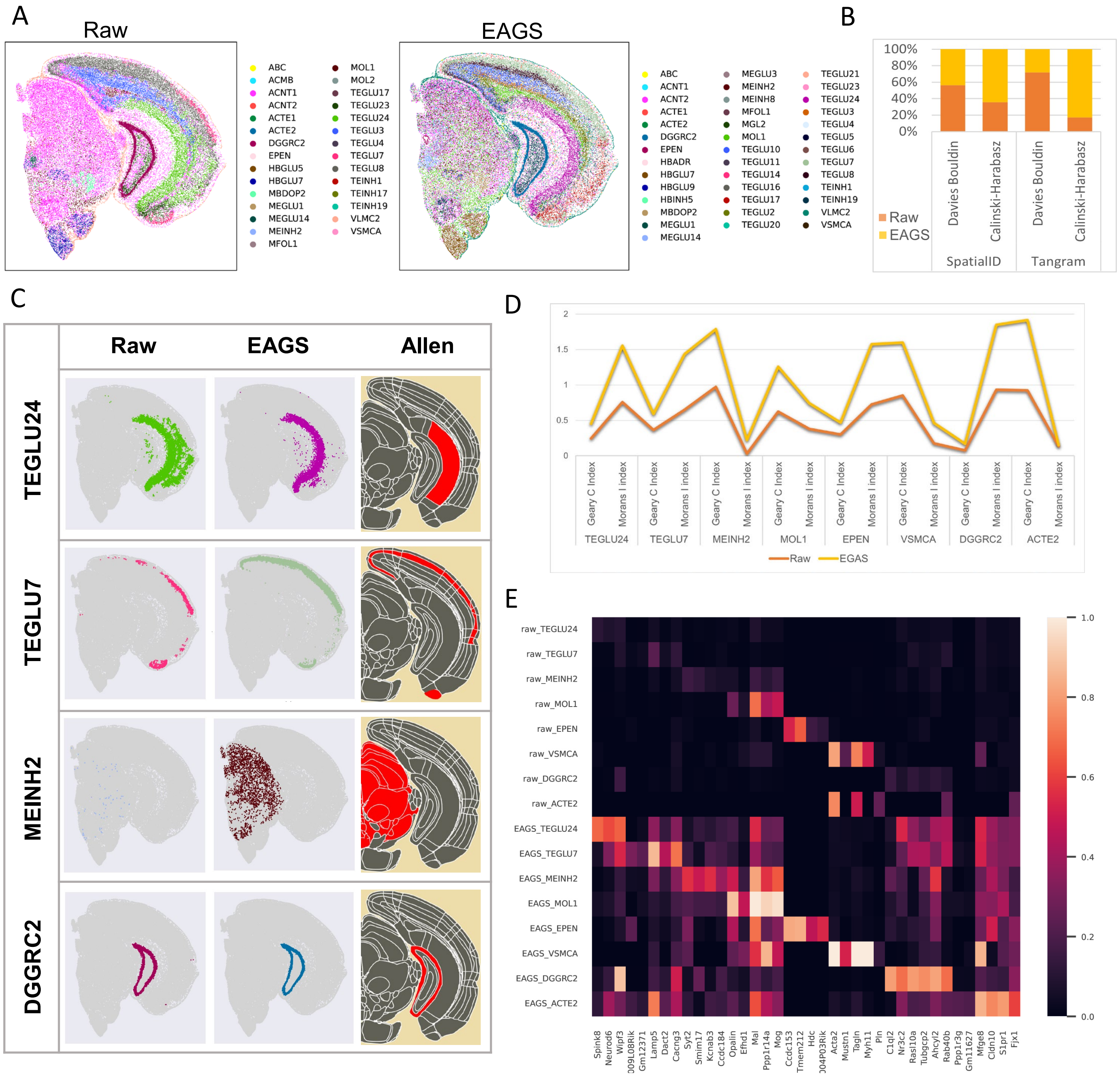
A
Raw
EAGS
B
Raw
EAGS
SpatialID
Tangram
Davies Bouldin
Calinski-Harabasz
C
Raw
EAGS
Allen
TEGLU24
TEGLU7
MEINH2
DGGRC2
D
Raw
EGAS
E

Fig. 4

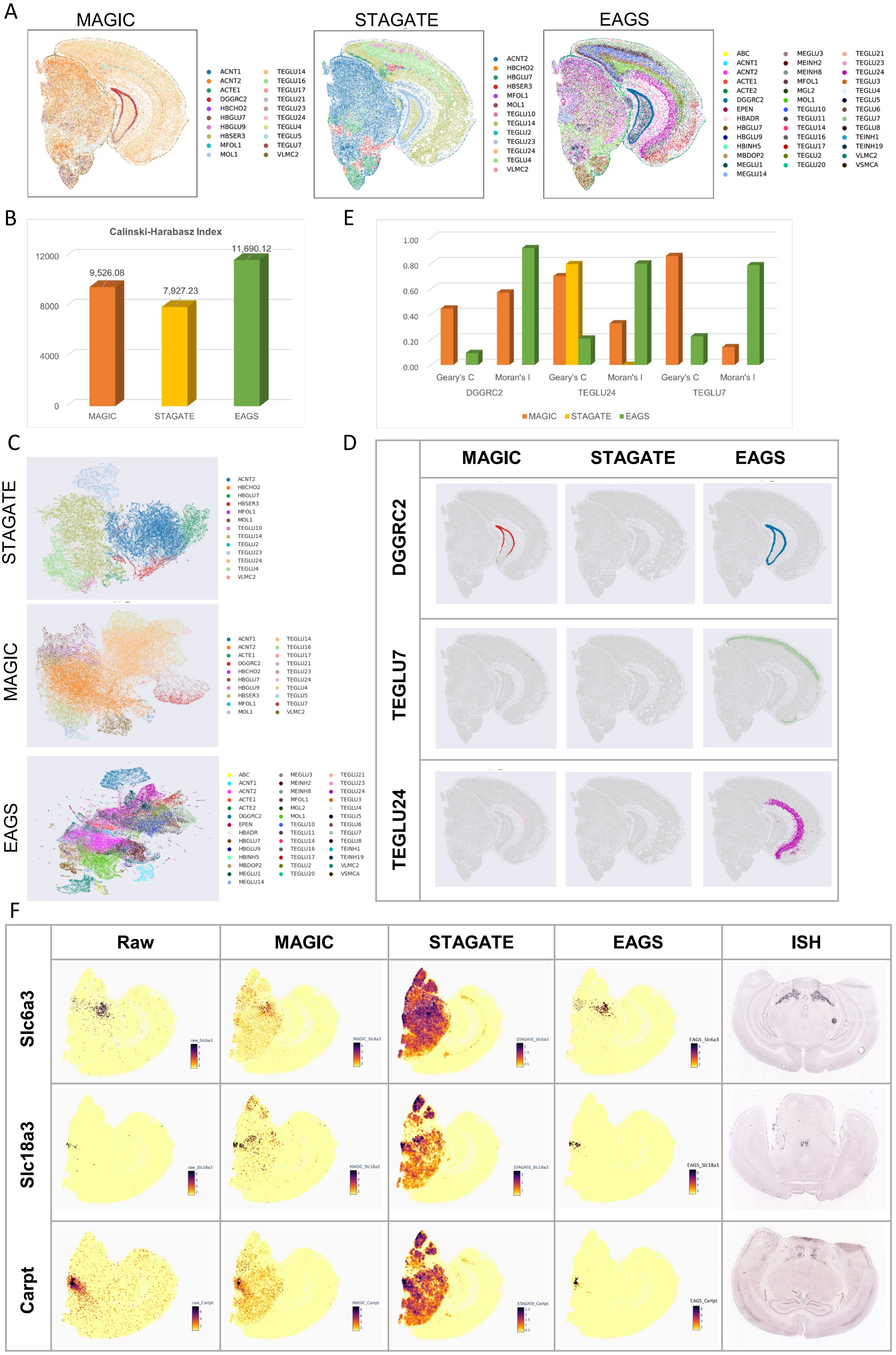
A
MAGIC
STAGATE
EAGS
B
Calinski-Harabasz Index
9,526.08
7,927.23
11,690.12
MAGIC
STAGATE
EAGS
E
Geary's C
Moran's I
DGGRC2
TEGLU24
TEGLU7
MAGIC STAGATE EAGS
C
STAGATE
MAGIC
EAGS
D
DGGRC2
TEGLU7
TEGLU24
F
Raw
MAGIC
STAGATE
EAGS
ISH
Slc6a3
Slc18a3
Carpt

Fig. 5

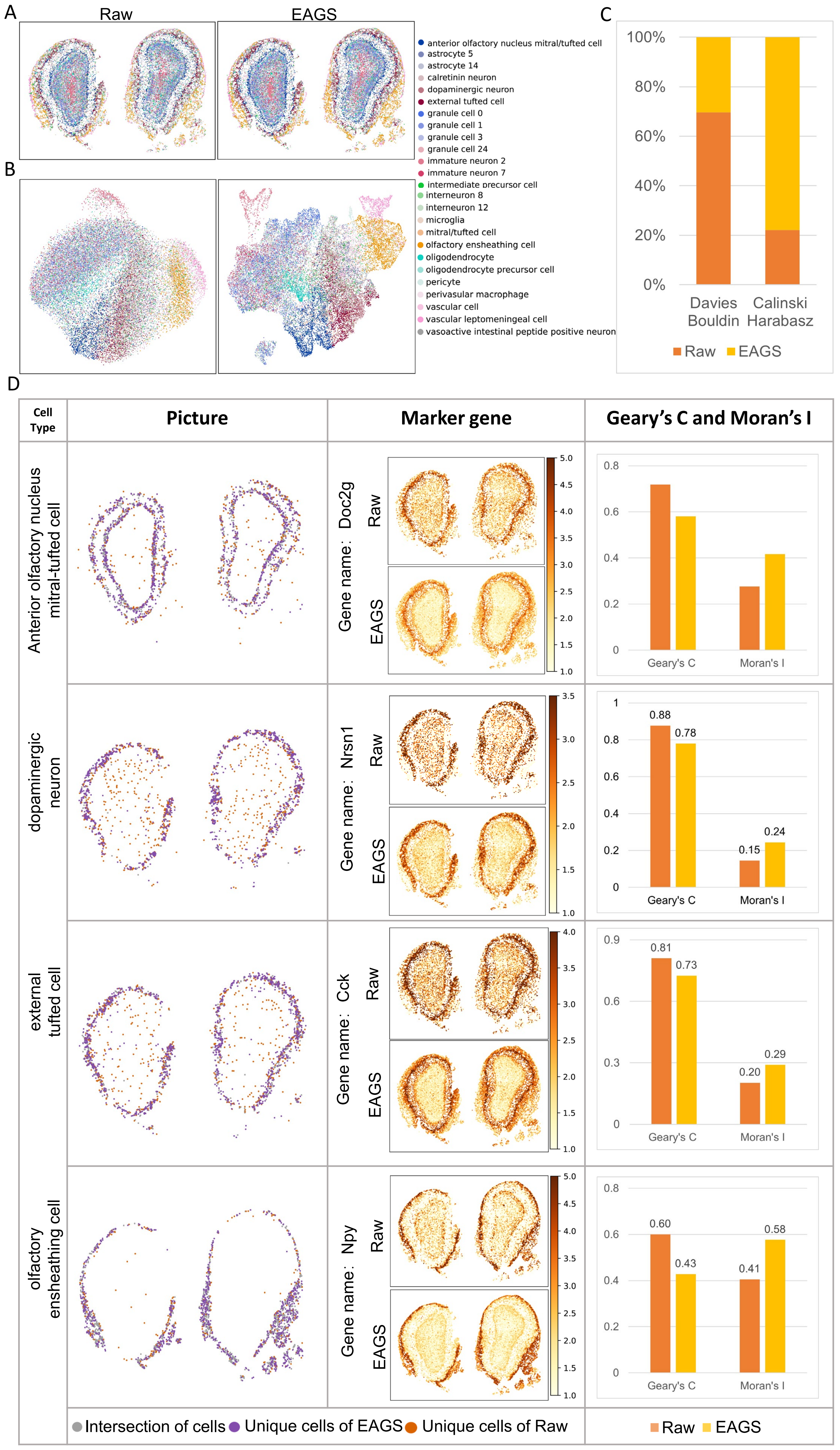
A
Raw
EAGS
B
anterior olfactory nucleus mitral/tufted cell
astrocyte 5
astrocyte 14
calretinin neuron
dopaminergic neuron
external tufted cell
granule cell 0
granule cell 1
granule cell 3
granule cell 24
immature neuron 2
immature neuron 7
intermediate precursor cell
interneuron 8
interneuron 12
microglia
mitral/tufted cell
olfactory ensheathing cell
oligodendrocyte
oligodendrocyte precursor cell
pericyte
perivasular macrophage
vascular cell
vascular leptomeningeal cell
vasoactive intestinal peptide positive neuron
C
100%
80%
60%
40%
20%
0%
Davies Bouldin
Calinski Harabasz
Raw
EAGS
D
Cell Type
Picture
Marker gene
Geary's C and Moran's I
Anterior olfactory nucleus mitral-tufted cell
Gene name: Doc2g
Raw
EAGS
dopaminergic neuron
Gene name: Nrsn1
0.88
0.78
0.15
0.24
external tufted cell
Gene name: Cck
0.81
0.73
0.20
0.29
olfactory ensheathing cell
Gene name: Npy
0.60
0.43
0.41
0.58
Geary's C
Moran's I
Intersection of cells
Unique cells of EAGS
Unique cells of Raw
Raw
EAGS
