# Supplementary material for: EAGS: efficient and adaptive Gaussian smoothing applied to high-resolved spatial transcriptomics
Source: Gigascience. 2024 Feb 20;13:giad097. doi: 10.1093/gigascience/giad097 (PMC10939424; doi:10.1093/gigascience/giad097)
Supplement: giad097_GIGA-D-23-00147_Revision_2 [file giad097_giga-d-23-00147_revision_2.pdf]

GigaScience

EAGS: efficient and adaptive Gaussian smoothing applied to high-resolved spatial transcriptomics

--Manuscript Draft--

**Manuscript Number:**	GIGA-D-23-00147R2
**Full Title:**	EAGS: efficient and adaptive Gaussian smoothing applied to high-resolved spatial transcriptomics
**Article Type:**	Technical Note
**Funding Information:**	2022YFC3400400 — Dr. Xun Xu
**Abstract:**	Background: The emergence of high-resolved spatial transcriptomics (ST) has facilitated the research of novel methods to investigate biological development, organism growth, and other complex biological processes. However, high-resolved and whole transcriptomics ST datasets require customized imputation methods to improve the signal-to-noise ratio and the data quality.<br>Findings: We propose an efficient and adaptive Gaussian smoothing (EAGS) imputation method for high-resolved ST. The adaptive two-factor smoothing of EAGS creates patterns based on the spatial and expression information of the cells, creates adaptive weights for the smoothing of cells in the same pattern, and then utilizes the weights to restore the gene expression profiles. We assessed the performance and efficiency of EAGS using simulated and high-resolved ST datasets of mouse brain and olfactory bulb.<br>Conclusions: Compared with other competitive methods, EAGS shows higher clustering accuracy, better biological interpretations, and significantly reduced computational consumption.
**Corresponding Author:**	Xun Xu, Ph.D<br>BGI-Shenzhen: BGI Group<br>Shenzhen, Guangdong CHINA
**Corresponding Author Secondary Information:**	
**Corresponding Author's Institution:**	BGI-Shenzhen: BGI Group
**Corresponding Author's Secondary Institution:**	
**First Author:**	Xun Xu, Ph.D
**First Author Secondary Information:**	
**Order of Authors:**	Xun Xu, Ph.D
	Tongxuan Lv
	Ying Zhang, Ph.D
	Mei Li
	Qiang Kang, Ph.D
	Shuangsang Fang
	Yong Zhang
	Susanne Brix
**Order of Authors Secondary Information:**	
**Response to Reviewers:**	Dear editor<br><br>First of all, we (the authors) would like to express our sincere gratitude to the editor and reviewers for their time and efforts given to our manuscript (Manuscript ID: GIGA-D-23-00147R1). Thanks a lot, and best regards.

Powered by Editorial Manager® and ProduXion Manager® from Aries Systems Corporation

	With many thanks, we would like to address the comments as below.<br><br>Comment 1: Please include a citation to your new GigaDB dataset (including the DOI link) to your reference list, and cite this by number from in the data availability section.<br>Response: We have added a citation of GigaDB dataset (including the DOI link) in the reference list and updated the data availability section.<br><br>Comment 2: Please cite the Zenodo and STOMICs datasets analogous to the gigaDB example above (i.e. each dataset should get its own entry in the bibliography, and cite them by number from the main text rather than including the URL/doi directly in the main text). Mention accession numbers where applicable.<br>Response: We have added the entries in the references for each of the Zenodo and STOMICs datasets cited in the Data Availability section with the login number.<br><br>Comment 3: Please also move any other in-text URLs for data sources to the bibliography and cite them by number in the text (similar to the GigaDB reference - please refer to our citation guidelines.<br>Response: We have moved the URLs to the References section and added the citation number.<br><br>Comment 4: please double check that all relevant accession numbers and data citations are included. (STOmicsDB_Sample: STSA0000207, STOmicsDB_Sample: STSA0000248)<br>Response: We have checked all relevant accession numbers and data citations.<br><br>Comment 5: The CNGB accession CNP0002966 is redundant and non-functional, as far as I understand - please remove this.<br>Response: We have removed this accession.<br><br>Comment 6: Please structure your abstract (“Background - Findings – Conclusions”).<br>Response: We have structured the abstract section as (“Background - Findings – Conclusions”).<br><br>Comment 7: At this stage, you can remove all highlighting/tracking of changes in red, that was made for the purpose of peer review.<br>Response: We have removed the highlighting/tracking of changes.<br><br>Comment 8: Please submit editable source files (WORD or LaTex, whatever you used - at the moment, we only have a PDF).<br>Response: We have submitted the source files of WORD.<br><br>Comment 9: If you cited preprints, please check if they are now published in a reviewed journal - if so, please cite the journal article instead. (But it's fine to cite preprints if they are not published in peer reviewed form yet).<br>Response: We have rechecked the latest publication status of preprints and have ensured that none of the preprint articles cited in the latest uploaded revision have been published in a journal.<br><br>Comment 10: Please also ensure that your revised manuscript conforms to the journal style, which can be found in the Instructions for Authors on the journal homepage.<br>Response: We have revised the manuscript to ensure that the revised manuscript conforms to the journal style.<br><br>Finally, we (the authors) would like to express thanks again sincerely to the editor and reviewers for their time and efforts spent in handing the manuscript, as well as providing us many constructive comments for improving further the presentation and quality of this manuscript.
**Additional Information:**	
**Question**	**Response**
Are you submitting this manuscript to a special series or article collection?	No

Powered by Editorial Manager® and ProduXion Manager® from Aries Systems Corporation

**Experimental design and statistics**<br><br>Full details of the experimental design and statistical methods used should be given in the Methods section, as detailed in our Minimum Standards Reporting Checklist. Information essential to interpreting the data presented should be made available in the figure legends.<br><br>Have you included all the information requested in your manuscript?	Yes
**Resources**<br><br>A description of all resources used, including antibodies, cell lines, animals and software tools, with enough information to allow them to be uniquely identified, should be included in the Methods section. Authors are strongly encouraged to cite Research Resource Identifiers (RRIDs) for antibodies, model organisms and tools, where possible.<br><br>Have you included the information requested as detailed in our Minimum Standards Reporting Checklist?	Yes
**Availability of data and materials**<br><br>All datasets and code on which the conclusions of the paper rely must be either included in your submission or deposited in publicly available repositories (where available and ethically appropriate), referencing such data using a unique identifier in the references and in the "Availability of Data and Materials" section of your manuscript.<br><br>Have you have met the above requirement as detailed in our Minimum Standards Reporting Checklist?	Yes

Manuscript
Click here to access/download;Manuscript;EAGS_for_production.docx

EAGS: efficient and adaptive Gaussian smoothing applied to high-resolved spatial transcriptomics

Tongxuan Lv[1,2,†], Ying Zhang[1,†], Mei Li[1,4,†], Qiang Kang[1,‡], Shuangsang Fang[1,3], Yong Zhang[1], Susanne Brix[4,*], Xun Xu[1,2,*]

ORCID iDs: Xun Xu [0000-0002-5338-5173]; Tongxuan Lv [0009-0008-3618-635X]; Ying Zhang [0000-0003-3830-1338]; Mei Li [0000-0003-3310-2911]; Qiang Kang [0000-0001-6579-7944]; Shuangsang Fang [0000-0002-4126-0074]; Yong Zhang [0000-0001-9950-1793]; Susanne Brix [0000-0001-8951-6705]

[1] BGI-Shenzhen, Shenzhen 518103, China

[2] College of Life Sciences, University of Chinese Academy of Sciences, Beijing 100049, China

[3] BGI-Beijing, Beijing 100101, China

[4] Department of Biotechnology and Biomedicine, Technical University of Denmark, 2800 Kgs. Lyngby, Denmark

[*] Corresponding: sbrix@dtu.dk, xuxun@genomics.cn

[†] These authors contributed equally as the first authors.

[‡] Senior author.

Abstract

Background: The emergence of high-resolved spatial transcriptomics (ST) has facilitated the research of novel methods to investigate biological development, organism growth, and other

complex biological processes. However, high-resolved and whole transcriptomics ST datasets require customized imputation methods to improve the signal-to-noise ratio and the data quality.

Findings: We propose an efficient and adaptive Gaussian smoothing (EAGS) imputation method for high-resolved ST. The adaptive two-factor smoothing of EAGS creates patterns based on the spatial and expression information of the cells, creates adaptive weights for the smoothing of cells in the same pattern, and then utilizes the weights to restore the gene expression profiles. We assessed the performance and efficiency of EAGS using simulated and high-resolved ST datasets of mouse brain and olfactory bulb.

Conclusions: Compared with other competitive methods, EAGS shows higher clustering accuracy, better biological interpretations, and significantly reduced computational consumption.

Keywords: spatial transcriptomics; imputation; gaussian smoothing; adaptive weight

Introduction

Recent advances in barcode-based spatial transcriptomics (ST) technology include 10X Visium [1], Slide-Seq [2,3], and high-definition spatial transcriptomics [4]. These advances made it feasible to provide expression profile information of entire genes, which is extremely important for comprehending biological functions and interaction networks [5,6]. High-resolved ST is an essential technical support for analyzing complex biological problems, as the function of complex biological tissues is closely related to the location of the transcriptional expression events within the tissue. However, cell localization and identification are limited by technical factors, such as the chip capture area, the sequencing depth, and the resolution. Spatially enhanced resolution transcriptome sequencing (Stereo-seq) [7] is a new ST technology based on DNA nanoballs. Stereo-seq provides the highest resolution (500 nm) among all currently available ST technologies. Such breakthrough in resolution allows researchers to perform genome-wide analyses of gene expression at the capture site (spot) with a single-cell or even sub-cellular resolution. Wang et al. [8] applied Stereo-seq to the 3D reconstruction of the ST of *Drosophila* embryos and larvae, providing a spatial- and temporal-resolved transcriptomic map of the whole organism across the developmental stages for *Drosophila* research. Liu et al. [9] reconstructed the developmental trajectory of zebrafish embryos during their development by analyzing Stereo-seq and scRNA-seq datasets from different time points.

Barcode-based high-resolved ST technology captures fewer genes at a single sequencing site (spot) than low-resolution ST technologies, such as 10X Visium [1], leading to high sparsity of the complete gene expression profile. In certain cell cycle phases, some cells do not express a set of genes whose expression thus appears to be null. In addition, amplification bias, cell cycle, library

creation, and poor RNA capture rates cause some genes to be expressed but not captured by DNA nanoballs; such genes are called "dropout" [10]. Such biases adversely affect downstream analyses, such as clustering, cellular interaction analyses, and pseudo-temporal reconstructions [11,12], when the raw data is directly processed.

Various imputation methods have been proposed to solve the "dropout" in gene expression for scRNA-seq datasets [13]. These imputation methods can be broadly classified into 3 categories according to their principles. The first category smooths or diffuses the levels of gene expression in cells with comparable expression patterns to correct (typically) all values (zero and non-zero). MAGIC imputes the missing data on scRNA-seq datasets based on the Markov chains of adjacent domains and recovers gene expression of the characterized cells by data diffusion [14]; DrImpute finds similar cells by consensus clustering and pools their gene expression values to estimate the loss [15]. The second category models the gene expression profile with an existing probabilistic statistical model to simulate the distribution of genes. SAVER assumes that each gene in each cell follows a Poisson-Gamma distribution (a negative binomial distribution) and estimates prior parameters to recover the expression of the missing genes using Poisson LASSO regression methods [16]. Scimpute constructs a mixed Gamma-Normal distribution based on the gene expression profile and uses a non-negative least squares regression model, sc-transform (R package), to perform the imputation [17]. The third category uses deep learning methods to capture the potential spatial representation of cells and reconstruct the expression matrix. DCA is an auto-encoder that predicts the parameters of the selected distribution to generate estimates [18]. These methods offer practical recommendations for single-cell imputation; however, these methods do not account for spatial information in ST datasets, and the methods based on specialized statistical models can't be applied

to the high sparsity of high-resolved ST datasets.

In recent years, ST-based imputation methods have been presented. Sprod first projects gene expression onto a potential space, connects nearest neighbor cells to construct patterns, and then learns the denoising matrix using a shared minimization of the graph's Laplacian smoothing term and reconstruction errors [19]. For ST data without pathology images, Sprod provides cluster-based pseudo-images, but it does not accurately reflect the actual cell clustering situation. STAGATE introduces a graph attention auto-encoder to construct a spatial neighbor network based on sequencing spots, and then, it introduces a distribution of the spatial neighbor network in the middle layer of the self-encoder to learn the correlation of neighboring sequencing spots and subsequently obtains the recovered gene expression profile by decoder [20]. However, the labels processed based on a specific clustering method are not completely consistent with the reality of the biological organization. It has been noted that the self-attention layer of the network does not consider the interaction between spot pairs and the information about the graphical structure of the spots [21].

To address these problems, we propose an efficient and adaptive Gaussian smoothing (EAGS) method, which is applied to high-resolved ST data. EAGS be derived from the fact that the spatial location of cells in biological tissues has a close relationship with their microenvironment, and the gene expression levels of cells within the same microenvironment are similar [17,22]. EAGS constructs different patterns based on cell expression profiles and cell location information to generate a similarity matrix. The similarity matrix then assesses cellular similarity within expression profiles to recover true biosignatures. By refining the information from proximal cells using adaptive smoothing weights and generating new gene expression profiles, the "dropout" is reduced. The resulting dataset provides RNA abundances more accurately than the original gene expression

profile and preserves more of the true biological signal. EAGS enables the usage of high-sparsity ST datasets since it is independent of prior statistical models of the expression preconditioning the gene expression profiles. More crucially, EAGS could be used for large-scale ST datasets without requiring a lot of operating memory since it does not call for the computation of parameters for a pre-defined model, skipping most of the iterative process. We applied EAGS to the simulated and high-resolved ST datasets of mouse brain and olfactory bulb, and compared it with widely used imputation methods to evaluate its efficacy in terms of fewer "zeros" in the gene expression profiles, improved cell annotation, and spatial organization replication.

Methods

The workflow of EAGS

In EAGS (RRID: SCR_024399), the original expression matrix with the single-cell resolution was first used to generate patterns based on expression and spatial information. Then, the tight relationship between cells was established using two distinct patterns. Finally, the smoothing weights calculated from the patterns were used to define the level of smoothing for each cell, and were applied to recalculate the gene expression.

Datasets

There were two methods to generate gene expression profiles from Stereo-seq *in situ* captured data. One was to acquire the spatial location information of various cells by conducting cell identification and segmentation on the optical stained image, and then match the cell in the image to the sequencing spots with spatial coordinates [7,23]. The other one was to take consecutive $X \times X$ bins as units (considered as cells), where each bin (bin$X$) contains the total gene expression of $X \times X$ spots [7]. We used the mouse brain [24] and olfactory bulb datasets at single-cell resolution [23],

which were generated by the first method and included 61,857 and 33,272 cells respectively. The In Situ Hybridization (ISH) images of the signature genes from the mouse brain were obtained to help compare the impacts of smoothing [25,26]. We also used another mouse olfactory bulb dataset generated by the second method, which contained 812 units of Bin140 [27].

The above two categories of gene expression profile with spatial information were pre-processed with the Scanpy toolbox (V1.9.1; RRID:SCR_018139) to remove low-quality signals that might be blended into the gene expression data [28,29]. For the first category, firstly, we filtered genes based on expression in at least 10 cells: those genes were kept. Next, cell outliers were filtered using gene expression: cells expressing at least 300 MID counts were kept. The 2% highest MID counts in all cells were subtracted from the overall number of MIDs across all cells in the gene expression profile. Finally, the coordinates of the spatial position information of the cells and the log-transformed and normalized gene expression profiles were employed as input to EAGS. For the second category, we filtered genes based on expression in at least 10 cells: those genes were kept. Next, cell outliers were filtered using gene expression: cells expressing at least 300 MID counts were kept.

Pattern construction

Since “similar cells” in organisms with comparable molecular microenvironments express their genes similarly, the regions with identical expression patterns may originate from the same cell type or from the same biological tissue location [17,22]. Using “similar cells” to supplement the information of a particular spot is feasible. Based on spatial location data and gene expression profiles, we constructed two patterns to divide the cells on an ST slice’s gene expression profile into several clusters. A comprehensive description of these two pattern styles is given below:

Definition 1 (Gene Expression Pattern): If $\boldsymbol{P}_e(i)$ is the gene expression domain of $\boldsymbol{Cell}_i$ for ST data, then:

$$\forall \boldsymbol{Cell}_j \in \boldsymbol{P}_e(i),\ \forall \boldsymbol{Cell}_k \in \boldsymbol{P}_g - \left(\boldsymbol{P}_e(i) \cup \{\boldsymbol{Cell}_i\}\right),\ s.t. d_{ij}{}^{e} < d_{ik}{}^{e} \tag{1}$$

where $\boldsymbol{Cell}_i$, $\boldsymbol{Cell}_j$ and $\boldsymbol{Cell}_k$ are different cells, $\boldsymbol{P}_g$ is the global pattern of gene expression, $d_{ij}{}^{e}$ and $d_{ik}{}^{e}$ are the distance between $\boldsymbol{Cell}_i$ and $\boldsymbol{Cell}_j$, and $\boldsymbol{Cell}_i$ and $\boldsymbol{Cell}_k$, respectively.

Balltree is a binary tree data structure that performs well on high-dimensional datasets, especially for Fast Nearest-neighbor Search on high-dimensional datasets [30,31]. The complete gene expression profile is separated into many different subspaces by Balltree. Then, the Euclidean distances between cells are calculated separately. Assuming the pre-normalized gene expression profile still contains m cells, the unsupervised nearest neighbor network toolkit (scikit-learn) is used to extract the n-dimensional principal component data and creates the low-dimensional information matrix ($\boldsymbol{LDIM}_{(m,n)}$) for the gene expression profile, as shown in Algorithm 1 [32]. Then, the neighboring cell matrix is constructed based on the K-Nearest Neighbors network as in Algorithm 2, forming the Expression Neighbor Matrix ($\boldsymbol{ENM}_{(m,m)}$). Different definitions are given depending on whether $\boldsymbol{Cell}_j$ can be attributed to the gene expression pattern of $\boldsymbol{Cell}_i$:

$$ENM_{(i,j)} = \begin{cases} 1,\ j = i \\ 1, \boldsymbol{Cell}_j \in \boldsymbol{P}_e(i) \\ 0, \boldsymbol{Cell}_j \notin \boldsymbol{P}_e(i) \end{cases} \tag{2}$$

where $ENM_{(i,j)}$ defines whether $\boldsymbol{Cell}_j$ is within the gene expression pattern $\boldsymbol{P}_e(i)$ of $\boldsymbol{Cell}_i$, if $ENM_{(i,j)}$ = 1, $\boldsymbol{Cell}_j$ belongs to the expression pattern of $\boldsymbol{Cell}_i$; if $ENM_{(i,j)}$ = 0, it does not.

Algorithm 1. Builds the tree structure of Balltree

Balltree is built using a divide-and-conquer method. Initially, Balltree has only one (root) node and all data points are assigned to it. At each step, the partition corresponding to each node is

split into two sub-partitions. For a partition p_i, the splitting procedure is as follows:

Step 1: Find the centroid of the node points in $\boldsymbol{LDIM}_{(m,n)}$. Reducing an n-dimensional matrix to a two-dimensional plane, the centroid of the node is centroid 1.

Step 2: Select the farthest point from centroid 1 in p_i as the first (left) child pivot p_i^L.

Step 3: Select the farthest point from p_i^L as the second (right) child pivot p_i^R.

Step 4: Assign each data point p_i to the partition whose pivot is closer.

Step 5: Assign the new sub-partitions as children of v_i in Balltree, i.e., v_i^R and v_i^L.

173

Algorithm 2. Using Balltree to find the nearest Neighbor of each cell

Input: Balltree structure $nbrs$, nearest neighbor num k, test point t, Current node n

Output: Expression Neighbor Matrix ($\boldsymbol{ENM}$)

Algorithm: $ball-tree-research(nbrs,k,t,n)$

if $distance(t, node.pivot)$ - $node.radius$ $\geq max(q)$:

    return;

if node in leaf-node set:

    Add $node.pivot$ to Q refresh q

    If $length(Q)$ > k :

        Remove the point furthest from the test point

        Refresh q

else：

    $ball-tree-research(nbrs,k,t,node.son1)$

$ball - tree - research(nbrs, k, t, node.son2)$

end if

return $\boldsymbol{ENM}$

The difference between ST and scRNA-seq datasets is that ST dataset provides the spatial coordinate position of each sequencing site (spot). After StereoCell processing, ST data are spots with a single-cell resolution where every spot corresponds to a single physical cell with spatial coordinates [23]. Cells in adjacent regions of histological sections are more likely to come from the identical microenvironment and belong to similar or identical cell types than cells from other areas. Therefore, we offer the spatial neighborhood pattern as a reference and classify the cluster of cells that are physically adjacent to a specific cell as its “spatial neighborhoods”:

Definition 2 (Spatial Neighbor Pattern): If $\boldsymbol{P}_s(i)$ is the spatial neighbor pattern of $\boldsymbol{Cell}_i$ for ST data, then:

$$\forall \boldsymbol{Cell}_j \in \boldsymbol{P}_s(i),\ s.t. d_{ij}{}^s \le \tau_s \tag{3}$$

where $d_{ij}{}^S$ is the spatial distance between $\boldsymbol{Cell}_i$ and $\boldsymbol{Cell}_j$, and τ_s represents the maximum spatial distance of $\boldsymbol{P}_s(i)$ of $\boldsymbol{Cell}_i$.

Since the spatial distribution of ST dataset is a two-dimensional plane space, the Euclidean distance can serve as a useful measure of spatial location between cells in a low-dimensional environment. Therefore, the spatial distance Matrix ($\boldsymbol{SDM}_{(m,m)}$) is constructed by computing the Euclidean distance. Furthermore, since ST chips of the Stereo-seq platform vary in size, EAGS fine-tunes the weight value for different chip sizes while calculating Euclidean distances.

Adaptive weight calculation

Cells can be used as smoothing factors for $\boldsymbol{Cell}_i$, and must satisfy both the gene expression

pattern and the spatial neighbor pattern belonging to $\boldsymbol{Cell}_i$. A cell acting as the smoothing factor is more similar in gene expression to the smoothed cell than to other cells in the overall expression profile. EAGS defines the nearest neighbor contribution matrix ($\boldsymbol{NCM}_{(m,m)}$) for an ST dataset containing m cells as follows:

$$\boldsymbol{NCM}_{(m,m)} = \boldsymbol{SDM}_{(m,m)} \bullet \boldsymbol{ENM}_{(m,m)} \tag{4}$$

where $\boldsymbol{NCM}_{(m,m)}$ is the dot product obtained by multiplying the corresponding elements of the $\boldsymbol{SDM}_{(m,m)}$ and $\boldsymbol{ENM}_{(m,m)}$ matrices. The non-zero value $\boldsymbol{NCM}_{nonzero}$ part of the $\boldsymbol{NCM}_{(m,m)}$ is selected as the parameter for smoothing weights, and $\boldsymbol{NCM}_{nonzero}$ is a *G*-dimensional row vector, where the condition $G \le M \times M$ is satisfied. The pth percentile of the $\boldsymbol{NCM}_{nonzero}$ along the specified axis is calculated by the following method:

$$(G-1) \times p^{th} = c + t \tag{5}$$

where G represents the number of vectors of $\boldsymbol{NCM}_{nonzero}$. c and t represent the integer and fractional parts of the calculation result, respectively. The Distance Distribution Threshold (DDT) is defined as follows:

$$DDT = (1-t) \times \boldsymbol{NCM}_{nonzero}[c] + t \times \boldsymbol{NCM}_{nonzero}[c+1] \tag{6}$$

where the calculated c and t obtain the pth percentile DDT along the specified axis of the $\boldsymbol{NCM}_{nonzero}$. The calculation of the adaptive weights is based on the $\boldsymbol{NCM}_{nonzero}$:

$$\boldsymbol{GS}_{new} = \mathrm{GS}(\boldsymbol{NCM}) = a \times e^{-\frac{(\boldsymbol{NCM}-b)^2}{2 \times \mu^2}} \tag{7}$$

where $\boldsymbol{GS}_{new}$ is the degree of smoothing information and is an adaptive weight determined by the degree of similarity between the cells in the pattern’s framework, and $\mathrm{GS}()$ is used to calculate the adaptive weights. The precise smoothing weight contribution between cells is calculated as follows:

$$\mu = \sqrt{-\frac{(DDT - b)^2}{2 \times \ln\left(\frac{gs}{a}\right)}} \tag{8}$$

where gs is a hyperparameter that characterizes the overall smoothness of the reference gene expression profile, which represents the overall smoothness of the entire chip. For a 1×1 cm ST chip of the Stereo-seq platform, gs is set to 0.95. μ is the smooth weight that varies around the gs, and characterizes the overall contribution level of cells in both the $\boldsymbol{P}_e(i)$ and $\boldsymbol{P}_s(i)$ to $\boldsymbol{Cell}_i$.

DDT in Eq. (6) refers to the similarity distance between cells generated based on the Gene Expression Pattern and the Spatial Neighbor Pattern in the entire gene expression distribution matrix, that as a global benchmark reference for information distribution and can be characterized as the distribution of the gene expression matrix from the overall level. μ calculated in Eq. (8) refers to the standardized parameters of the Gaussian model. The value of μ calculated by DDT can make the smoothed gene expression matrix more consistent with the preset distribution, such $\boldsymbol{GS}_{new}$ can be measured with the help of some existing expression quantities, genes are complemented without changing the overall expression profile.

Smooth

The raw gene expression profile can be processed after $\boldsymbol{GS}_{new}$ and raw expression $\boldsymbol{E}_{origin}$ have been obtained:

$$\boldsymbol{E}_{GS}(x) = \frac{\sum_{i \in P_A(i)} \boldsymbol{GS}_{new}(R(i,x)) \times \boldsymbol{E}_{origin}(i) + \boldsymbol{E}_x}{\sum_{i \in P_A(i)} \boldsymbol{GS}_{new}(R(i,x)) + 1} \tag{9}$$

where $\boldsymbol{E}_{GS}$ represents the level of gene expression after adaptive weight smoothing, $P_A(i)$ represents all cells in the region where cell x is smoothed, $\boldsymbol{E}_x$ represents the original gene expression of the smoothed cell. The whole process can be represented by Algorithm 3.

Algorithm 3. Calculate weights and perform smoothing

Input: Expression Neighbor Matrix $\boldsymbol{ENM}$, Spatial Distance Matrix $\boldsymbol{SDM}_{(m,m)}$, Origin expression matrix $\boldsymbol{E}_{origin}$, Hyperparameter gs

Output: Smooth expression Matrix $\boldsymbol{E}_{(GS)}$

Step 1：Calculating the K-nearest-neighbor cell Euclidean distance distribution.

Step 2：Smooth threshold takes the percentile value x of the distance distribution and requires a value from 0.2 to 1.

Step 3：Using Eq. (6) to back-calculate the magnitude of μ at this time; preset gs = 0.95.

Step 4：The Gaussian weights at other distances are calculated by substituting μ values into Eq. (7).

Step 5：Re-weighted summation based on the newly calculated Gaussian weights and the original expressions.

If relying entirely on the cells in the $P_A(i)$ as smoothing factors without using the origin gene expression of the smoothed $\boldsymbol{Cell}_i$, Eq. (10) can be further streamlined as:

$$\boldsymbol{E}_{GS}(x) = \frac{\sum_{i \in P_A(i)} \boldsymbol{GS}_{new}\left(R(i,x)\right) \times \boldsymbol{E}_{origin}(i)}{\sum_{i \in P_A(i)} \boldsymbol{GS}_{new}\left(R(i,x)\right)} \tag{10}$$

where $\boldsymbol{E}_{GS}$ is completely calculated from the expression level of cells in $P_A(i)$, regardless of the gene expression of $\boldsymbol{Cell}_i$.

Evaluation method

We used the imputation error by calculating the L2 norm of the difference between the smoothed matrix and ground truth (L2-error) [33]. We used Calinski-Harabasz Index (CHI) and the Davies-Bouldin Index (DBI) to evaluate the significance of the differences in intra-class and extra-

class similarity of the clustering results. We used Moran's I and Geary's C to calculate the correlation of cellular marker genes in the gene expression space of the data before and after smoothing [34].

Imputation error by calculating the L2 norm

L2-error is used to compute the difference between two matrix vectors by calculating the Euclidean distance between each corresponding element of the two matrices separately. A lower L2-error represents a higher degree of similarity between the two matrices, indicating that the method performs better. It is defined as follows:

$$\text{L2-error} = \sqrt{\sum_{i=1}^{N}\sum_{j=1}^{N}\left(Y_{i,j}\right)^2} - \sqrt{\sum_{i=1}^{N}\sum_{j=1}^{N}\left(X_{i,j}\right)^2} \quad (11)$$

where $Y_{i,j}$ represents the reference gene expression matrix, and $X_{i,j}$ represents the smoothed gene expression matrix. L2-error is mainly used to compare the difference between the reference expression matrix with "ground truth counts" and the smoothed expression matrix.

Calinski-Harabasz Index

The CHI computes the sum of squares of the distances between points in the class and the class center to determine how closely a class is related [35]. The higher the CHI, the higher the similarity between cells of the same type in the cell population, indicating that this method performs better. It is defined as:

$$\text{CHI}(k) = \frac{\text{tr}\left(\boldsymbol{B}_q\right)}{\text{tr}\left(\boldsymbol{W}_q\right)} \times \left(\frac{h-q}{q-1}\right) \quad (12)$$

where h is the number of training samples, q is the number of categories, $\boldsymbol{B}_q$ is the between-category covariance matrix, $\boldsymbol{W}_q$ is the within-category data covariance matrix, and $\text{tr}()$ is the trace calculation function.

Davies-Bouldin Index

The DBI finds the maximum by calculating the quotient of the sum of the average intra-class distances of any two classes within the sample set and the distance between the centers of the two clusters [36]. The lower the DBI, the higher the similarity between cells of the same type in the cell population, indicating that this method performs better. It is defined as:

$$\mathrm{DBI} = \frac{1}{n}\sum_{i=1}^{n}\max_{i \neq j}\left(\frac{\sigma_i + \sigma_j}{d\left(\boldsymbol{c}_i, \boldsymbol{c}_j\right)}\right) \tag{13}$$

where n is the number of categories, $\boldsymbol{c}_i$ is the center of the $i\text{th}$ category, σ_i is the average distance from all points of the $i\text{th}$ category to the center, $d\left(\boldsymbol{c}_i, \boldsymbol{c}_j\right)$ is the distance between the center points $\boldsymbol{c}_i$ and $\boldsymbol{c}_j$, and $\max()$ is the maximum function.

Moran's I

Moran's I is a global autocorrelation statistic for certain metrics on a graph. It is commonly used in spatial data analysis to evaluate autocorrelation on two-dimensional grids [37]. The higher the Moran's I, the stronger the spatial autocorrelation of the cell population, indicating that the method performs better. It is defined as:

$$\text{Moran's I} = \left(\frac{N}{W}\right) \times \frac{\sum_{i=1}^{N}\sum_{j=1}^{N}\left(w_{ij} \times \left(x_i - \bar{x}\right) \times \left(x_j - \bar{x}\right)\right)}{\sum_{i=1}^{N}\left(x_i - \bar{x}\right)^2} \tag{14}$$

where N is the number of spatial units indexed by i and j, x is the variable of interest, $\bar{x}$ is the mean of x, w_{ij} are the elements of a matrix of spatial weights with zeros on the diagonal, and W is the sum of all w_{ij}.

Geary's C

Geary's C is a measure of spatial autocorrelation that attempts to determine if observations of the same variable are spatially autocorrelated globally (rather than at the neighborhood level) [38]. The lower the Geary's C, the stronger the spatial autocorrelation of the cell population, indicating

that the method performs better. It is defined as:

$$\text{Geary's C} = \frac{(N-1)\times\sum_i\sum_j\left(\boldsymbol{w}_{ij}\times\left(x_i-x_j\right)\right)}{2\times S_0\times\sum_i\left(x_i-\bar{x}\right)^2} \tag{15}$$

where $\boldsymbol{w}_{ij}$ is the $i\text{-}th$ row of the spatial weight matrix with zeros on the diagonal, and S_0 is the sum of all the weights.

Results

Overview of EAGS

We collect the datasets of mouse brain and olfactory bulb as inputs to EAGS [23,24]. The acquisition process of these data is: stereo-seq [7] is used to capture the ST data of the mouse brain and mouse olfactory bulb *in situ* and record the position information of the sequencing spot, just like the data generation process in “Datasets” subsection, and then StereoCell [23] is used to generate ST data at single-cell resolution with spatial information. After obtaining the ST dataset at single-cell resolution, the entire gene expression profile is normalized and smoothed [39], as shown in Fig. 1A.

EAGS constructs two styles of patterns based on the input gene expression information and spatial information, respectively. These two patterns are used to identify similar cells within the pattern, as shown in Fig. 1B. Next, EAGS adaptively generates smoothing weights based on the difference between similar cells and their genes’ expression, then utilizes these weights as a reference to complement the expression of similar cells.

EAGS performs better smoothing by adaptive weighting

We use mouse brain dataset to evaluate EAGS with adaptive weight. The results are compared to the outputs of EAGS with fixed weights. As the mouse brain dataset’s adaptive weight value is

19,001, the fixed value weights are set to 25,000 and 15,000. We use Spatial-ID to annotate cell types in order to assess the potential of EAGS to improve the cell annotation power and restore the true levels of gene expression [24]. Fig. 2 shows all the results of the subsequent analysis with the adaptive and the fixed weights. The cell annotation results of EAGS using an adaptive weight compares to a fixed weight generated a cell-type spatial map with clearer tissue outlines and more annotated cell-type subtypes (Fig. 2A).

Based on our cell annotation results, the CHI of the EAGS smoothing results with adaptive and fixed weights are calculated. Next, Geary's C and Moran's I of the common cell types in the annotation results are calculated (Figs. 2B and C). The results based on the adaptive weight cell annotation show a significant improvement in spatial autocorrelation compared to the others. Also, within the same type of cell annotation, the level of intra-class autocorrelation is higher.

EAGS smooths gene expression with better performance on simulated ST dataset

We collected Bin140 specification mouse olfactory bulb ST dataset [27], the top 2000 highly variable genes are selected as the reference input of ScDesign3 to construct a simulation space group with "ground truth counts" [40]. To simulate the "dropout" phenomenon during the sequencing process, we randomly drop the simulated ST dataset expression to varying degrees and add different proportions of noise. EAGS, MAGIC [14], kNN-smoothing [41], SPCS [22] and STAGATE [20] are used to impute the processed ST dataset, then L2-error with the "ground truth counts" matrix and DBI are calculated respectively. The results are shown in Table 1.

Table 1. Results on simulated ST dataset with different proportions of dropout and noise

Dataset	Method	10% noise		20% noise		30% noise	
		L2-error	DBI	L2-error	DBI	L2-error	DBI

	MAGIC	473.0679	4.7818	524.2593	4.4600	562.8816	4.2533
	kNN-smoothing	381.4241	4.4481	448.7622	4.2175	504.8468	3.9928
30% dropout	SPCS	322.4659	4.6961	390.2348	4.3379	460.2699	4.2569
	STAGATE	757.3749	11.3146	790.5977	10.4536	791.5948	4.7064
	EAGS	**313.4211**	**4.1926**	**379.4374**	**3.9912**	**449.6919**	**3.9589**
	MAGIC	496.2557	4.9018	557.0938	4.4717	590.9306	4.2881
	kNN-smoothing	389.2189	5.2899	458.8456	4.4637	517.1581	4.7092
50% dropout	SPCS	319.4318	4.8357	398.8399	4.3223	475.0918	4.2884
	STAGATE	705.9759	8.7298	747.5357	76.9083	872.2147	9.3826
	EAGS	**310.2873**	**4.6808**	**386.7170**	**4.3201**	**459.1241**	**4.2065**
	MAGIC	506.0679	6.3969	571.8875	5.9789	600.8655	5.7213
	kNN-smoothing	390.3494	7.7208	477.8630	6.6270	532.8365	6.1928
70% dropout	SPCS	321.1818	**5.9028**	413.9192	5.7685	488.5445	5.6784
	STAGATE	850.6077	13.1108	814.2477	15.8843	693.4574	7.2281
	EAGS	**312.1247**	6.0768	**395.1362**	**5.6497**	**467.0692**	**5.6410**

From Table 1, in the simulated datasets with 30%- and 50%-dropout, L2-error and DBI values obtained by EAGS are always the lowest, regardless of the proportion of noise. When the proportion of dropout is 70%, DBI obtained by EAGS is suboptimal with 10%-noise (only higher than that of SPCS), and the results obtained by EAGS are the best on the other cases. In general, EAGS performs better on different simulated ST datasets and shows obvious advantages in improving intra-cell similarity and consistency with the “ground truth counts” compared with other methods.

EAGS smooths gene expressions for better characterizing the spatial expression

patterns of mouse brain

We perform cell annotation on mouse brain data before and after EAGS smoothing using Spatial-ID [24]. The annotation results are shown in Fig. 3A. The mouse brain cell annotation based on data smoothed by EAGS return a clearer tissue structure, and more cell types can be annotated. To further assess the improvement provided by EAGS in cell annotation, we also perform cell annotation with Tangram [42], a technique for merging spatial data types with single cell/single nucleus RNA sequencing data and for cell type annotation. As shown in Fig. 3B, the CHI and DBI are calculated for the spatial autocorrelation of cell types with the gene expression profiles after Tangram and Spatial-ID cell annotation. These results show that EAGS smoothing provides significantly better results in cell-type annotations.

Fig. 3C shows the results of cell annotation using Spatial-ID and the spatial map of Allen Mouse Brain Atlas of corresponding cell types [25,26]. TEGLU24, TEGLU7 and MEINH2 are important cell types in the Hippocampus, Cortex and Midbrain dorsal respectively, and DGGRC2 is the important cell type in the Midbrain ventral and Dentate gyrus. These cell types are more consistent with the Allen's spatial expression map of cell types after EAGS smoothing. To verify the smoothing effect, Moran's I and Geary's C are calculated for cells with different cell number ratios using the raw or the EAGS smoothed dataset (Fig. 3D). To determine whether the correlation between the above cell types and their marker genes improved after smoothing, the ratios of the number of annotated cell types to their corresponding non-zero marker gene expressions are computed. The ability of EAGS to restore true biological signals is shown in Fig. 3E. Our results show that EAGS contributes to enhancing the cellular features of the mouse brain as well as the spatial autocorrelation and intraclass similarity of the gene expression patterns.

EAGS improves spatial patterns and downstream analyses of gene expression data

EAGS is compared with the imputation methods, MAGIC [14], STAGATE [20] and kNN-smoothing [41], on ST mouse brain dataset (SPCS can't be executed successfully because the sparsity of this dataset is high to cause out of memory, thus the results SPCS are not obtained). The cell type space map of different imputation methods using Spatial-ID as reference are shown in Fig. 4A (left). EAGS return more cell types and more prominent outlines than other methods. The results of MAGIC are very unbalanced in terms of the number of cell types, with a large number of cell annotations that did not match the true values [25,26]. The annotations of the Midbrain dorsal, the Midbrain ventral, and the Dentate gyrus are mixed using MAGIC. The results of STAGATE show fewer cell types. Also, STAGATE don't result in well-organized cell type distributions in the Hippocampus and Cortex. The cell type boundaries of cell annotation after kNN-smoothing processing are blurred, and different types of cells are mixed. In order to avoid the impact of data sparsity on the interpretability of the results, the input data of the cell annotation is the 50th-dimensional principal component of different imputation results; the Uniform Manifold Approximation and Projection (UMAP) of the annotated results is shown in Fig. 4A (middle). The cell type space maps, consisting of cell types that are highly represented and annotated by the three methods, are shown in Fig. 4A (right). Fig. 4B shows the CHI derived from data processed using one of the four methods. After cell annotation, CHI [35] calculated by the cell annotation label using EAGS shows higher spatial autocorrelation than other three methods. EAGS obtain higher Moran's I and Geary's C than other methods (Fig. 4C). Additionally, the spatial maps of a few marker genes based on their expression are generated (Fig. 4D). The gene expression profiles smoothed by EAGS agree with the Allen's ISH image better than the other methods.

To evaluate the efficiency of high-resolved ST data, we run EAGS, MAGIC, STAGATE, kNN-smoothing, Scimpute and Drimpute three times on ST mouse brain dataset and monitor the average run time. STAGATE is run using a GPU. For the sake of fairness, in this running time comparison, all methods used the CPU uniformly. EAGS require the shortest run time, taking 3,484 seconds, while MAGIC takes 4,109 seconds, kNN-smoothing costs 4,739 seconds, and the other methods need a large memory consumption and can't reach their final output in an acceptable time. The ST mouse brain dataset for STAGATE imputation is generated utilizing a GPU platform.

EAGS application to high-resolved ST dataset of other biological tissues

To verify EAGS's adaptability to high-resolved ST data, we next apply EAGS to mouse olfactory bulb dataset. We generate the mouse olfactory bulb spatial cell map with cell type annotations (Fig. 5A) and the UMAP with cell annotation labels (Fig. 5B). The cell-annotated spatial map of the EAGS results show a clearer outline of the cells in the mouse olfactory bulb (Fig. 5A). The results of EAGS in UMAP form easily distinguishable clusters in the transcriptome space, and the clusters of different cell types have a low degree of overlap (Fig. 5B). We then calculate the CHI and DBI of the results generated without and with EAGS. EAGS can generate the results with higher intraclass similarity. Also, cells belonging to the same annotation type are closer to each other when the data has been smoothed by EAGS (Fig. 5C). Next, we count the cell types with a high proportion of Tangram cell labels to generate a spatial cell map and make a heatmap of the expression of the corresponding marker genes for different types of cells (Fig. 5D). Then we classify the sources of different cell labeling results and calculated Geary's C and Moran's I. The cell type annotation profile generated through dataset smoothed by EAGS is clearer. Also, the corresponding marker gene expression is more concentrated, and the cell types have higher Geary's C and Moran's I if the

data has been processed using EAGS. These results indicate a stronger spatial autocorrelation in the transcriptome space.

Discussions

EAGS defines patterns based on expression and spatial information. Specifically, it selects similar elements from the intersection between cells of two patterns, ensuring a reliable source of information is borrowed between smoothed cells and similar cells. The main source of smoothing information for EAGS is the smoothing weights adaptively generated based on gene expression profiles. EAGS considers the overall expression level to generate weights, avoids the appearance of a single edge value, and effectively ensures the reliability of information borrowed between cells. This allows to recover authentic cellular signals with improved intracellular similarity and spatial autocorrelation. For example, the expression of the Cartpt gene in Fig. 4D is scattered in the original data heatmap, with more noise appearing, and the matching degree with Allen's ISH image is low. EAGS smoothing consider the reliability of adjacent information. After EAGS smoothing, a lot of noise is eliminated, and more Cartpt genes are expressed in the correct cells, which is more degree with Allen's ISH image and the aggregation of Cartpt expression is significantly improved. Furthermore, EAGS improves the quality of raw data as it recovers the original biological signals by smoothing cell expression information. The dimensional space is adjusted to ensure the hidden correlation between cells. As it does not depend on a specific statistical model, EAGS does not adjust from the low-dimensional space of the expression profile, thus ensuring the hidden correlation between cells. More importantly, EAGS does not require pre-defined expression models, numerous iterations to obtain the model parameters, or multiple training sessions on the deep learning model framework of the GPU platform. Consequently, EAGS significantly reduces

computational costs and offers a significant execution advantage over other methods. Finally, because of the general applicability of smoothing, EAGS is suitable for different ST data.

It should be noted that the EAGS model is based on the premise that “neighboring” cells in the spatial microenvironment of biological tissues are more similar, which is applicable to most developmental tissue systems. However, for complex microenvironments with high biological heterogeneity (such as tumor microenvironment, etc.), this assumption will be challenged. EAGS may result in many false positive signals. When it is necessary to perform EAGS on complex tumor microenvironment samples, when calculating the adaptive Gaussian smoothing weight, the sample may need to be partitioned according to different situations, and gaussian weight is calculated for different areas.

Conclusions

We propose EAGS, a method for smoothing high-resolved ST datasets that performs two-factor smoothing and adaptive weighting on raw gene expression profiles. EAGS significantly improves computing efficiency, reduces “dropout” in ST data, recovers the expression of true biological signals, and restores the spatial patterns of tissues. In the future, we will explore the false positive signals produced by EAGS imputation strategies, as well as downstream analyses of datasets after imputation.

Availability of Source Code and Requirements

Project name: EAGS: efficient and adaptive Gaussian smoothing

Project home page: https://github.com/STOmics/EAGS

Operating system(s): Platform independent

Programming language: Python

Other requirements: Python 3.8 or higher

License: MIT License

RRID: SCR_024399

BiotoolsID: EAGS

Data availability

The mouse brain dataset at single-cell resolution is available in STOmics DB of China National Gene Bank (CNGB) (accession code: “STT0000022”) [43], [44]. The mouse olfactory bulb at single-cell resolution is available in STOMICS DataBase (accession code: “STT0000027”) [43], [44]. The mouse olfactory bulb data for the Bin140 specification is available in China National Gene Bank (CNGB) (accession code: “CNP0001543”) [7]. The ST data at single-cell resolution with spatial information is available in Zenodo [45]. An archival copy of the code and supporting data is available via the GigaScience repository, GigaDB [46].

Funding

This work was supported by the National Key R&D Program of China (2022YFC3400400).

Authors’ contributions

Project administration and supervision: Xun Xu, Susanne Brix

Algorithm development and implementation: Tongxuan Lv, Ying Zhang

Data collection, processing, and application: Tongxuan Lv, Mei Li, Qiang Kang

Project coordination: Mei Li, Shuangsang Fang, Yong Zhang

Method comparisons: Tongxuan Lv, Qiang Kang

Manuscript writing and figure generation: Tongxuan Lv, Qiang Kang

Manuscript review: Tongxuan Lv, Mei Li, Qiang Kang, Shuangsang Fang

Competing interests

The authors declare they have no competing interests.

Abbreviations

CHI: Calinski-Harabasz Index; DBI: Davies-Bouldin Index; DDT: Distance Distribution Threshold; EAGS: efficient and adaptive Gaussian smoothing; MIDs: molecular identifiers; ST: spatial transcriptomics; Stereo-seq: spatially enhanced resolution transcriptome sequencing. scRNA-seq: single-cell RNA seq; ISH: In Situ Hybridization; UMAP: uniform manifold approximation and projection.

Acknowledgments

We thank Guangdong Provincial Key Laboratory of Genome Read and Write (2017B030301011) for technical support for this study. We thank China National GeneBank for providing data support for this study.

References

1. Ji AL, Rubin AJ, Thrane K, et al. Multimodal Analysis of Composition and Spatial Architecture in Human Squamous Cell Carcinoma. Cell 2020;182(2):497-514. doi:10.1016/j.cell.2020.05.039.

2. Rodriques SG, Stickels RR, Goeva A, et al. Slide-seq: A scalable technology for measuring genome-wide expression at high spatial resolution. Science 2019;363(6434):1463-7. doi:10.1126/science.aaw1219.

3. Stickels RR, Murray E, Kumar P, et al. Highly sensitive spatial transcriptomics at near-cellular resolution with Slide-seqV2. Nat Biotechnol 2021;39:313-9. doi:10.1038/s41587-020-0739-1.

4. Vickovic S, Eraslan G, Salmén F, et al. High-definition spatial transcriptomics for in situ tissue profiling. Nat Methods 2019;16:987-90. doi:10.1038/s41592-019-0548-y.

5. Fang S, Chen B, Zhang Y, et al. Computational Approaches and Challenges in Spatial Transcriptomics. Genom Proteom Bioinf 2022. doi:10.1016/j.gpb.2022.10.001.

6. Longo SK, Guo MG, Ji AL, et al. Integrating single-cell and spatial transcriptomics to elucidate intercellular tissue dynamics. Nat Rev Genet 2021;22:627-44. doi:10.1038/s41576-021-00370-8.

7. Chen A, Liao S, Cheng M, et al. Spatiotemporal transcriptomic atlas of mouse organogenesis using DNA nanoball-patterned arrays. Cell 2022;185(10):1777-92. doi:10.1016/j.cell.2022.04.003.

8. Wang M, Hu Q, Lv T, et al. High-resolution 3D spatiotemporal transcriptomic maps of developing Drosophila embryos and larvae. Dev Cell 2022;57(10):1271-83.e4. doi: 10.1016/j.devcel.2022.04.006.

9. Liu C, Li R, Li Y, et al. Spatiotemporal mapping of gene expression landscapes and developmental trajectories during zebrafish embryogenesis. Dev Cell 2022;57(10):1284-98.e5. doi:10.1016/j.devcel.2022.04.009.

10. Kharchenko PV, Silberstein L, Scadden DT. Bayesian approach to single-cell differential expression analysis. Nat Methods 2014;11(7):740-2. doi:10.1038/nmeth.2967.

11. Ly LH, Vingron M. Effect of imputation on gene network reconstruction from single-cell RNA-seq data. Patterns (N Y) 2021;3(2):100414. doi:10.1016/j.patter.2021.100414.

12. Xu J, Cui L, Zhuang J, et al. Evaluating the performance of dropout imputation and clustering methods for single-cell RNA sequencing data. Comput Biol Med 2022;146:105697. doi:10.1016/j.compbiomed.2022.105697.

13. Hou W, Ji Z, Ji H, et al. A systematic evaluation of single-cell RNA-sequencing imputation methods. Genome Biol 2020;21:218. doi:10.1186/s13059-020-02132-x.

14. Dijk D, Sharma R, Nainys J, et al. Recovering Gene Interactions from Single-Cell Data Using Data Diffusion. Cell 2018;174(3):716-29.e27. doi:10.1016/j.cell.2018.05.061.

15. Gong W, Kwak IY, Pota P, et al. DrImpute: Imputing dropout events in single cell RNA sequencing data. BMC Bioinformatics 2018;19(1):220. doi:10.1186/s12859-018-2226-y.

16. Huang M, Wang J, Torre E, et al. SAVER: gene expression recovery for single-cell RNA sequencing. Nat Methods 2018;15(7):539-42. doi:10.1038/s41592-018-0033-z.

17. Li WV, Li JJ. An accurate and robust imputation method scImpute for single-cell RNA-seq data. Nat Commun 2018;9:997. doi:10.1038/s41467-018-03405-7.

18. Eraslan G, Simon LM, Mircea M, et al. Single-cell RNA-seq denoising using a deep count autoencoder Nat Commun. 2019;10:390. doi:10.1038/s41467-018-07931-2.

19. Wang Y, Song B, Wang S, et al. Sprod for de-noising spatially resolved transcriptomics data based on position and image information Nat Methods. 2022;19:950–8. doi:10.1038/s41592-022-01560-w.

20. Dong K, Zhang S. Deciphering spatial domains from spatially resolved transcriptomics with an adaptive graph attention auto-encoder. Nat Commun 2022;13:1739. doi:10.1038/s41467-022-29439-6.

21. Park W, Chang W, Lee D, et al. Graph Self-Attention for learning graph representation with Transformer. arXiv 2022;2201.12787. doi:10.48550/arXiv.2201.12787.

22. Liu Y, Wang T, Duggan B, et al. SPCS: a spatial and pattern combined smoothing method for spatial transcriptomic expression. Brief Bioinform 2022;23(3):bbac116. doi:10.1093/bib/bbac116.

23. Li M, Liu H, Li M, et al. StereoCell enables highly accurate single-cell segmentation for spatial transcriptomics. bioRxiv 2023;530414. doi: 10.1101/2023.02.28.530414.

24. Shen R, Liu L, Wu Z, et al. Spatial-ID: a cell typing method for spatially resolved transcriptomics via transfer learning and spatial embedding. Nat Commun 2022;13:7640. doi:10.1038/s41467-022-35288-0.

25. Lein ES, Hawrylycz MJ, Ao N, et al. Genome-wide atlas of gene expression in the adult mouse brain. Nature 2007;445:168–76. doi:10.1038/nature05453.

26. Zeisel A, Hochgerner H, Lönnerberg P, et al. Molecular Architecture of the Mouse Nervous System. Cell. 2018;174(4):999-1014.e22. doi:10.1016/j.cell.2018.06.021.

27. Zhang C, Liu L, Zhang Y, et al. spatiAlign: An Unsupervised Contrastive Learning Model for Data Integration of Spatially Resolved Transcriptomics. bioRxiv 2023;08.08.552402. doi: 10.1101/2023.08.08.552402.

28. Virshup I, Bredikhin D, Heumos L, et al. The scverse project provides a computational ecosystem for single-cell omics data analysis. Nat Biotechnol 2023;41:604–6. doi: 10.1038/s41587-023-01733-8.

29. Wolf FA, Angerer P, Theis FJ. SCANPY: Large-scale single-cell gene expression data analysis. Genome Biol 2018;19(1):15. doi:10.1186/s13059-017-1382-0.

30. Omohundro SM. Five balltree construction algorithms. International Computer Science Institute Technical Report; 1989.

31. Kumar N, Zhang L, Nayar S. What is a good nearest neighbors algorithm for finding similar patches in images? In European Conference on Computer Vision 2008;364–78. doi:10.1007/978-3-540-88688-4_27.

32. Pedregosa F, Varoquaux G, Gramfort A, et al. Scikit-learn: Machine learning in Python. J Mach Learn Res, 2011;12:2825-30.

33. Chen S, Yan X, Zheng R, Li M. Bubble: a fast single-cell RNA-seq imputation using an autoencoder constrained by bulk RNA-seq data. Brief Bioinform 2023;24:1. doi: 10.1093/bib/bbac580.

34. Desgraupes B. Clustering Indices. Univ.Paris Ouest-Lab Modal'X 2013;1(34).

35. Caliñski T, Harabasz J. A Dendrite Method Foe Cluster Analysis. Commun Stat 1974;3:1,1-27. doi:10.1080/03610927408827101.

36. Hubert L, Arabic P. Comparing Partitions. J Classif 1985;2:193-218. doi:10.1007/BF01908075.

37. Moran PAP. Notes on Continuous Stochastic Phenomena. Biometrika 1950;37(1/2):17-23. doi:10.2307/2332142.

38. Geary RC. The Contiguity Ratio and Statistical Mapping. Statistician. 1954.

39. Chen G, Ning B, Shi T. Single-Cell RNA-Seq Technologies and Related Computational Data Analysis. Front Genet 2019;10:317. doi:10.3389/fgene.2019.00317.

40. Song D, Wang Q, Yan G, et al. scDesign3 generates realistic in silico data for multimodal single-cell and spatial omics. Nat Biotechnol 2023; Nature Research; 2023;1-6. doi: https://doi.org/10.1038/s41587-023-01772-1.

41. Wagner F, Yan Y, Yanai I. K-nearest neighbor smoothing for high-throughput single-cell RNA-Seq data 2. bioRxiv 2018;217737. doi: 10.1101/217737.

42. Biancalani T, Scalia G, Buffoni L, et al. Deep learning and alignment of spatially resolved single-cell transcriptomes with Tangram. Nat Methods 2021;18:1352–62. doi:10.1038/s41592-021-01264-7.

43. Xu Z, Wang W, Yang T, et al. STOmicsDB: a database of spatial transcriptomic data[J]. bioRxiv 2022;2022.03.11.481421. doi: 10.1101/2022.03.11.481421.

44 STOmics DB: Spatial Transcript Omics DataBase (STOmics DB) https://db.cngb.org/stomics/ accessed 13/10/2023

45. Lv T, Zhang Y, Li M, et al. EAGS: efficient and adaptive gaussian smoothing applied to high-resolved spatial transcriptomics (Version 1) [Data set]. Zenodo 2023. doi: https://doi.org/10.5281/zenodo.7906815.

46 . Xu X, Lv T, Zhang Y, et al. Supporting data for "EAGS: efficient and adaptive Gaussian smoothing applied to high-resolved spatial transcriptomics." GigaScience Database 2023. http://dx.doi.org/10.5524/102457.

Figure Legends

Figure 1: Workflow of EAGS. (A) Data generation process for the input of EAGS. (B) The EAGS method calculates the nearest neighbor information based on the gene expression pattern and spatial information. Then, EAGS adaptively generates smoothing weights and outputs the smoothed results.

Figure 2: Results of EAGS with adaptive and fixed weights. (A) Spatial cell type map for cell annotation with Spatial-ID using different weights for smoothing results. (B) The smoothing results with different weights are annotated with Spatial-ID cells. The Calinski-Harabasz Index is calculated using cell labels. (C) After the cell annotation using Spatial-ID with different weights, Geary's C and Moran's I are calculated from annotation results.

Figure 3: Comparisons between the analysis results obtained from data before and after EAGS smoothing. (A) Spatial cell type maps of the mouse brain using Spatial-ID cell annotation of raw and EAGS smoothed data. (B) Davies-Bouldin and Calinski-Harabasz Indexes calculated using Spatial-ID and Tangram annotation results obtained from raw and EAGS smoothed data. (C) Comparison of the spatial map and Allen Mouse Brain Atlas obtained from raw and EAGS

smoothed dataset. (D) Comparison of Moran’s I and Geary’s C cell annotation types obtained from raw and EAGS smoothed dataset. (E) Heatmap of non-zero ratio between the number of cell types and their marker genes obtained from raw and EAGS smoothed dataset.

Figure 4: Comparison of different imputation methods. (A) left: Spatial maps of cell types using Spatial-ID cell annotations and four different imputation methods. middle: UMAP dimensionality reduction using Spatial-ID cell annotation and different imputation methods. right: Individual cell type spatial maps after cell annotation and different imputation methods. (B) Calinski-Harabasz Index calculated using cell labels after Spatial-ID cell annotations and different imputation methods. (C) Moran’s I and Geary’s C for the DGGRC2, TEGLU7, TEGLU24 cell types. (D) Marker gene heatmaps and Mouse Brain Atlas obtained using different imputation methods.

Figure 5: EAGS application to mouse olfactory bulb data. (A) Cell-annotated spatial map of data before and after EAGS smoothing. (B) Cell-annotated Umap of dataset before and after EAGS smoothing (C) Davies-Bouldin and Calinski-Harabasz Indexes of mouse olfactory bulb data. (D) How the annotation results of the main cell types of the mouse olfactory bulb differ between data without and with EAGS smoothing. We also show the heatmap of the marker genes of different cell types, and Moran’s I and Geary’s C indexes of the corresponding types. Cells annotated before and after smoothing (grey), cells annotated by EAGS alone (purple), and cells annotated by pre-treatment data alone (orange) are displayed on the left side; the expression heatmap of marker genes corresponding to different cell types are shown on the middle; the Moran’s I and Geary’s C indices are shown on the right side.

Fig_1

Click here to access/download;Figure;Fig_1.pdf

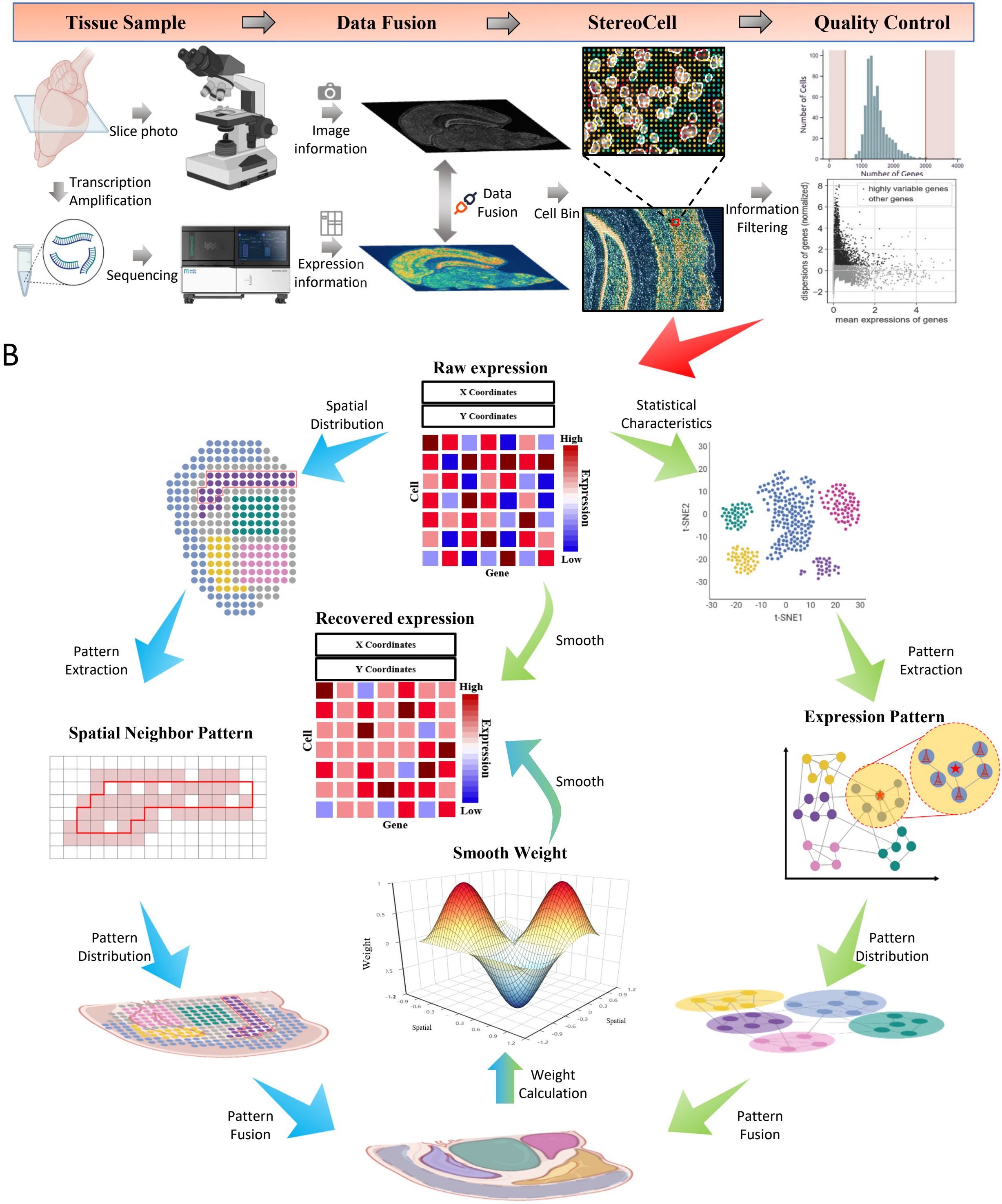

Fig_2

Click here to access/download;Figure;Fig_2.pdf

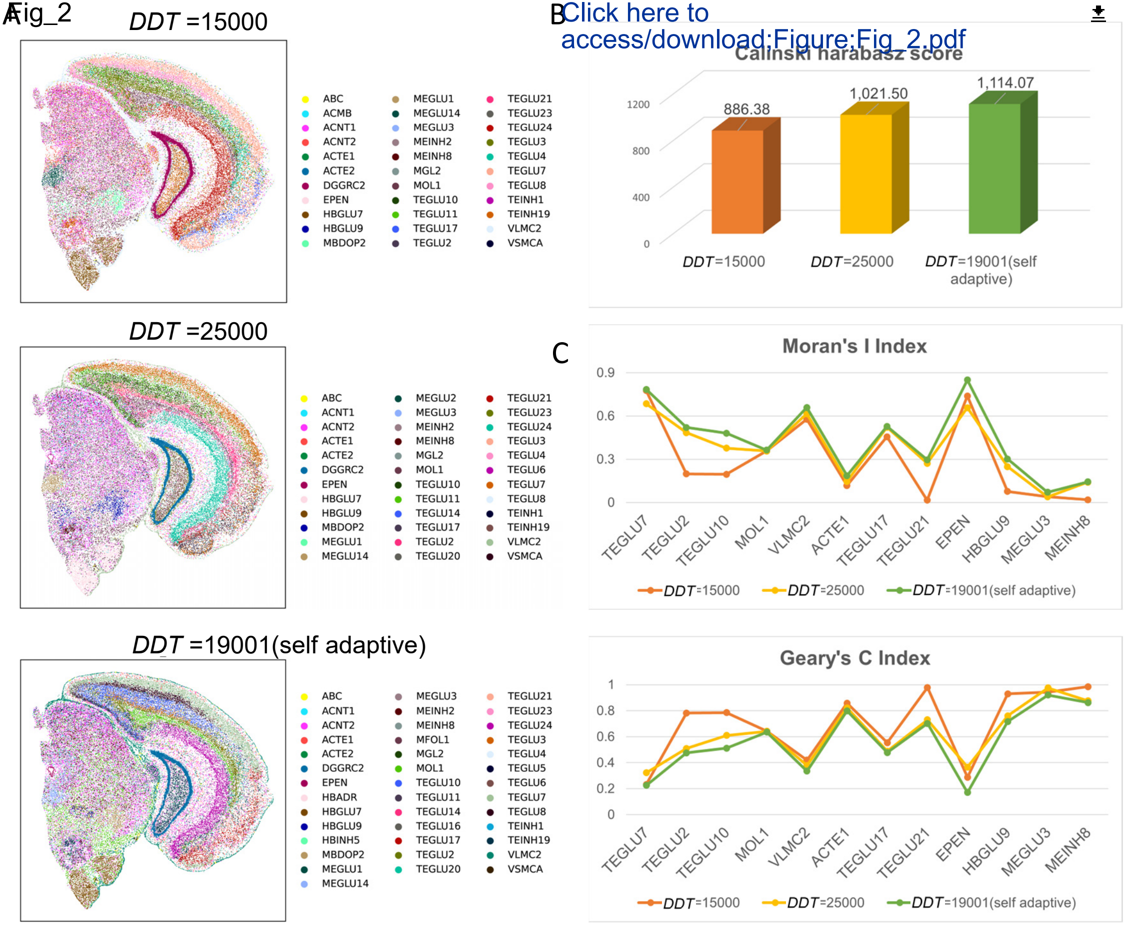

Fig_3

Click here to access/download;Figure;Fig_3.pdf

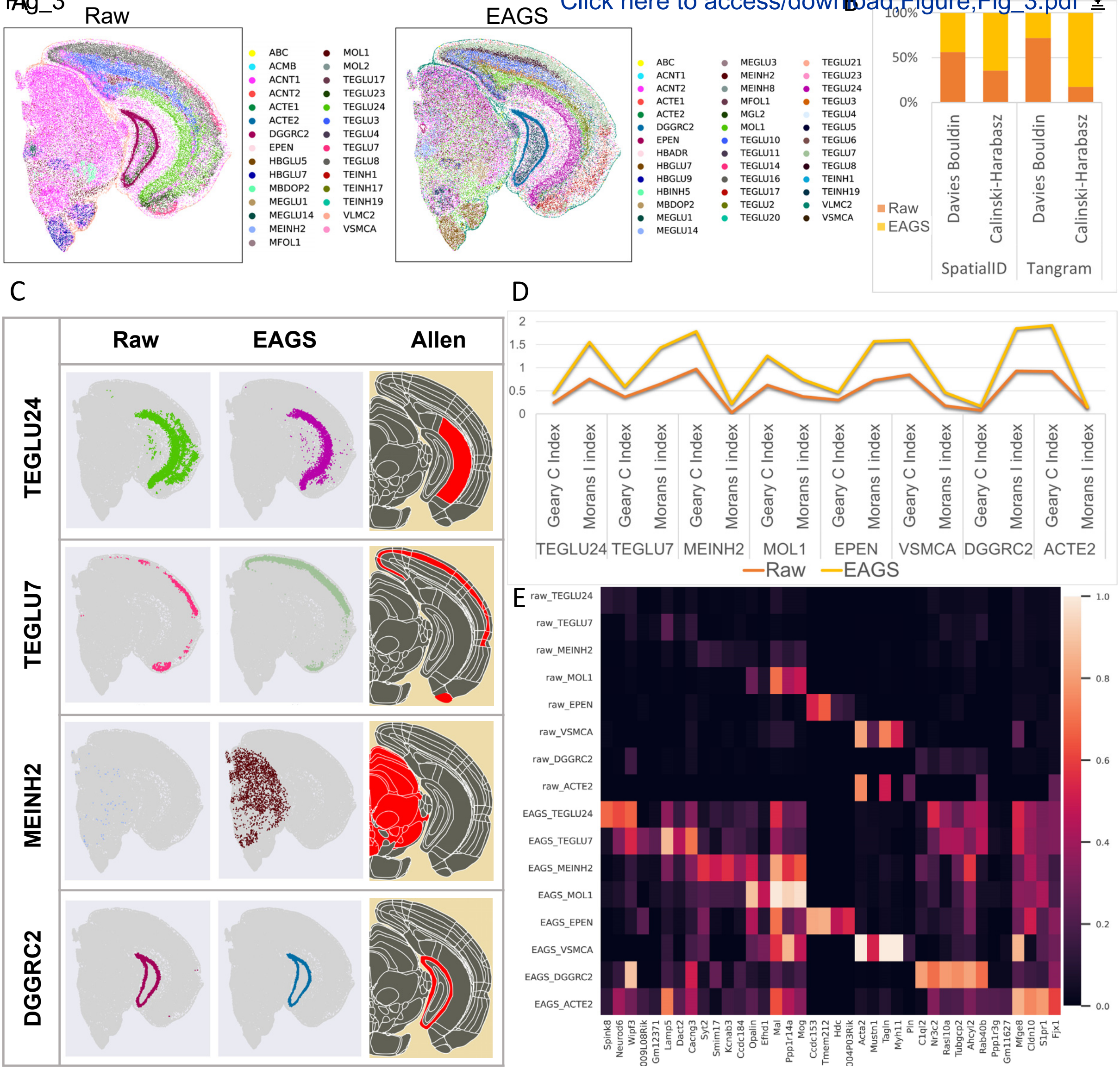

Fig_4 Click here to access/download;Figure;Fig_4.pdf

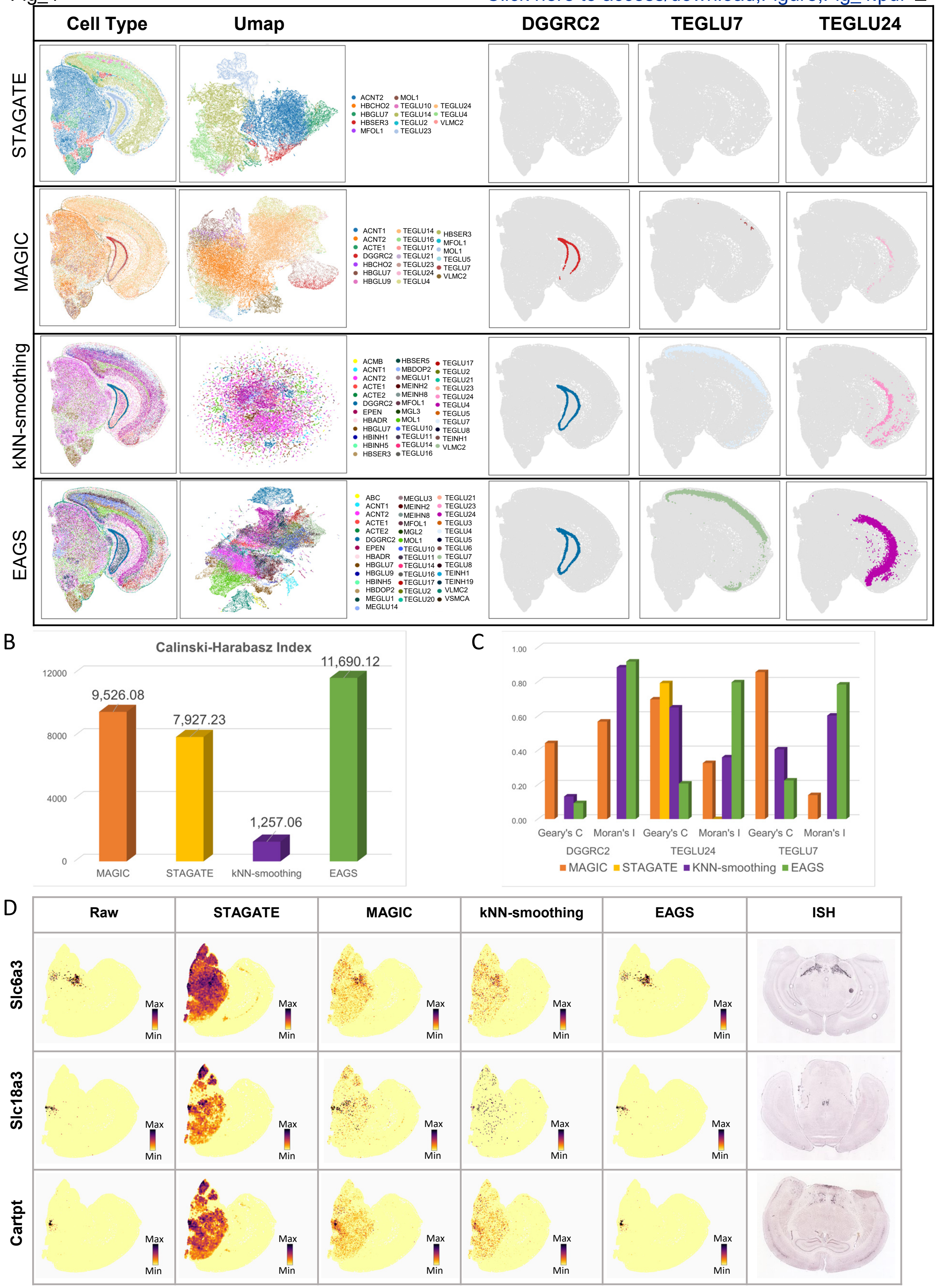

Fig_5

Click here to access/download;Figure;Fig_5.pdf

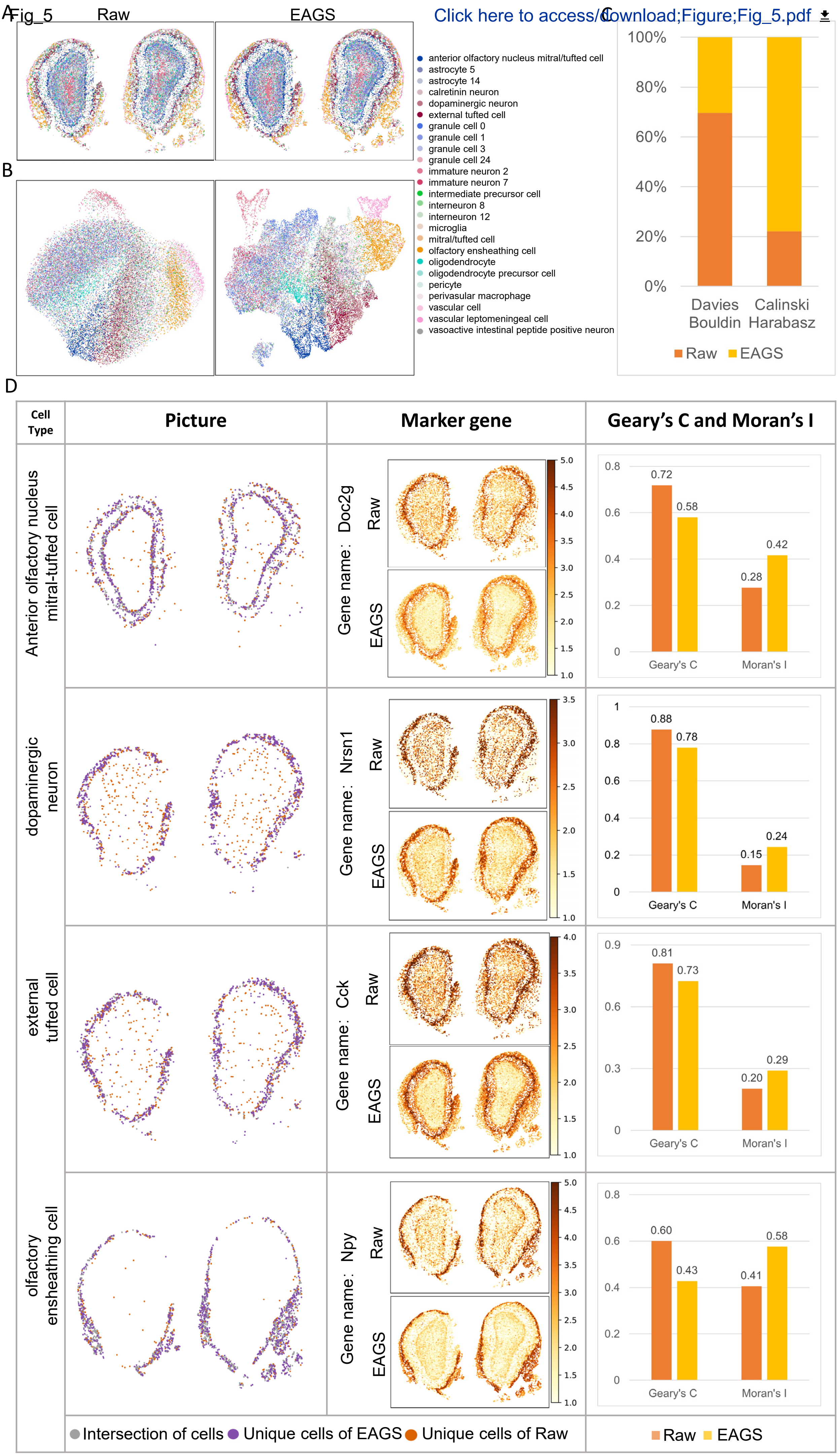

Click here to access/download
Supplementary Material
Responses.docx
