# Supplementary material for: EAGS: efficient and adaptive Gaussian smoothing applied to high-resolved spatial transcriptomics
Source: Gigascience. 2024 Feb 20;13:giad097. doi: 10.1093/gigascience/giad097 (PMC10939424; doi:10.1093/gigascience/giad097)
Supplement: giad097_Response_to_Reviewer_Comments_Original_Submission [file giad097_response_to_reviewer_comments_original_submission.pdf]

Author's Response To Reviewer Comments

[editor's note: a formatted version of this response letter, which is easier to read, is available as "supplemental material" at the end of the manuscript PDF]

Dear editor and reviewers:

First of all, we (the authors) would like to express our sincere gratitude to the editor and anonymous reviewers for their time and efforts given to our manuscript (Manuscript ID: GIGA-D-23-00147). Thanks a lot and best regards.

Secondly, it is worth pointing out that the reviewers' comments and suggestions have really and constructively helped us improve the quality and presentation of our manuscript much further. In light of their inspiring comments and suggestions, we have duly and carefully revised the manuscript, with the main changes highlighted in red color in the revised manuscript.

Thirdly, with many thanks to the reviewers, we would like to address their comments as below.

Authors Response to Comments of Editor

Comment: In addition, please register any new software application in the bio.tools and SciCrunch.org databases to receive RRID (Research Resource Identification Initiative ID) and biotoolsID identifiers, and include these in your manuscript (in the availability section).
Response: Many thanks are given to the editor for this comment.
We have registered the software application on bio.tools and SciCrunch.org respectively, and added the RRID and biotoolsID to the "Availability of Source Code and Requirements" section of the revised manuscript on Page 24, Lines 480-481.

Authors Response to Comments of Reviewer #1

Major comments:
Comment 1: More comprehensive and systematic benchmarking on large-scale synthetic dataset. In current manuscript, the comparisons were mainly performed on datasets where cell type annotations in previous researches were utilized. While the comparison results were appealing, to further demonstrate the strength of EAGS, it would be very insightful to incorporate more systematic benchmarking on synthetic data where "ground truth counts" are ready and more controllable. Recently, tools have been proposed to generate large-scale spatiomics data, e.g. scDesign3 (Nature Biotech 2023). In addition, the authors could also consider benchmarking with more spatial imputation methods such as SpaGE (NAR 2020) and classical scRNA-seq methods for example kNN-smoothing.
Response 1: Many thanks are given to the reviewer for the comment.
We have used ScDesign3 to generate a simulated dataset of the spatial transcriptomics mouse olfactory bulb. In addition to the existing comparison algorithms in the previous manuscript, we have also added the spatial imputation method SPCS [22] and classical scRNA-seq method kNN-smoothing [41] for comparison in the experiments on simulated and high-resolved datasets. Since SpaGE required single-cell transcriptome dataset as a reference and the datasets we used lacked such a reference, we have used the competitive method, SPCS, as an alternative.
The details can be seen on Page 1, Lines 23-24; Page 4, Lines 100-101, 112-113 and 115-116; Page 5, Lines 117, 120-121 and 129-132; Page 10, Lines 230-231 and 235-237; Page 11, Lines 238-243; Page 15, Lines 320-327; Page 16, Lines 329-336; Page 18, Lines 371-373; Page 19, Lines 380-391; Page 20, Lines 394-397; Page 21, Lines 398-407; Page 28, Lines 571-573 and 593-595; Page 29, Lines 598-602; Tabel 1 and Figure 4 of the revised manuscript.

22. Liu Y, Wang T, Duggan B, et al. SPCS: a spatial and pattern combined smoothing method for spatial transcriptomic expression. Brief Bioinform 2022;23(3):bbac116. doi:10.1093/bib/bbac116.
41. Wagner F, Yan Y, Yanai I. K-nearest neighbor smoothing for high-throughput single-cell RNA-Seq data

2. bioRxiv 2018;217737. doi: 10.1101/217737.

Comment 2: More detailed documentation and examples on software usage. The authors have already provided the source code of EAGS and the sample datasets to run, which is helpful. To make the software more user-friendly and enhance its potential impact, the authors might also consider improving the documentations and examples on Github page, by providing more description about function API, and step-by-step examples in notebooks (if applicable) for users to conveniently try EAGS on their own datasets and visualize the results.
Response 2: We sincerely thank the reviewer for the comment.
We have improved the documentation and usage examples on the Github page, provided instructions and interface instructions for EAGS input parameters, and provided 2 running modes and practical examples: 1. Quick run, and 2. Setting each parameter by users. It should be noted that we have changed the GitHub link as: https://github.com/STOmics/EAGS. We have also updated the link in "Availability of Source Code and Requirements" section of the revised manuscript. Please see Page 24, Line 475.

Comment 3: Discussions about limitation of EAGS and potential remedies. The key assumption in EAGS is about the similarity of "neighboring" cells in tissue spatial microenvironment. While this can hold true in many developing tissue system, in tumors where heterogeneity of microenvironment are significant, the assumption could be challenged. In such case, would the performance of EAGS be impaired? What are potential solutions, e.g. is it possible to fine-tuning the weights between ENM or SDM to allow for certain "cell-type-specific imputation"? The authors might include such discussions to further elucidate the scope of usage of EAGS.
Response 3: Thank the reviewer very much for such a comment.
The performance of EAGS will be affected in the tumor microenvironment with extremely heterogeneous microenvironment. We have added the potential solutions in "Discussions" section of the revised manuscript. Please see Page 24, Lines 458-465.

Minor comments:
Comment 4: In equation (4), does the "×" sign denotes matrix multiplication or simply elementwise operation?
Response 4: Thanks to the reviewer for the comment on the equation description.
We have corrected "×" in equation (4) to "·", which means the matrix multiplication. Please see Page 8, Lines 182-186 of the revised manuscript.

Comment 5: What is the rationale of defining DDT and therefore equation (8)? More explanations about the motivation here could be very insightful.
Response 5: Thanks to the reviewer for reasonable suggestions on the equation description.
We have added the explanations about the motivation of DDT in equation (8). Please see Page 9, Lines 207-214 of the revised manuscript.

Comment 6: For all the evaluation metric used, could authors note whether larger/smaller values indicate better performance?
Response 6: We thank the reviewer for the suggestion.
We have given more explanation about whether larger/smaller values indicate better performance. Please see Page 10, Line 237, Page 11, Lines 238-239, 246-248 and 256-257; Page 12, Lines 264-266 and 274-275 of the revised manuscript.

Comment 7: In Fig. 4F, it seems that less cells are expressing Cartpt after using EAGS? What is the reason?
Response 7: Many thanks are given to the reviewer for this comment.
The expression of the Cartpt gene is scattered in the original data heatmap, with more noise appearing, and the matching degree with Allen's ISH image is low. After EAGS smoothing, a lot of noise is eliminated, and more Cartpt genes are expressed in the correct cells, which is more degree with Allen's ISH image. The reduction of noise cells and the aggregation many Cartpt genes make the figure show such a result. We have given an explanation on Page 23, Lines 444-448 of the revised manuscript.

Authors Response to Comments of Reviewer #2

Comment 1: There's no specific comment to be made apart from the fact that authors could have used more similar tools for comparison.
Response 1: Sincerely thank the reviewer for the comment.
We have added the spatial imputation method SPCS [22] and classical scRNA-seq method kNN-smoothing [41] for comparison.
The details can be seen on Page 15, Lines 320-327; Page 16, Lines 329-336; Page 18, Lines 371-373; Page 19, Lines 380-391; Page 20, Lines 393-397; Page 21, Lines 398-407; Tabel 1 and Figure 4 of the revised manuscript.

22. Liu Y, Wang T, Duggan B, et al. SPCS: a spatial and pattern combined smoothing method for spatial transcriptomic expression. Brief Bioinform 2022;23(3):bbac116. doi:10.1093/bib/bbac116.
41. Wagner F, Yan Y, Yanai I. K-nearest neighbor smoothing for high-throughput single-cell RNA-Seq data 2. bioRxiv 2018;217737. doi: 10.1101/217737.

Comment 2: From Fig2-5, legend font can be increased. It's blurry and not visible properly.
Response 2: Thank the reviewer for such a comment.
We have enlarged the font of the legend in Figures 2-5 in this revision. In addition, we have provided vector figures in PDF format to clearly show the details.

Comment 3: Why only ISH data is analyzed when lots of ISS datasets are also available. Would EAGS perform equally well on ISS data like from 10X Xenium?
Response 3: Many thanks are given to the reviewer for raising the comment.
We have used the ISS datasets based on in situ capture as the test set in our experiments, such as spatial transcriptomics datasets of mouse brain and olfactory bulb. Since these ISS datasets lacked ground truth, and Allen's ISH dataset is a published standard dataset, such we have used Allen's ISH dataset as a reference for evaluation of different imputation methods.
EAGS is an imputation method for high-resolved spatial transcriptomics data. Given that 10X Xenium has high resolution, EAGS has the potential to be applied on this ISS data. We will also explore improvements and more applications of EAGS in next work.

Finally, we (the authors) would like to express thanks again sincerely to the editor and anonymous reviewers for their time and efforts spent in handing the manuscript, as well as providing us many constructive comments for improving further the presentation and quality of this manuscript.
