# Supplementary material for: EAGS: efficient and adaptive Gaussian smoothing applied to high-resolved spatial transcriptomics
Source: Gigascience. 2024 Feb 20;13:giad097. doi: 10.1093/gigascience/giad097 (PMC10939424; doi:10.1093/gigascience/giad097)
Supplement: giad097_Response_to_Reviewer_Comments_Revision_1 [file giad097_response_to_reviewer_comments_revision_1.pdf]

Author's Response To Reviewer Comments

Dear editor

First of all, we (the authors) would like to express our sincere gratitude to the editor and reviewers for their time and efforts given to our manuscript (Manuscript ID: GIGA-D-23-00147R1). Thanks a lot, and best regards.
With many thanks, we would like to address the comments as below.

Comment 1: Please include a citation to your new GigaDB dataset (including the DOI link) to your reference list, and cite this by number from in the data availability section.
Response: We have added a citation of GigaDB dataset (including the DOI link) in the reference list and updated the data availability section.

Comment 2: Please cite the Zenodo and STOMICs datasets analogous to the gigaDB example above (i.e. each dataset should get its own entry in the bibliography, and cite them by number from the main text rather than including the URL/doi directly in the main text). Mention accession numbers where applicable.
Response: We have added the entries in the references for each of the Zenodo and STOMICs datasets cited in the Data Availability section with the login number.

Comment 3: Please also move any other in-text URLs for data sources to the bibliography and cite them by number in the text (similar to the GigaDB reference - please refer to our citation guidelines.
Response: We have moved the URLs to the References section and added the citation number.

Comment 4: please double check that all relevant accession numbers and data citations are included. (STOmicsDB_Sample: STSA0000207, STOmicsDB_Sample: STSA0000248)
Response: We have checked all relevant accession numbers and data citations.

Comment 5: The CNGB accession CNP0002966 is redundant and non-functional, as far as I understand - please remove this.
Response: We have removed this accession.

Comment 6: Please structure your abstract ("Background - Findings – Conclusions").
Response: We have structured the abstract section as ("Background - Findings – Conclusions").

Comment 7: At this stage, you can remove all highlighting/tracking of changes in red, that was made for the purpose of peer review.
Response: We have removed the highlighting/tracking of changes.

Comment 8: Please submit editable source files (WORD or LaTex, whatever you used - at the moment, we only have a PDF).
Response: We have submitted the source files of WORD.

Comment 9: If you cited preprints, please check if they are now published in a reviewed journal - if so, please cite the journal article instead. (But it's fine to cite preprints if they are not published in peer reviewed form yet).
Response: We have rechecked the latest publication status of preprints and have ensured that none of the preprint articles cited in the latest uploaded revision have been published in a journal.

Comment 10: Please also ensure that your revised manuscript conforms to the journal style, which can be found in the Instructions for Authors on the journal homepage.
Response: We have revised the manuscript to ensure that the revised manuscript conforms to the journal style.

Finally, we (the authors) would like to express thanks again sincerely to the editor and reviewers for their

time and efforts spent in handing the manuscript, as well as providing us many constructive comments for improving further the presentation and quality of this manuscript.
