# Supplementary material for: EAGS: efficient and adaptive Gaussian smoothing applied to high-resolved spatial transcriptomics
Source: Gigascience. 2024 Feb 20;13:giad097. doi: 10.1093/gigascience/giad097 (PMC10939424; doi:10.1093/gigascience/giad097)
Supplement: giad097_Reviewer_1_Report_Original_Submission — Peijie Zhou -- 7/2/2023 [file giad097_reviewer_1_report_original_submission.pdf]

Reviewer Report

Title: EAGS: efficient and adaptive Gaussian smoothing applied to high-resolved spatial transcriptomics

Version: Original Submission **Date:** 7/2/2023

Reviewer name: Peijie Zhou

Reviewer Comments to Author:

In this manuscript, the authors proposed the method called EAGS to enhance signals in spatial transcriptomics data using adaptive Gaussian smoothing approach. The method imputes gene expression counts of a certain cell by utilizing the information of "neighbor cells" in terms of both spatial coordinates and gene expression similarity , and a designated Gaussian kernel was adopted for imputation. The authors demonstrate the superb performance of EAGS in mouse brain and mouse olfactory bulb datasets.Overall, the manuscript addresses the meaningful technical issue in spatial omics data analysis, and the method has the promise to serve as a useful tool in data preprocessing. The methodology part of manuscript is well-described. In the meantime, I have some suggestions below for authors to consider in order to further improve the manuscript.1. More comprehensive and systematic benchmarking on large-scale synthetic dataset. In current manuscript, the comparisons were mainly performed on datasets where cell type annotations in previous researches were utilized . While the comparison results were appealing, to further demonstrate the strength of EAGS, it would be very insightful to incorporate more systematic benchmarking on synthetic data where "ground truth counts" are ready and more controllable. Recently, tools have been proposed to generate large-scale spatiomics data, e.g. scDesign3 (Nature Biotech 2023). In addition, the authors could also consider benchmarking with more spatial imputation methods such as SpaGE (NAR 2020) and classical scRNA-seq methods for example kNN-smoothing.2. More detailed documentation and examples on software usage. The authors have already provided the source code of EAGS and the sample datasets to run, which is helpful. To make the software more user-friendly and enhance its potential impact, the authors might also consider improving the documentations and examples on Github page, by providing more description about function API, and step-by-step examples in notebooks (if applicable) for users to conveniently try EAGS on their own datasets and visualize the results.3. Discussions about limitation of EAGS and potential remedies. The key assumption in EAGS is about the similarity of "neighboring" cells in tissue spatial microenvironment . While this can hold true in many developing tissue system, in tumors where heterogeneity of microenvironment are significant, the assumption could be challenged. In such case, would the performance of EAGS be impaired? What are potential solutions, e.g. is it possible to fine-tuning the weights between ENM or SDM to allow for certain "cell-type-specific imputation"? The authors might include such discussions to further elucidate the scope of usage of EAGS.Minor:1. In equation (4), does the "x" sign denotes matrix multiplication or simply elementwise operation?2. What is the rationale of defining DDT and therefore equation (8)? More explanations about the motivation here could be very insightful.3. For all the evaluation metric used, could authors note whether

larger/smaller values indicate better performance?4. In Fig. 4F, it seems that less cells are expressing Carpt after using EAGS? What is the reason?

Level of Interest

Please indicate how interesting you found the manuscript: Choose an item.

Quality of Written English

Please indicate the quality of language in the manuscript: Choose an item.

Declaration of Competing Interests

Please complete a declaration of competing interests, considering the following questions:

- Have you in the past five years received reimbursements, fees, funding, or salary from an organisation that may in any way gain or lose financially from the publication of this manuscript, either now or in the future?
- Do you hold any stocks or shares in an organisation that may in any way gain or lose financially from the publication of this manuscript, either now or in the future?
- Do you hold or are you currently applying for any patents relating to the content of the manuscript?
- Have you received reimbursements, fees, funding, or salary from an organization that holds or has applied for patents relating to the content of the manuscript?
- Do you have any other financial competing interests?
- Do you have any non-financial competing interests in relation to this paper?

If you can answer no to all of the above, write 'I declare that I have no competing interests' below. If your reply is yes to any, please give details below.

I declare that I have no competing interest

I agree to the open peer review policy of the journal. I understand that my name will be included on my report to the authors and, if the manuscript is accepted for publication, my named report including any attachments I upload will be posted on the website along with the authors' responses. I agree for my report to be made available under an Open Access Creative Commons CC-BY license (http://creativecommons.org/licenses/by/4.0/). I understand that any comments which I do not wish to be included in my named report can be included as confidential comments to the editors, which will not be published.

Choose an item.

To further support our reviewers, we have joined with Publons, where you can gain additional credit to further highlight your hard work (see: https://publons.com/journal/530/gigascience). On publication of this paper, your review will be automatically added to Publons, you can then choose whether or not to claim your Publons credit. I understand this statement.

Yes Choose an item.
